# Ex vivo modelling of human colorectal cancer liver metastasis by normothermic machine perfusion

**DOI:** 10.1186/s12943-025-02430-7

**Published:** 2025-10-21

**Authors:** Manuel Trebo, Thomas Maurer, Felix J. Krendl, Stefan Salcher, Agnieszka Martowicz, Theresa Hautz, Sieghart Sopper, Arno Amann, Benno Cardini, Lukas H. Poelsler, Anna Mair, Julia Hofmann, Andras T. Meszaros, Martin Hermann, Michael Günther, Steffen Ormanns, Zlatko Trajanoski, Stefan Schneeberger, Dominik Wolf, Rupert Oberhuber, Andreas Pircher

**Affiliations:** 1 Department of Internal Medicine V, Hematology and Oncology, Comprehensive Cancer Center Innsbruck (CCCI), Austrian Comprehensive Cancer Network (ACCN), Tyrolean Cancer Research Institute (TKFI), Medical University of Innsbruck, Innsbruck, Austria; 2 Department of Visceral, Transplant and Thoracic Surgery, Center of Operative Medicine, organLife Laboratory and D. Swarovski Research Laboratory, Medical University of Innsbruck, Innsbruck, Austria; 3 Tyrolpath Obrist Brunhuber GmbH, Zams, Austria; 4 Innpath Institute of Pathology, Tirol Kliniken, Innsbruck, Austria; 5 Institute of General Pathology, Medical University Innsbruck, Innsbruck, Austria; 6 Biocenter, Institute of Bioinformatics, Medical University of Innsbruck, Innsbruck, Austria

**Keywords:** Liver transplantation, Human cancer model, Colorectal cancer metastasis, *Ex vivo*, Normothermic machine perfusion, Single-cell mapping, Spatial transcriptomics.

## Abstract

**Background:**

Colorectal cancer liver metastasis (CRLM) is associated with poor survival, primarily due to acquired therapy resistance. While novel therapies arise, translation is limited by the lack of tumor models accurately representing dynamic microenvironmental interplay. Here, we show that ex vivo normothermic machine perfusion (NMP) offers a novel preclinical framework to study the intratumoral dynamics of CRLM biology.

**Methods:**

Six resected metastatic human livers were preserved for two days and subjected to multi-omic profiling of serially sampled adjacent liver and metastatic tissue using single-cell RNA sequencing (scRNA-seq) and spatial transcriptomics (ST). Tissue integrity was assessed and cross-validated by immunofluorescence (IF), high-resolution respirometry (HRR) and flow-cytometry.

**Results:**

NMP was successfuly applied to metastatic livers with minimal surgical adaptations, preserving both intrinsic hepatic properties and tissue viability over an extended duration. Single-cell and spatial mapping confirmed preservation of CRLM phenotypic properties and demonstrated high clinical translatability by applicability of the intrinsic epithelial consensus molecular subtypes to metastasis. Spatially deconvoluted pathway activities reflected functional tissue-microenvironments. Transcriptomic profiles – including those of tumor-associated myeloid cells – were preserved during NMP. Finally, we demonstrate tumor-associated myeloid cell persistence as a driver of disease progression and poor survival in colorectal cancer.

**Conclusion:**

Our findings represent the basis for future innovative applications adopting NMP in the context of CRLM, providing a new preclinical tumor model avenue.

**Graphical Abstract:**

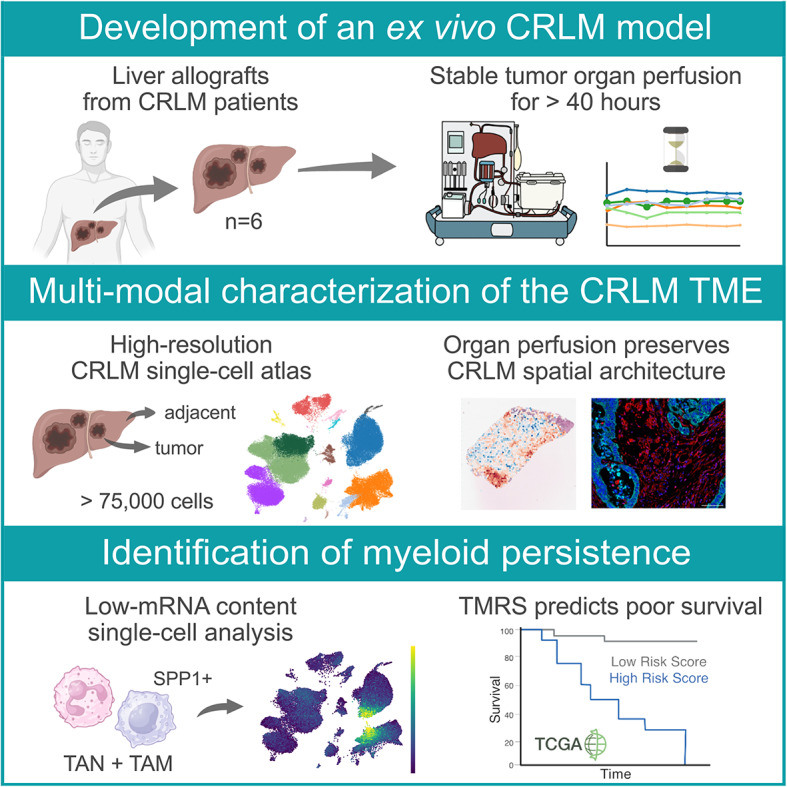

**Supplementary Information:**

The online version contains supplementary material available at 10.1186/s12943-025-02430-7.

## Background

Colorectal cancer (CRC) represents one of the most significant global health burdens, accounting for nearly one million deaths annually [1]. Despite advances in diagnosis and therapy patients with disease progression and metastatic dissemination face poor survival. Metastatic CRC (mCRC) is associated with a dismal five-year survival rate of approximately 15%. The liver is the primary target for metastasis [2]. At present, surgery offers the only curative treatment for mCRC but is often limited by metastatic spread, anatomical constraints, or poor functional liver reserve [3]. Recurrence rates following successful metastatic resection or local treatment exceed 60% within three years. This underscores the limitations of current therapeutic strategies [4]. Non-surgical interventions – including chemotherapy, targeted therapy, and immunotherapy – often show limited effect in the advanced metastatic setting, with low response rates and poor median progression-free survival [5]. These poor outcomes arise from key biological characteristics inherent to colorectal liver metastasis (CRLM), including metastatic dormancy mediating tumor cell survival, extensive intra- and inter-tumoral heterogeneity, and a highly immunosuppressive microenvironment that limits the efficacy of immunotherapies [6–8]. The lack of preclinical CRLM models that fully recapitulate the complex biology of metastatic disease and its response to therapy hampers the advancement of novel treatment strategies. While advanced mCRC research platforms, such as engineered mouse models and patient-derived organoid systems [6, 9], offer valuable insights, they often miss key features of CRLM, including its complex three-dimensional architecture, stromal and vascular heterogeneity, dynamic tumor microenvironment (TME) interactions, immunological ecosystems, inter-patient heterogeneity, the impact of the microbiome and previous treatments [9–13]. This critical gap between experimental models and the clinical reality is underscored by the fact that nearly 90% of drug candidates entering Phase I trials ultimately fail [14]. This illustrates the urgent need for more representative CRLM models.

Ex vivo normothermic machine perfusion (NMP) of organs or organ parts may serve as a human-like model and promising platform for therapy testing. Originally developed and now routinely applied in solid organ transplantation to extend and improve preservation, NMP simulates body-like conditions by providing oxygen and essential nutrients at normothermia. Compared to standard cold storage, NMP ameliorates the preservation induced damage and allows to assess organs during preservation [15–17]. Beyond its role in transplantation, NMP presents unique potential as a modeling platform for various disease states [18–20]. Recent studies have demonstrated successful long-term perfusion of diseased livers, including a tumor-bearing liver preserved for 17 days [21–24]. These pioneering efforts indicate the capacity of ex vivo NMP to retain the phenotypic properties of diseased livers, including those with primary tumors or metastatic lesions. In order to make this clinically meaningful and reproducible, however, simple and more standardized protocols are needed.

To address these limitations and capitalize on the potential of NMP, we repurposed whole or partial human livers from six patients with CRLM and perfused these organs using the OrganOx^®^ Metra^®^ system for two days. During this period, we employed multi-modal single-cell mapping of both adjacent liver and tumor regions before and after perfusion to confirm phenotypic properties and evaluate their temporal stability. The preservation of tissue architecture and spatial context was assessed using spatial transcriptomics (ST) and multiplex immunofluorescence (IF). Our findings establish an innovative platform for investigating CRLM biology, drug, and immunotherapy testing under physiologically relevant conditions. By capturing the complexity of human metastatic disease, this ex vivo model bridges the gap between advanced in vitro models and traditional in vivo approaches, offering a crucial leap in translational cancer research.

## Results

### Establishment of an ex vivo colorectal cancer liver metastasis model using normothermic machine perfusion

**Fig. 1:**
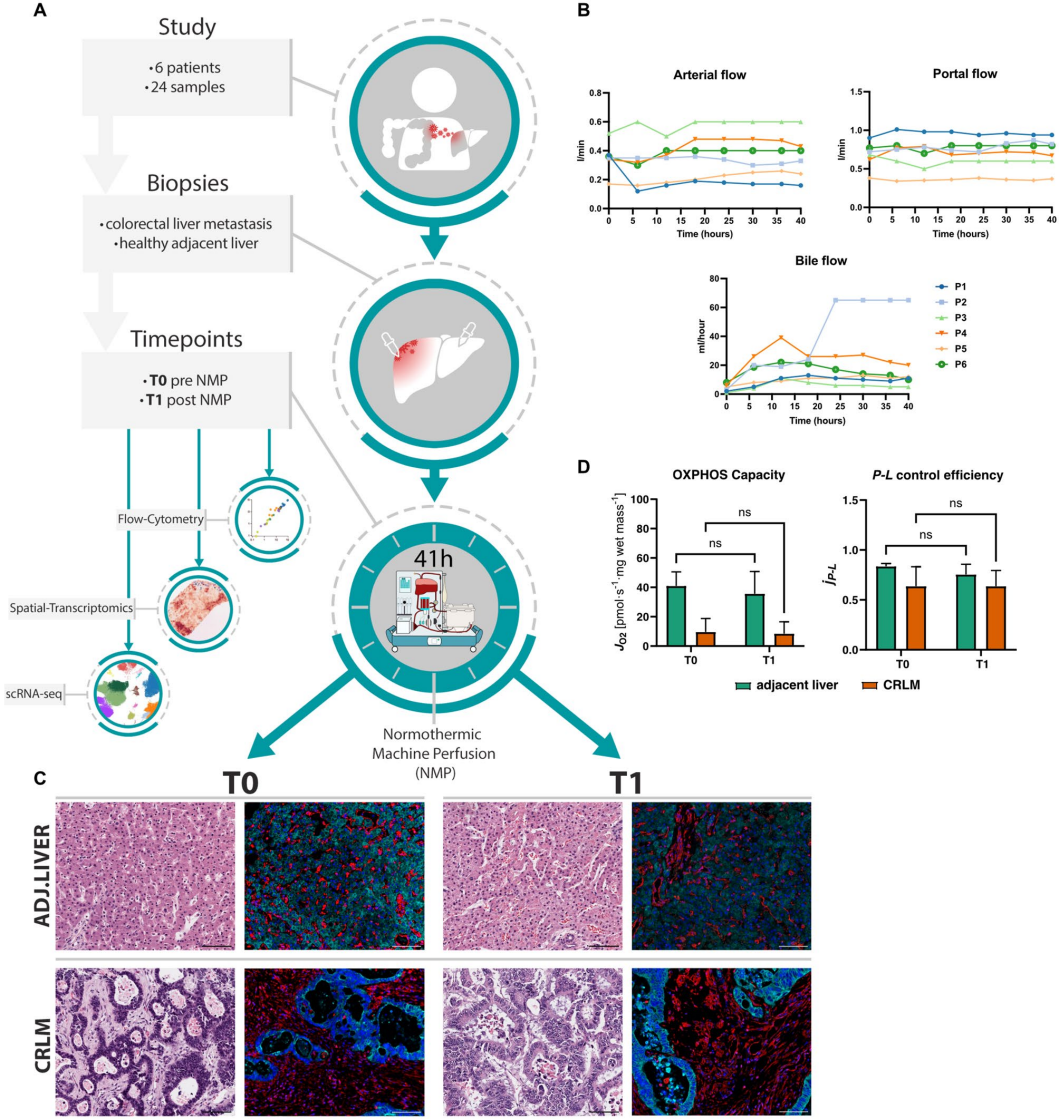
NMP of partial- or whole livers with CRLM lesions as a novel tumor model **A** Graphical overview of the study workflow. **B** Flow and pressure curves, as well as bile flow of livers during NMP are shown. **C** H&E stainings and multiplex IF images of tissue integrity markers (Vimentin - red, panCK - turquoise, DAPI - blue) presented before (T0) and after (T1) NMP of corresponding adjacent liver and CRLM tissue. Images are displayed at ×20 magnification (scale bar: 100 μm). **D** HRR analysis for evaluation of the capacity of mitochondrial oxidative phosphorylation (OXPHOS) and efficiency of ATP production (P-L control efficiency)

In total, six hepatectomy specimens were obtained from patients undergoing liver resection (LR) or liver transplantation (LT) for CRLM (patient characteristics are depicted in Table S1). An overview of the overall study set-up, including the central analysis platforms applied, is displayed in Fig. 1A. The median age of the cohort was 49.5 years (39–70). In all six patients the primary tumor (CRC) was left-sided, BRAF^wt^, and mismatch repair proficient - microsatellite stable (MSS). In five out of six patients the primary had been resected upon neoadjuvant therapy. In one patient with rectal cancer, the primary tumor was no longer detectable (ycT0) following six months of chemotherapy. All patients had received systemic therapy before LR or LT (Table S1). The response of CRLM to chemotherapy was evaluated and classified according to Tumor Regression Grade (TRG) system developed by Rubbia-Brandt et al. [25], which assesses the extent of fibrosis and residual tumor cells in resected liver specimens (Table S1). In two cases a right hepatectomy was performed, in one case a patient was treated with a left hepatectomy. In three cases, a hepatectomy and LT for unresectable bilobar CRLM was performed. The details of the surgical procedure and machine perfusion are displayed in Fig. S1A-F. The median NMP duration was 41 h (38–64). Machine perfusion was uneventful with stable pressure and flow (Fig. 1B – top), and consistent bile production, indicating sustained liver function and viability (Fig. 1B – bottom). Lactate levels and pH remained stable over the course of the perfusion (Fig. S1G). All livers displayed glucose utilization, and transaminase levels remained within acceptable ranges (Fig. S1G). Throughout the ex vivo perfusion experiment, tissue viability remained high, and the architectural organ integrity was maintained (Fig. 1C). Furthermore, high resolution respirometry (HRR) confirmed bioenergetic integrity over the course of perfusion in all organs (Fig. 1D). An additional tumor liver subjected to extended NMP further confirmed the platform’s capacity to sustain stable perfusion for up to 144 h, with gradual deterioration thereafter (Fig. S2A-C). Collectively, our results demonstrate that NMP can be applied to resected livers with CRLM. The intrinsic properties of both tumor and liver tissue remain stable over an extended preservation period.

### A cell atlas of CRLM and adjacent liver tissue during ex vivo NMP

**Fig. 2:**
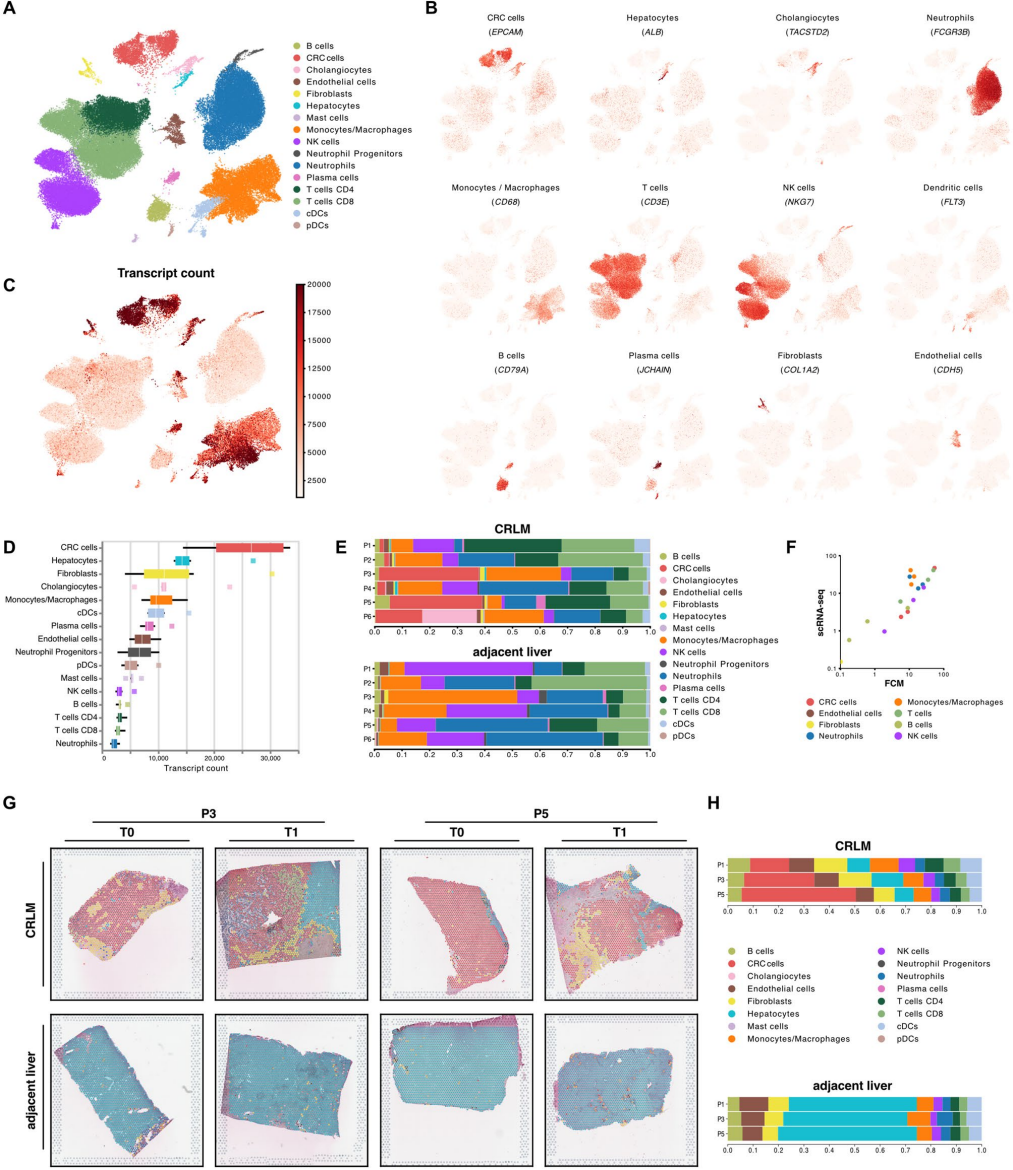
In-depth cellular profiling of an ex vivo CRLM model (**A**) Uniform Manifold Approximation and Projection (UMAP) plot of 75,075 high quality single-cells with color-coded cell types. **B** UMAP plot showing color-coded canonical marker expression used for cell-type annotation. **C** UMAP and (**D**) boxplots showing total transcript counts per cell-type (**E**) Barplots showing cell type percentages in scRNA-seq data per patient and tissue (CRLM – top or adjacent liver – bottom) including both time points (T0 and T1). **F** Proportional correlation of selected cell types between flow-cytometry and scRNA-seq data. **G** Cell2Location deconvoluted images of adjacent liver and CRLM tissues showing the predicted most dominant cell type per spot and (**H**) the mean percentage of cell types of both time points

To chart the cellular ecosystem of CRLM in our ex vivo perfused model, we analysed 75,075 high-quality cells from six patients including both, adjacent liver and CRLM tissues, before (T0) and after (T1) NMP by whole transcriptome single-cell RNA sequencing (scRNA-seq, Fig. 2A). Canonical marker-based annotation revealed the presence of epithelial tumor cells (*EPCAM*), hepatocytes (*ALB*), cholangiocytes (*TACSTD2*), fibroblasts (*COL1A2*) and endothelial cells (*CDH5*) but also immune cells, such as neutrophils (*FCGR3B*), monocytes/macrophages (*CD68*), plasmacytoid dendritic cells (CLEC4C), conventional dendritic cells (FLT3), T cells (*CD3E*), NK cells (*NKG7*), B cells (*CD79A*) and plasma cells (*JCHAIN*) (Fig. 2B, Fig. S3A). Cell type annotations were validated by their top expressed marker genes (Table S2). Tumor cells (mCRC cells) were clearly distinguishable from other cell types based on hallmark tumor genes and overall elevated transcript counts (Fig. 2C and D). Conversely, neutrophils exhibited the lowest median mRNA transcript levels, however the protocol for low-mRNA content cells using the BD Rhapsody scRNA-seq platform [26, 27] readily enables their inclusion in the subsequent analyses.

Cellular composition showed considerable inter-patient heterogeneity (Fig. 2E, Fig. S3B. Table S3), which primarily resulted from mCRC cells rather than from the non-cancerous cell compartments within the TME (Fig. S3C). Focusing on the TME, T cells (33.7%) were the most abundant cell type, followed by neutrophils (21.9%), which we previously described to be the predominant cell population in healthy donor livers [28]. While NK cells were less abundant in the tumor vs. adjacent liver tissue, CD4^+^ T cells showed an expansion when compared to the adjacent tissue (Fig. 2E).

Flow-cytometry analyses of three corresponding tumor samples demonstrated high concordance with the relative leukocyte and epithelial cell proportions as detected by scRNA-seq (Fig. 2F). While these results confirmed suitability of our model, hepatocytes appeared to be underrepresented in the generated scRNA-seq dataset. This is possibly resulting from bead-exclusion, a phenomenon inherent to the microwell-based BD Rhapsody platform [28]. To outweigh this shortcoming and to characterize the spatial organization and cellular composition within tumor- and the corresponding adjacent tissue, we applied ST in samples from three representative patients. We cross-referenced our findings by performing a signature-based approach using Cell2Location to deconvolute resulting ST spots and estimate their cell proportions (see *Methods*) [29]. These analyses confirmed that our ex vivo CRLM model depicts all relevant immune cell populations, as well as hepatocytes, fibroblasts, endothelial cells, and tumor cells consistently across all patients (Fig. 2G and H, Fig. S3D).

### mCRC cells in the liver display typical metastatic characteristics

**Fig. 3:**
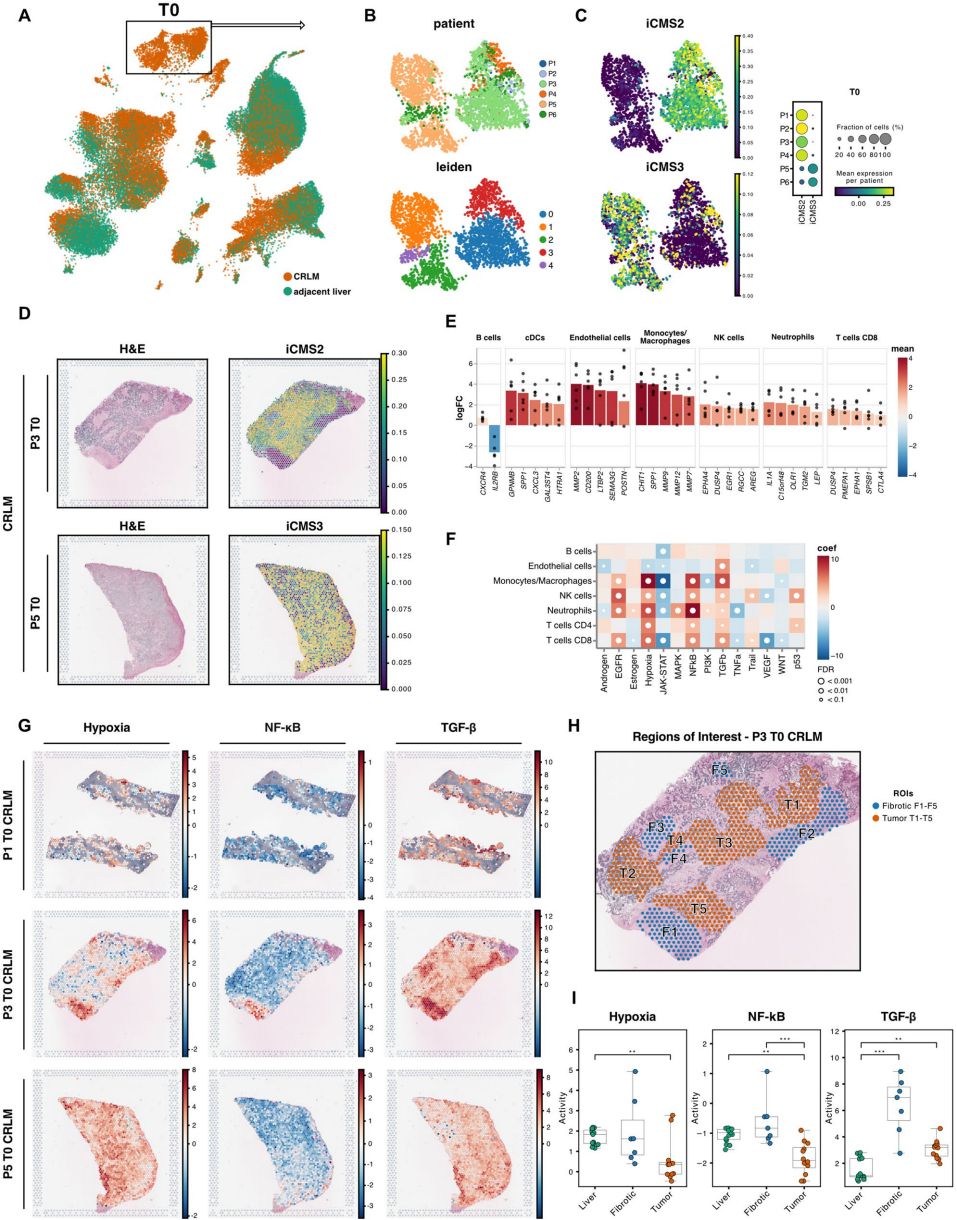
Transcriptomic landscape of CRLM at perfusion start (T0) (**A**) UMAP color-coded by tissue-type at T0. **B** UMAPs of subclustered mCRC cells color-coded by patient (top) and leiden-clusters (bottom). **C** UMAPs (left panel) and dotplot (right panel) showing iCMS2 and iCMS3 signature scores divided by patient. **D** Spatial images showing H&E stainings (left panel) and signature expression of iCMS2 and iCMS3 (right panel) of CRLM tissue from patient 3 and 5 at T0. **E** Top DEGs per cell type sorted by logFC between adjacent liver and CRLM at T0. Each dot represents the mean of an individual patient (*n* = 6) (**F**) Differential activation based on ProgenY pathways per cell type using DEGs between tissues at T0. Heatmap shows inferred activation deviation from the mean, white dots show thresholds for false-discovery rates (FDR). **G** Selected pathway activities (Hypoxia, NF-κB and TGF-β) within CRLM tissues of patients calculated based on the ProgenY network at T0. **H** Spatial tumor image of patient 3 at T0 showing pathologist annotations. **I** Mean pathway activities per ROI compared between CRLM and adjacent liver tissue at T0

In order to provide relevant clinical translatability, a novel cancer model should accurately depict the known and distinct metastatic phenotypes [30]. Thus, we first focused on relevant aspects of cancer biology at baseline (T0, Fig. 3A) and investigated mCRC cells in more detail (Fig. 3B). Given the inherent heterogeneity of the cancer cell compartment, we initially sought to characterize and confirm their malignant nature. We therefore analysed copy number variations (CNVs) and genomic alterations involving gains or losses of chromosomal regions, using InferCNV. Clustering in CNV space revealed inter-patient differences, indicating distinct CNV programs across individuals (Fig. S4A). Patient 5 exhibited a markedly distinct CNV profile contrasting other patients, suggesting a unique genomic landscape. Cancer cells, in general, displayed higher CNV scores, reflecting increased genomic instability.

Next, we assessed the tumor epithelial cell states of different mCRC cell transcriptomes through advanced binary classification, categorizing mCRC cells from primary MSS tumors into two distinct intrinsic-consensus molecular signatures (iCMS) [31], each linked to specific phenotypic implications. iCMS scoring using corresponding gene sets stratified patients into iCMS2 and iCMS3 classes. Our findings confirm applicability of the iCMS classification to metastatic settings (Fig. 3C). Patients 1–4 showed almost exclusively iCMS2 features, while patients 5 and 6 revealed a clear predominance of the iCMS3 phenotype. These patient-specific iCMS patterns were confirmed within their respective ST specimens (Fig. 3D). iCMS signatures clearly overlapped with tumor regions manually curated by two independent pathologists (Fig. 3D – left). Furthermore, we validated the applicability of iCMS classification to metastatic settings by analyzing a large publicly available dataset of mCRC patients [32]. After subsetting and reclustering of mCRC cells, we found a comparable distinction into two molecular subpopulations with differential enrichment of iCMS2 and iCMS3 signatures. Our findings suggest iCMS applicability to metastatic settings (Fig. S4B).

Recent work revealed that mCRC spans over a large range of differentiation states, representing its inherent heterogeneity [33]. To evaluate this in our model, we projected these functional phenotypes of non-canonical and canonical metastatic programs on our mCRC cells (Fig. S4C-D). We observed that similar expression patterns corresponded well with subgroups identified from unsupervised Leiden clustering (Fig. 3B). While most mCRC cells appeared to adopt more canonical phenotypes, patient 5 displayed a non-canonical neuroendocrine and osteoblast state. This finding corresponded with our spatial dataset (Fig. S4E). Notably, we observed the presence of multiple heterogeneous metastatic signatures within patients (Fig. S4F).

Taken together, our multi-omic characterization of mCRC lesions highlights cancer cell complexity and suggests the compatibility of the primary iCMS classification to metastasis.

### CRLM tissue displays features of a functional mCRC TME

Building on the accurate representation of metastatic phenotypes, we next explored whether the gene expression of the obtained CRLM tissues capture features of a functional mCRC TME. First, we compared cell-type-specific gene expression differences between CRLM and adjacent liver tissues before perfusion start (T0). We observed markedly altered gene expression across diverse cell populations, with pronounced shifts in monocytes/macrophages (1558 differentially expressed genes (DEGs)) and endothelial cells (244 DEGs, Fig. 3E, Table S4). Within the top DEGs we identified dysregulated genes frequently linked to aggressiveness and treatment resistance, such as *SPP1* in monocytes/macrophages or *MMP2* in endothelial cells [34, 35].

Next, we assessed the connection between expression profiles and distinct cellular processes through inferring pathway analysis using the ProgenY network. We identified several significantly dysregulated activities among distinct cell types, including NF-κB, TGF-β or Hypoxia pathways (Fig. 3F). Persistent NF-κB may fuel chronic inflammation in mCRC, promote cancer cell survival, growth and angiogenesis [36, 37]. Aberrant TGF-β and Hypoxia signalling in the mCRC TME also play synergistic roles directly linked towards therapeutic resistance and tumor progression [38–40]. Monocytes/macrophages and neutrophils were characterized by particularly prominent Hypoxia and NF-κB signalling. Gene expression activities of monocytes/macrophages in the tumor were overall more significantly altered. The spatial representation of these findings were validated using matched spatial data (Fig. 3G, Fig. S5A). To ensure accurate spatial quantification within heterogeneous tissue samples, we defined regions of interest (ROIs) curated by matching with tumor, fibrotic, and healthy zones in corresponding hematoxylin and eosin (H&E) slides (Fig. 3H, Fig. S5B, see *Methods*). The ROI characteristics corresponded well with the estimated dominant cell population from Cell2Location (Fig. 2G). Interestingly, areas of TGF-β and Hypoxia signalling overlapped within fibrotic regions and monocyte/macrophage presence. Emerging evidence links direct interactions between these cell types in hypoxic areas to the exacerbation of the CRC microenvironment [34]. Further quantification of ProgenY activity across ROIs revealed pronounced upregulation of TGF-β and NF-κB signalling. The strongest activation was found in peritumoral fibrotic areas. This aligns well with expression patterns from single-cell data and suggests that TGF-β and NF-κB signalling are primary drivers of this process (Fig. 3F and G). Conversely, hypoxic signalling was less prominent within tumor regions but exhibited higher levels in fibrotic and the adjacent liver tissue (Fig. 3I).

In summary, our results demonstrate that specific characteristics of the CRLM TME are accurately captured on single-cell and spatial level after resection and prior to NMP, providing a robust foundation for subsequent evaluations.

### Impact of ex vivo perfusion on compositional integrity

**Fig. 4:**
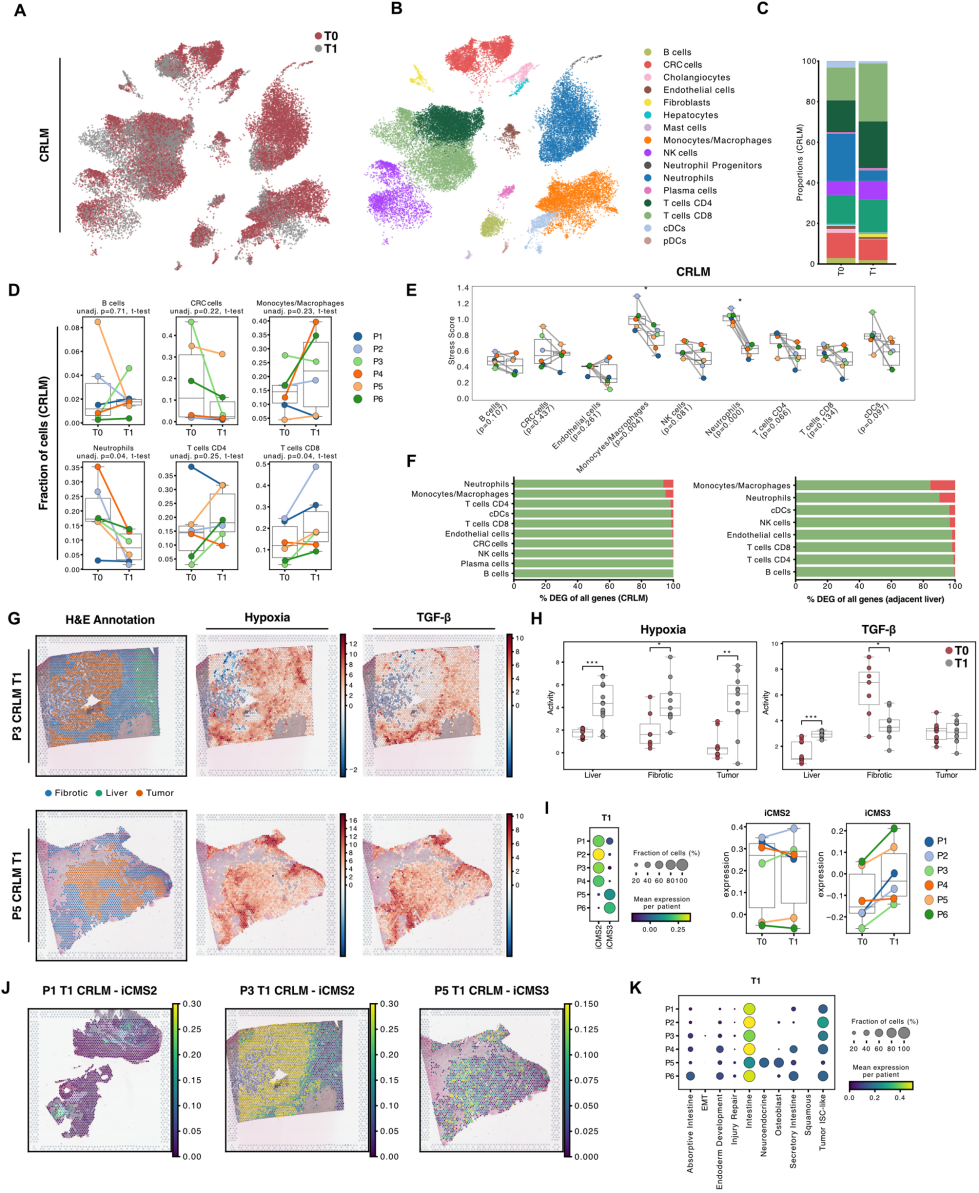
Impact of NMP on ex vivo CRLM (T0 vs. T1) **A** UMAP color-coded by time point pre NMP (T0) and after NMP (T1) or (**B**) cell-type of CRLM tissue. **C** Relative cell type composition in CRLM tissues at T0 and T1 NMP. **D** Cell-type proportions per patient in CRLM tissue. **E** Stress-scores in CRLM calculated as mean per cell-type and patient compared between T0 and T1. **F** Barplots showing the percentage of DEGs between time points (T0 vs. T1). DEGs were calculated between T0 and T1 of cell types in CRLM (left) or adjacent liver tissue (right). **G** Scatter plots showing H&E based annotation (left panel) and inferred pathway activities based on the ProgenY network per spot in CRLM tissue (right panel) at T1. **H** Comparison of mean pathway activities per ROI (divided into adjacent liver, fibrotic and tumor regions) between time points. **I** iCMS2 and iCMS3 mean scores of mCRC cells compared between T0 vs. T1 and visualized as dotplot (left panel) and boxplots (right panel). Each line indicates matched samples per time point and patient. **J** Spatial images of tumor tissue showing iCMS2 and iCMS3 expression per patient at T1. **K** Metastatic differentiation programs of mCRC cells at T1 represented as dotplot per patient

Given the accurate representation of mCRC phenotypes and TME characteristics at baseline, the stability of tumor and TME over time was assessed. We recently demonstrated that up to 24 h NMP of healthy transplanted livers induces a significant decrease in neutrophil cell numbers, while other cell types remain stable [28]. Thus, we first evaluated the impact of NMP on cell type composition in our model (Fig. 4A-B, Fig. S6A). In line with our previous report in donor livers, the proportion of neutrophils decreased significantly after a median of 41 h of NMP. This alteration was accompanied by a proportional increase of CD8^+^ T cells (Fig. 4C, Fig. S6B). Other cell types remained stable during NMP. These patterns were consistent in adjacent liver and CRLM tissues across all patients (Fig. 4D, Fig. S6C). Notably, the reduction in neutrophils was more distinct in adjacent liver tissue than in the tumor. We further accounted for the impact of compositional shifts by Bayesian Modelling (scCODA) [41]. The analysis confirmed proportional shifts in both tissues, verifying a more pronounced perfusion effect in adjacent liver tissue compared to CRLM tissue (Fig. S6D). Dynamic changes in leukocyte proportions were also visible in serial perfusate samples over an extended period of up to 168 h (Fig. S6E). Qualitative analysis of TME integrity indicated that NMP preserved the tissue architecture and immune cell presence (Fig. S7A). In line with transcriptomic data, we also identified macrophage-rich fibrotic regions located in close proximity to tumor borders.

Collectively, compositional analysis revealed that NMP reciprocally affects hepatic neutrophil and T cell proportions, while other cell types and TME integrity remained stable.

### Ex vivo perfusion preserves the gene expression profile of the CRLM TME

Next, we evaluated the impact of NMP on cellular transcriptomic profiles in adjacent liver and CRLM tissues. We initially evaluated whether ex vivo perfusion alters cellular stress levels by analyzing the abundance of mitochondrial reads as well as a score of stress- and apoptosis-related genes (Fig. S8A). Our results show that NMP did not induce cellular stress across cell types (Fig. 4E, Fig. S8B), which was also confirmed by spatial data (Fig. S8C-D). Notably, we identified an overall trend towards a reduction in cell-type specific stress in both adjacent liver and CRLM tissue. This is consistent with a recent finding in transplanted livers [28]. DEG analysis revealed that our ex vivo model largely preserves the gene expression profile across cell populations, both in adjacent liver and CRLM tissues (Fig. 4F). In line, the global and intra-patient composition of T- and NK cell subpopulations maintained stable throughout NMP (Fig. S9A-F) [42]. The only exception was a limited impact of NMP on the transcriptomic profile of monocytes/macrophages and neutrophils. We expanded our analysis to assess how NMP influences the spatial representation of functional processes. Similar to the findings in non-perfused livers, Hypoxia and TGF-β activated pathways were primarily localized in fibroblast-rich regions (Fig. 4G, Fig. S10A-B). ROIs showed comparable ProgenY activities across time points, with the most pronounced effect on all tissues observed in the Hypoxia and TNF-α pathway (Fig. 4H, Fig. S10C). We then assessed mCRC cell viability and the preservation of their metastatic phenotype in our ex vivo model. Selected genes associated with cell proliferation revealed no changes over perfusion time (Fig. S10D). Similarly, the patient-specific iCMS subtypes were consistently detectable until the end of NMP at both single-cell and spatial level (Figs. 3C and Fig. 4 I-J). We further identified mCRC cells to occupy similar metastatic programs as before perfusion (Fig. 4K).

Collectively, our findings provide evidence that ex vivo perfusion largely preserves the gene expression profile of tumor cells and relevant cell types within the CRLM TME, with only limited transcriptomic changes related to the myeloid compartment.

### CRLM tumor-associated myeloid cells persist during ex vivo perfusion

**Fig. 5:**
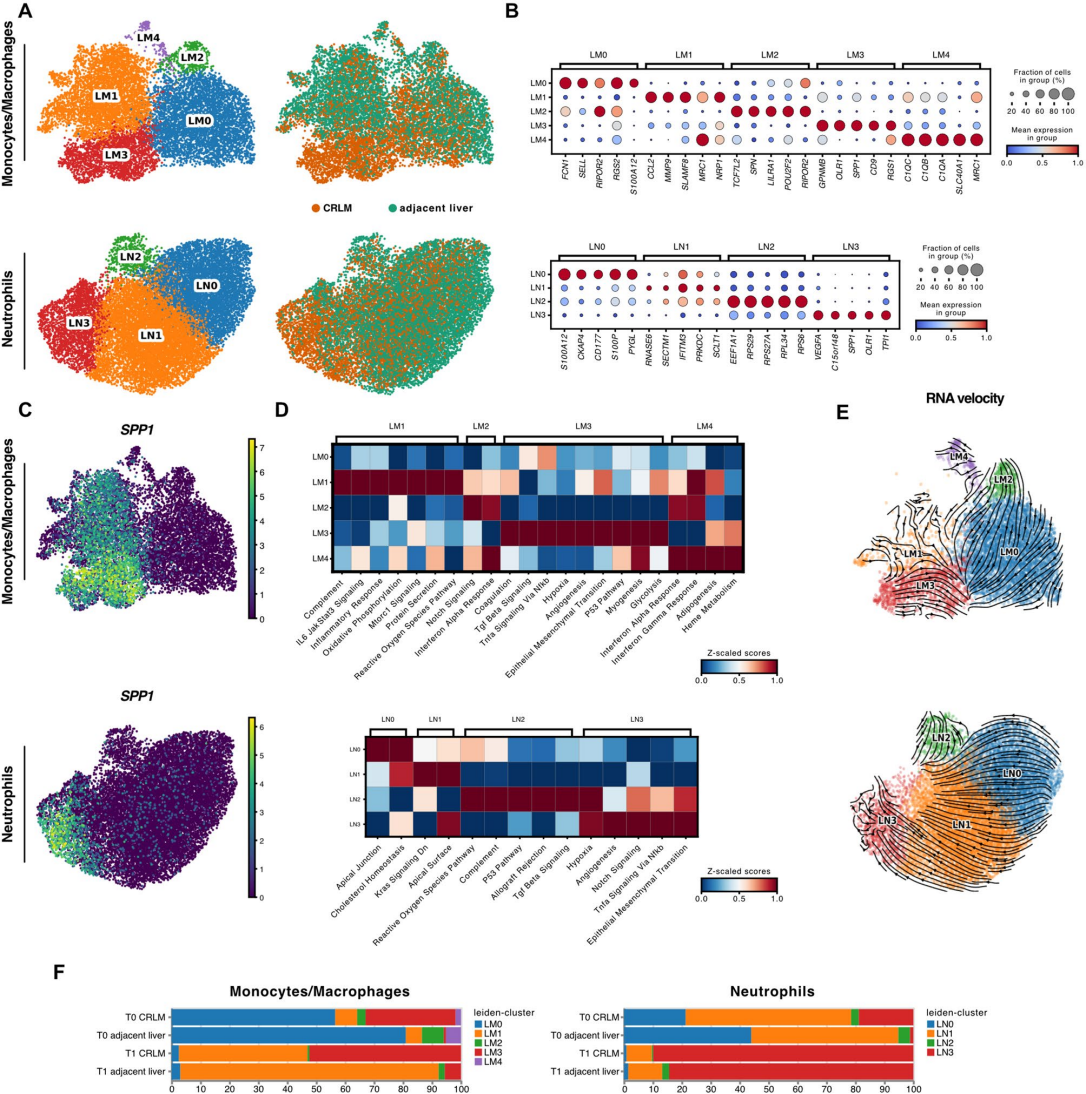
NMP persistence of tumor-associated myeloid cells **A** UMAP of monocytes/macrophages (top panel) and neutrophils (bottom panel) color-coded by leiden-clusters (left) or tissue-type (CRLM or adjacent liver - right). **B** Top five ranked genes per leiden-cluster for monocytes/macrophages (LM0-LM4) and neutrophils (LN0-LN3) without perfusion influence (T0). **C** SPP1 expression displayed on UMAPs of monocytes/macrophages (top) and neutrophils (bottom). **D** Enriched pathways calculated by GSEA ORA per leiden-cluster before perfusion start (T0). **E** UMAP of monocytes/macrophages (top) and neutrophils (bottom) with projected RNA velocities on top at T0. **F** Relative percentages of individual leiden-clusters divided by tissue-type (adjacent liver and CRLM) and time point (T0 vs. T1)

In a next step, we analysed phenotypic changes of myeloid cells, specifically monocytes/macrophages and neutrophils, in more detail. Compositional observations revealed a stronger effect of perfusion on adjacent liver than on CRLM in myeloid cells (Fig. S6C). Strikingly, changes in DEG revealed shared gene expression differences at T0 and T1 for both cell types (Fig. S11A). Leiden sub-clustering identified five distinct monocyte/macrophage clusters (LM0–LM4) and four neutrophil clusters (LN0–LN3), respectively (Fig. 5A). Phenotypic characterization at baseline (T0) revealed LM0 as pro-inflammatory (*FCN1*, *SELL*, *S100A12*), LM1 as activated (*CCL2*, *MMP9*), LM2 as anti-inflammatory, M2-like (*TCF7L2*,* SPN*,* LILRA1*), and LM4 as M2 phenotype associated with tissue repair functions (*C10C*,* C1OB*,* C1QA*) and further consistent with a phenotype previously identified in healthy ex vivo perfused livers (Fig. 5B – top) [28]. LM3 was characterized by high expression of *SPP1* and *OLR1*, indicative for a tumor-associated macrophage (TAM) phenotype [34]. The identified neutrophil subclusters LN0 (*S100A12*, *CD177*) and LN1 displayed activated, immune-responsive phenotypes (*IFITM3*, *RNASE6*, Fig. 5B – bottom). LN2 expressed ribosomal genes (*RPS29*, *RPL34*), resembling neutrophil clusters earlier identified in non-small cell lung cancer (NSCLC) and severe COVID-19 [43, 44]. Similarly to LM3, LN3 expressed high levels of *SPP1* and *OLR1* (Fig. 5C), markers associated with tumor-associated neutrophils [45, 46]. Notably, LM3 and LN3 were markedly enriched in CRLM tissue (Fig. S11B). ORA identified activated pathways in each subcluster that aligned well with their respective phenotypes (Fig. 5D). In particular, LM3 and LN3 exhibited upregulation of TGF-β or Hypoxia pathways, respectively, supporting their tumor-associated immunosuppressive phenotypes.

RNA velocity analysis indicated a terminal transition within neutrophils towards the LN3 phenotype (Fig. 5E – bottom). Consistently, LN3 shared features with a recently discovered terminal transition state that was functionally reprogrammed by the TME [47]. Interestingly, we identified a similar trajectory within monocytes/macrophages towards a shared state within LM3 (Fig. 5E – top).

We further assessed the impact of ex vivo perfusion on individual monocyte/macrophage and neutrophils subclusters (Fig. S11C) through comparison between time points of the same tissue origin (Fig. S11D). In CRLM tissue we observed a marked increase of LM1 over perfusion, but a decrease of LM0, LM2 and LM4. LM3 was the most stable subcluster in CRLM tissue during NMP (Fig. 5F – left, Fig. S11D – top). A comparable compositional shift of monocytes/macrophages was evident in adjacent tissue (Fig. 5F – left, Fig. S11D – top). Neutrophil subclusters were largely depleted in CRLM during NMP, with the exception of LN3 which persisted throughout perfusion (Fig. 5F – right, Fig. S11D – bottom). Notably, we also identified an increase of LN3 in adjacent tissue at the end of perfusion (Fig. S11D – bottom).

Together, our findings demonstrate that tissue-resident myeloid cells are primed towards shared tumor-associated states which persist throughout NMP.

### TME-driven persistence of immunosuppressive myeloid cells promotes disease progression and poor survival in CRC

**Fig. 6:**
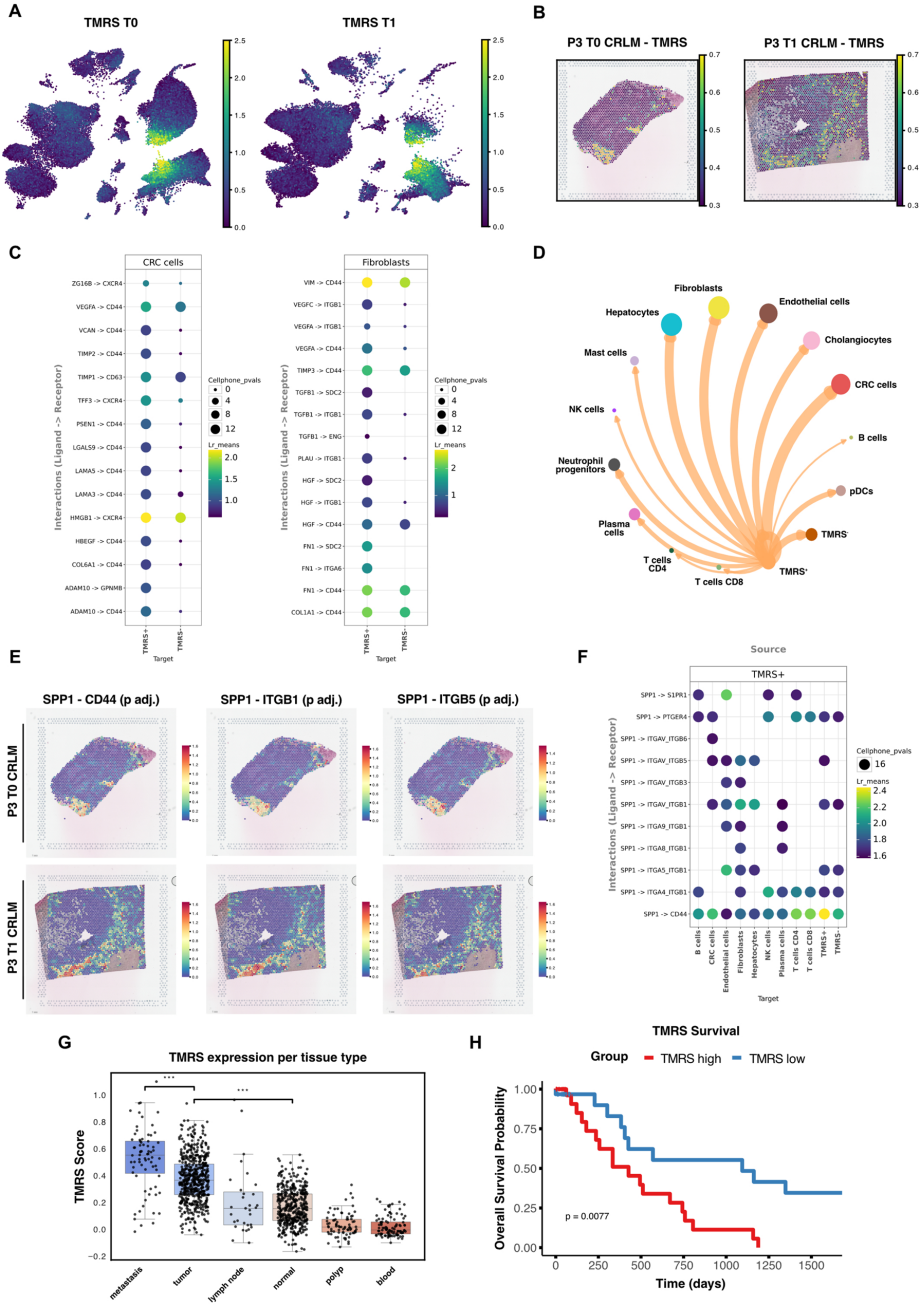
TME drives immunosuppressive myeloid cell persistence and poor survival **A** TMRS expression of single-cells in CRLM tissue at T0 (left) and T1 (right). **B** Representative image of patient 3 showing TMRS expression per spot in CRLM tissue at T0 and T1. **C** Top 15 ligand-receptor interactions predicted by LIANA of TMRS^+^ and TMRS^−^ myeloid cells with mCRC cells and fibroblasts. **D** Circular plot illustrating cellular communication between TMRS^+^ myeloid cells and other cell types. **E** Predicted interaction strength of *SPP1* with its targets by LIANA per spot before (T0 - top) and after (T1 - bottom) NMP in CRLM tissue of patient 3. **F** Predicted ligand-receptor interactions of *SPP1* in TMRS^+^ cells with other cell types at T0. **G** Mean TMRS expression per tissue and patient from a large-scale publicly available CRC atlas [32]. **H** Overall Survival Probability in a TCGA-COAD (*n* = 59) cohort divided into high or low expression of TMRS

Given that LM3 and LN3 occupy terminal differentiation states with high phenotypic similarity, we hypothesized that their persistence during NMP is driven by the same underlying process. Hence, we defined a tumor-associated myeloid cell resistance signature (TMRS) based on the gene intersection of the persistent myeloid cell clusters LM3 and LN3 (Table S5). Using this signature we classified myeloid cells as either TMRS^+^ or TMRS^-^ (Fig. 6A). Spatial analysis revealed that TMRS^+^ myeloid cells predominantly reside within fibrotic areas (Fig. 6B), matching with earlier identified hotspots of TGF-β and Hypoxia signalling before and at the end of NMP (Fig.4G). Given their enrichment within specific areas, we speculated that TMRS⁺ persistence was likely driven by TME-mediated local cues. Cell-to-cell communication analysis by ligand-receptor pair expression (LIANA, see *Methods*) revealed consistently stronger interactions from stromal and epithelial cells to TMRS^+^ cells compared to their TMRS^-^ counterparts (Fig. 6C). Notably, most incoming TMRS^+^ interactions targeted *CXCR4* and *CD44* signalling pathways. These pathways are known for their roles in recruiting immunosuppressive myeloid cells and interacting with extracellular matrix (ECM) components [48, 49]. These findings suggest that stromal cells actively steer the persistence of tumor-associated myeloid cells within the CRLM TME.

Outgoing communication from TMRS^+^ cells was predominantly driven by the *SPP1* axis. This was consistent with the findings in the corresponding ST dataset (Fig.6D and E). *SPP1* itself appeared to strongly interact with fibroblast-expressed integrin pairs and *CD44* on other cell types (Fig. 6F). *SPP1* is known, particularly in macrophages, to promote tumor immune border formation and thereby therapy resistance, which is associated with reduced survival [50]. Notably, *SPP1-CD44* signalling – typically promoting T cell inhibition/exhaustion [34] – was markedly elevated from TMRS^+^ cells towards T cells, indicating an immunosuppressive role of these tissue-retaining cells (Fig. 6F). Analysis of a large-scale human CRC single-cell atlas [32] revealed elevated TMRS signature expression in primary tumors and metastases (Fig. 6G), linking persistent tumor-associated myeloid cells to advanced disease and an immunosuppressive, aggressive TME. Consistently, patients with elevated TMRS expression exhibited significantly reduced overall survival in a large TCGA-COAD cohort (Fig. 6H; *n* = 59).

In summary, our ex vivo CRLM model uniquely captures intratumoral myeloid cell dynamics, offering novel insights into their persistence within a functional human TME. Our findings provide suggestive evidence, that TME-driven immunosuppressive myeloid cell persistence promotes disease progression and poor survival in CRC.

## Discussion

We apply an innovative model for cancer research using ex vivo NMP to study the full complexity of the CRLM TME. Long-term NMP of liver grafts has been proposed as a potent preclinical platform [19], and particularly for CRLM, there is a clear need for more accurate models with high clinical translatability. To address this, we established and thoroughly profiled a CRLM model by applying NMP to six human livers with viable mCRC lesions, utilizing single-cell and spatial mapping.

NMP, originally developed for clinical application [51], has previously been adapted for experimental perfusion of non-transplantable and diseased human livers [21, 24, 52, 53]. A proof-of-concept study demonstrated the feasibility of perfusing resected liver parts using a custom-built device [52], while a more recent study successfully maintained an explanted full-size liver with CRLM for multiple days using a modified commercial device [21]. We and others have reported perfusion of explanted full-size cirrhotic livers using commercially available NMP systems [19, 24]^,^ [53] and have previously investigated split liver graft perfusion in a clinical context [54].

For the first time, we applied NMP to both explanted full-size and partially resected livers using an unmodified, commercially available perfusion device, distinguishing it from studies that relied on custom-built or heavily modified systems. We demonstrate that partially resected and full-size explanted liver specimens carrying CRLM can be surgically reconstructed, perfused, and maintained viable on a closed-circuit NMP device stable for up to 144 h. All perfused liver specimens fulfilled established quality criteria – such as arterial and portal venous flow rates [55, 56] – and maintained bile production and architectural integrity of both tumor and adjacent liver tissue throughout perfusion.

Comprehensive multi-omics profiling confirmed mCRC phenotypic traits through metastatic programs and iCMS classification, while aberrant TGF-β and Hypoxia signalling highlighted functional TME representation beyond mere macroscopic integrity. Given the prognostic relevance of cancer cell-intrinsic phenotypes, their precise recreation in tumor models is essential [57]. Our results demonstrate that epithelial tumor phenotypes represented by iCMS class and differentiation state, previously defined in primary CRC [31], are also reliably represented in CRLM before and during NMP. This eliminates the reliance on pathway-level classifiers, which limit clinical translatability [58]. Notably, the presence of non-canonical states, typically associated with poor prognosis, highlights the preclinical potential of the proposed model system [33]. Furthermore, the coexistence of differentiation states within patients in our model confirms functional plasticity, mirroring a fundamental characteristic of mCRC. Tumors follow a stepwise progression from differentiated intestinal stem cell-like, to more fetal progenitor-like, ultimately resulting in late differentiated non-canonical states [33].

Evaluation of temporal integrity demonstrated that NMP largely preserves cell type composition and transcriptomic phenotypes with only limited impact on myeloid cells. Hypoxia and TNF-α signalling were increased post-perfusion across all tissue types, likely reflecting tissue remodeling and repair mechanisms [59–64], potentially triggered by necessary surgical adaptations before perfusion start. While ischemia-reperfusion injury (I/R) may contribute to hypoxic signalling, its impact appears minimal, as indicated by stable Wnt signalling levels [65].

Finally, our analysis demonstrated the model’s ability to capture intratumoral dynamics and revealed that the persistence of tumor-associated myeloid cells is primarily driven by local environmental cues. We provide evidence that tumor cells recruit and retain immunosuppressive myeloid cells via *CXCR4*, enhancing tissue residency and survival within the TME [49, 66, 67]. Stromal cells support myeloid cell persistence through *CD44*, which interacts with ECM components such as vimentin and collagen, reinforcing tissue retention [68, 69]. Additionally, tumor-associated myeloid cells sustain their own persistence by expressing *SPP1*, a key adhesion molecule that remodels the ECM through fibroblast interaction, creating hypoxic, fibrotic niches with increased stiffness and altered chemokine signalling [70, 71]. Accordingly, we identified *SPP1*⁺ myeloid cells predominantly in regions with restricted immune cell movement. This is consistent with tumor immune border formation and reinforcement of myeloid cell retention [34]. Our findings suggest a role of persistent tumor-associated myeloid cells in disease progression and poor survival in CRC, aligning with large-scale single-cell and TCGA data analysis that link their presence to advanced disease and poor survival.

Our cancer model has technology-inherent limitations. The use of a closed perfusion circuit, unlike open systems, requires meticulous surgical reconstruction of the inflow and outflow before NMP can be initiated. This process can be tedious requiring multiple hours of work on the back-table, thereby potentially affecting organ quality. Additionally, organ availability depends on local therapeutic practices for mCRC management, though recent evidence showing a survival benefit in transplanted patients with unresectable CRLM may increase access for preclinical studies [72]. While our findings confirm phenotypic stability during long-term perfusion, further research is needed to determine whether extended perfusion similarly preserves molecular integrity. Although the model effectively captures tumor-associated myeloid phenotypes, its applicability remains limited in fully characterizing the broader spectrum of myeloid cell populations in CRLM.

## Conclusions

Single-cell and spatial mapping confirmed the preservation of tumor phenotypes and their associated TME throughout perfusion. In-depth analysis revealed the persistence of long-lived, immunosuppressive myeloid cells, actively shaped by the TME. Also, our work highlights the potential of NMP as a preclinical model for tumor biology, bridging the gap between animal models and early-phase clinical trials. Thus, we introduce a unique framework that enables simultaneous ex vivo investigation of metastatic cell biology and intratumoral immune cell dynamics. This platform holds promise for future applications, including the preclinical testing of novel drugs or cellular therapies and improved preselection of patients for subsequent LT, paving the way for translational and clinical advancements.

## Methods

### Study design and patient inclusion

Adult patients with CRLM undergoing either resection or transplantation – in case of bilobar unresectable CRLM – were considered for inclusion in this study. Technical factors, such as the resection type and the projected configuration of the resected or explanted specimens, served as exclusion criteria. Further patient characteristics are summarized in Table S1. Between March 2021 and January 2024, a total of six patients were included.

### Surgical preparation for normothermic machine perfusion and sample collection

All anatomic resections were carried out by experienced hepatobiliary surgeons. Following LR, the hepatectomy specimens were put on ice in 4 °C cold perfusion solution and flushed with Custodiol^®^ HTK solution (Dr. Franz Köhler Chemie GmbH, Bensheim, Germany, + 5000 IE heparin) via the portal vein as well as the hepatic artery (warm ischemia time ≤ 4 min in all cases). Next, biopsies (T0) of the tumor and the adjacent liver tissue were taken. Tumor biopsies were taken ultrasound-guided to guarantee reliable tumor sampling (Fig. S1E) with the help of a coaxial needle. Biopsy tracts were occluded with fibrin glue to prevent bleeding as the coaxial needles were removed.

Once biopsies were taken, back-table preparation was commenced. For resected specimens the portal vein was lengthened in an end-to-end fashion using 5 − 0 Prolene™ sutures (Ethicon, Somerville, New Jersey, USA) and a Peri-Guard^®^ Pericardium Patch (Synovis Surgical Innovations, Saint Paul, Minnesota, USA) and cannulated. Second, the outflow was reconstructed in an end-to-side fashion using 4 − 0 Prolene™ sutures (Ethicon, Somerville, New Jersey, USA) and a Peri-Guard^®^ Pericardium Patch (Synovis Surgical Innovations, Saint Paul, Minnesota, USA, Fig. S1C). The sectoral or rather segmental bile ducts were cannulated directly using PVC pancreas stents which were connected via a Y-tube (Fig. S1E). In all cases there was no need to reconstruct the hepatic artery as the length and diameter of the vessel was sufficient for direct cannulation (Fig. S1E).

In three cases a full-size, explanted liver was obtained from patients undergoing LT for bilobar, unresectable CRLM. At our center a conventional, cava replacing approach is used for LT, thus all livers were explanted with their native inferior vena cava (IVC). At the suprahepatic IVC site, the liver veins were joined together using 5 − 0 Prolene™ sutures (Ethicon, Somerville, New Jersey, USA) to create a single orifice and a Peri-Guard^®^ Pericardium Patch (Synovis Surgical Innovations, Saint Paul, Minnesota, USA) was sutured to the newly created single orifice to close the suprahepatic IVC opening. Biopsies were taken in analogy to LR specimens as described above. The full-size livers were then cannulated in standard fashion in anticipation of NMP. Once the cannulas were in place the liver specimens were transferred to the OrganOx^®^ Metra^®^ and NMP was commenced (Fig. S1D-F). We aimed to achieve 36 h of normothermic ex vivo perfusion carefully extending the maximum clinical duration of 24 h. This duration was chosen to ensure that the final histopathological evaluation remained uncompromised. Contrary to other studies which used non-transplantable organs, we utilized resected and explanted livers from patients with CRLM which require histopathologic examination for accurate and adequate TNM staging. Perfusions were extended to approximately 40 h in five out of six cases for logistic reasons. In one case (#P3), following favorable outcomes after the first two perfusions, the liver specimen was kept on the perfusion device after the second biopsy sample was collected for a total of 64 h. In all cases final histopathologic workup was successfully performed and was not affected by prolonged NMP. The OrganOx^®^ Metra^®^ is a commercially available NMP device which has been CE marked and FDA approved. Compared to other commercially available devices, the OrganOx^®^ Metra^®^ constitutes a closed perfusion circuit which delivers blood, oxygen and nutrients to the liver at physiologic temperatures to mimic ideal physiologic conditions [73]. More specifically, the perfusate consisted of three units of type O leukocyte-depleted (30 Gy irradiation) packed red blood cells (PRBC, 280 ml each), 500 ml Gelofusine (B. Braun, Melsungen, Germany) and additives (cefuroxime, heparin, calcium gluconate, insulin, amino acids, epoprostenol and bile salts) as per the manufacturer’s protocol.

In addition to cefuroxime, a broad-spectrum antimicrobial agent (piperacillin/tazobactam) was added to the perfusion circuit prophylactically. Strict aseptic handling was applied throughout the experiments and perfusate cultures were taken at the end of all perfusions (negative in all cases). At the end of perfusion, following end-biopsies (T1), the hepatectomy specimens were placed in formaldehyde and transferred to the pathology department for final histopathologic workup.

### Pilot-experiment of NMP for 168 h of patient 7

Due to the success of extended perfusions up to 64 h (#P3), we piloted an additional tumoral liver (#P7) to demonstrate extended perfusion until parameters deteriorated (168 h). To not jeopardize the primary goal of uninterrupted perfusion, we limited analysis on selected immune cell dynamics of serially taken perfusate samples by routine hospital diagnostics as well as biopsies taken after NMP.

### Tissue preparation for scRNA-seq and flow-cytometry

Detailed instructions for preparation of viable single-cells for scRNA-seq and flow-cytometry from liver tissue can be found in our STAR protocols article [26]. Briefly, tumor and normal adjacent liver biopsies were taken pre (T0) and at the end of NMP (T1). Both tissues were minced into small pieces and incubated with the BD TuDoR dissociation reagent (BD Biosciences, Heidelberg, Germany) for 10 min at 37 °C while shaking. Afterwards, single-cells were filtered using a 100 μm cell strainer and subsequently red blood cells lysed with the BD Pharm Lyse (BD Biosciences, Heidelberg, Germany). Prior to scRNA-seq and flow-cytometry, cell viability was evaluated with Draq7 (BD Biosciences, Heidelberg, Germany) and Calcein-AM (Thermo Fisher Scientific, Massachusetts, USA).

### scRNA-seq library preparation and sequencing

Biopsies (CRLM and adjacent tissue) were immediately processed and prepared for scRNA-seq and flow-cytometry. Whole transcriptome libraries were generated following the BD Rhapsody WTA protocol with sample-tag labeling. We selected the microwell-based BD Rhapsody scRNA-seq platform to capture essential TME components. This system is particularly effective for capturing low-mRNA-content cells, such as neutrophils, though it may exclude larger cells (> 40 μm, such as hepatocytes). Following WTA and indexing, library quality was assessed using the 4200 TapeStation system and the Qubit dsDNA HS (High Sensitivity) assay kit. Sequencing was performed on a NovaSeq 6000 (Illumina, San Diego, California) using the S1 Reagent Kit v1.5 (200 cycles, 68 bp index read 1; Illumina, San Diego, California), achieving a sequencing depth of approximately 50,000 reads per cell.

### scRNA-seq data pre-processing, quality control and analysis

Raw FastQ files were processed using the Seven Bridges Genomics Platform with a fixed cell count of 150,000 to retain cell types with low mRNA content. Stringent quality control and AnnData containerization was performed using Scanpy [74]. Briefly, we performed filtering keeping only high quality cells with 200–8000 genes, 1000-100,000 transcripts and < 30% mitochondrial transcripts. Dataset integration was performed on raw counts using scVI on the 4000 most highly variable genes (*HVGs*, flavor = “seurat_v3”) with “sample” set as batch key. Based on batch corrected scVI embeddings, neighboring graphs and uniform manifold approximation and projections (UMAP) were computed. We performed cell type identification applying Leiden based clustering and annotated them based on characteristic canonic marker genes as well as top expressed genes (Table S2). Subcluster annotation was performed using a combined approach of Scanpy’s ranked gene function and literature based markers [34, 46, 47, 75, 76].

For differential gene testing, we pseudobulked samples based on biological replicates using the decoupler-py implementation [77]. Single-cell data were aggregated into pseudobulk profiles using the get_pseudobulk function from the decoupler package, requiring at least 10 cells per sample–cell_type combination (min_cells = 10) and a minimum of 1000 total counts for that combination (min_counts = 1000). After aggregation, genes were retained only if they had ≥ 10 total counts across all pseudobulked samples. This two-stage filtering approach excludes lowly abundant cell groups and lowly expressed genes, ensuring that downstream analyses focus on robust signal profiles. PyDESeq2 was performed comparing conditions (time point or tissue) including patient as additional covariate to account for inter-patient variability and repeated measures (design_factors=[“patient”, “condition”]). For inference of pathways and over representation analysis (ORA) we used foldchanges per cell type as input and the progenY and hallmark networks as implemented in the decoupler-py package. All scoring analyses were performed using the Scanpy “score_genes” function and the respective genes provided as list input. Previously published gene sets were used to calculate the iCMS signatures, cancer phenotypic states, and stress/apoptosis-related signature [28, 31, 33].

For subcluster analysis, we performed ORA on single-cells using logarithmized gene matrices as input. We used the python implementation of infercnv (https://github.com/icbi‑lab/infercnvpy) to infer copy-number variations from scRNA‑seq data. Additionally, we performed RNA velocity applying velocyto and scvelo [78]. We preprocessed BAM files with samtools and merged generated loom files into the main anndata object. RNA velocities were calculated using a dynamical model and visualized according to the scvelo tutorial. To systematically evaluate potential ligand–receptor interactions between cell populations, we employed the LIANA (v 1.5.0) framework in its “consensus” mode. Specifically, we focused on the CellPhoneDB method within LIANA by grouping cells based on their annotated cell types (groupby=’cell_type’) and referencing the “consensus” resource. To ensure robust detection, the expression proportion threshold was set to expr_prop = 0.1, and results were stored under key_added=’cpdb_res’. To generate interaction overviews, we first employed LIANA’s dotplot function to visualize ligand–receptor means and associated p-values by cell-type groups. We filtered results for significance (*p* ≤ 0.05) and highlighted sources of interest (e.g. TMRS⁺ cells). For a global interaction network, we then used LIANA’s circle_plot to depict cell-type nodes and communication edges, customizing node/edge size and label offsets.

### Signature validation and survival analysis

We validated the extension of iCMS classifications to metastatic settings by applying them to mCRC cells of a newly published CRC atlas dataset [32]. The dataset was subsetted to mCRC cells, reclustered using batch corrected scVI embeddings and finally iCMS scores were calculated as stated above. Similarly, we validated our identified myeloid signature by subsetting to neutrophil/myeloid cells and computing mean TAM signature scores per sample grouped by sample type.

For survival analysis, RNA-seq data for TCGA-COAD were retrieved via TCGAbiolinks (v2.34.1) and loaded into a SummarizedExperiment (v1.36.0) object. Clinical metadata were merged to define a binary survival outcome (alive vs. deceased) and time to event (days to death or last follow-up), with patients lacking consistent survival data excluded. Raw count data were filtered to exclude low-abundance genes, followed by a variance-stabilizing transformation (VST) using DESeq2 (v1.46.0). The 12-gene signature and the corresponding transcripts were extracted from the processed dataset. The per-sample mean signature expression was computed, and patients were stratified into high vs. low signature groups. Kaplan–Meier survival curves were then generated and visualized using survival (v3.8.3) and survminer (v0.5.0) packages to assess the prognostic relevance of the signature. Data manipulation and visualization were performed using dplyr (v1.1.4), tidyr (v1.3.1), and ggplot2 (v3.5.1).

### Flow-cytometry

Isolated single-cells from biopsies of both tissues at T0 and T1 were stained with 19 antibodies (Table S6) and after washing and 7-AAD addition measured on the FACSymphony A5 flow-cytometer. We analysed generated data using the FlowJo v10.10 Software. For the exact gating strategy we refer to our recently published article [28].

### Histology and multiplex immunofluorescence staining

Normal adjacent and CRLM liver tissue samples were fixed in 4% formalin, embedded in paraffin and sectioned at a thickness of 3 μm for staining. H&E stained slides (Sakura Tissue-Tek) were scanned using either the VENTANA^®^ DP 200 slide scanner (Ventana Roche) with combination with the navify^®^ Digital Pathology platform or the Olympus VS120 Slide Scanner (Olympus). Digital slides were further processed with the QuPath software (v0.5.1) and ImageJ (v1.54 g) [79].

Multiplex IF staining on formalin-fixed paraffin-embedded (FFPE) tissue was conducted using the Opal 6-Plex Detection Kit (Cat. No: NEL821001KT, Akoya Biosciences, Menlo Park, USA). A panel of immune markers for tissue architecture (Vimentin, Cytokeratin) or immune infiltration (CD20, CD8, CD3, CD68, Cytokeratin) was applied using the automated BOND RX staining system (Leica Biosystems). Antibodies were applied sequentially and paired with their respective Opal fluorophores (antibody details are provided in Table S7). Cell nuclei were counterstained with spectral DAPI (Akoya Biosciences). Stained slides were scanned using the Mantra 2**™** Quantitative Pathology Workstation (Akoya Biosciences), and representative images were acquired with Mantra Snap software (v1.0.4). Spectral unmixing and multispectral image acquisition were performed using the inForm Tissue Analysis Software (v2.4.10, Akoya Biosciences).

### Spatial transcriptomics data pre-processing, quality control and analysis

For ST validation, three patients (patients 1, 3 and 5) were chosen based on tissue availability and diverging iCMS classifications. We employed the 10x Genomics Visium Spatial Transcriptomics platform to perform spatial gene expression profiling on FFPE tissue samples, following manufacturer-provided protocols. RNA quality was ensured by isolation with the Qiagen RNeasy FFPE Kit (Cat. No. 73504), followed by quality assessment using the Agilent Bioanalyzer RNA Pico Kit, confirming DV200 values exceeding 50%. FFPE tissue blocks were sectioned at 5 μm thickness and processed for deparaffinization, H&E staining, and library preparation, adhering to the Visium Spatial Tissue Prep Guide (CG000408) and Visium Library Preparation protocol (CG000407). The resulting libraries were sequenced on an Illumina NovaSeq 6000 platform (PE150 mode) at the NGS Core Facility in Vienna.

Raw sequencing data underwent quality control and alignment to the reference genome using Space Ranger (version 3.0.0). The generated gene-spot expression matrix was analysed using Python 3.9 and the Scanpy ecosystem (v1.10.3). To ensure data quality, spots with fewer than 500 detected genes, total counts below 1,000, or mitochondrial content exceeding 30% were removed. Gene expression values were normalized using Scanpy’s total-count normalization, where counts were scaled to the median total counts across all spots prior to normalization, followed by log1p transformation.

Marker gene signatures were computed using Scanpy’s tl.score_genes function for visualization the pl.spaital function was used. Deconvolution of ST data was performed with Cell2Location (v0.1.4), using our single-cell dataset as reference training data for the model. Spatial functional analysis, including transcription factor activity inference, pathway activity estimation, and functional enrichment of biological terms, was conducted using DecoupleR (v1.6.0). Histological annotations of ROIs were provided by an expert pathologist and integrated into the dataset using the Napari Spatial plugin. We defined five distinct ROIs per tissue sample when applicable with average pathway activities used for quantification. Spatial Cell2Cell Communication was analysed with stLearn(v 0.4.12).

### High-resolution respirometry

HRR was employed to evaluate mitochondrial function in normal adjacent liver and tumor biopsies as previously described [17]. Briefly, biopsies were homogenized in MiR05 respiration medium (MiR05-Kit, Oroboros Instruments, Innsbruck, Austria) to prepare a crude liver homogenate (final concentration of 1 mg/mL wet mass tissue). This was added into the 0.5 mL chambers of the HRR device (O2k, Oroboros Instruments, Innsbruck, Austria) and measurements were performed in technical duplicates at 37 °C under constant stirring at 750 rpm. Respiration rates were recorded using DatLab software (Datlab 7.4, Oroboros Instruments, Innsbruck, Austria) and expressed as O_2_ flux per wet mass tissue [pmol O_2_·s^−1^·mg^−1^]. A pre-defined Substrate-Uncoupler-Inhibitor-Titration (SUIT) protocol was applied to assess succinate-linked OXPHOS capacity. *P-L* control efficiency (j_*P−L*_) was calculated as a measure of ATP production efficiency and cytochrome *c* control efficiency (j_*cyt c*_) was calculated to assess the outer mitochondrial membrane integrity.

### Statistical analysis

To compare compositional data, we applied Bayesian modelling with scCODA [41] as implemented in pertpy (Version 0.9.4) We used the Models automatic reference cell type selection feature (Endothelial cells) to estimate abundance changes showing credible effects at an FDR of 0.1. Differential gene expression between conditions (time point or tissue) was performed using PyDESeq2 (Version 0.4.12) and single-cell data aggregated by biological replicate. Statistical testing for ST and scRNA-seq paired and unpaired results was performed using Python (statsmodels library) adjusting for multiple hypotheses when required (Benjamin-Hochberg). Statistical testing for other data (flow-cytometry and IF) was performed using Graphpad Prism with either linear modeling, t-testing or wilcoxon test as appropriate.

## Supplementary Information

Supplementary Material 1.

Supplementary Material 2.

## Data Availability

The datasets supporting the conclusions of this article are available as download in h5ad format on Zenodo (10.5281/zenodo.15234826). We provide processed and annotated sequencing data of scRNA-seq and ST analysis, respectively. Additionally, we provide both the single-cell and spatial datasets via cell-x-gene, a web-based viewer allowing interactive visualization of both gene expression and metadata (https://cellxgene.cziscience.com/e/e3ed2ba4-edf5-40ac-8750-8a417ad1eefe.cxg/). Further available data can be found in supplementary files or shared upon reasonable request to the corresponding author.

## Abbreviations

CNV: copy number variation
CRC: colorectal cancer
CRLM: colorectal cancer liver metastasis
DEG: differential gene expression
ECM: extracellular matrix
FFPE: formalin-fixed paraffin-embedded
H&E: hematoxylin and eosin
HRR: high-resolution respirometry
iCMS: intrinsic-consensus molecular subtypes
IF: immunofluorescence
LT: liver transplantation
LR: liver resection
mCRC: metastatic colorectal cancer
MSS: microsatellite stability
NMP: normothermic machine perfusion
ROI: region of interest
scRNA-seq: single-cell RNA sequencing
ST: spatial transcriptomics
TCGA-COAD: Cancer Genome Atlas Colon Adenocarcinoma
TME: tumor- microenvironment
TMRS: tumor-associated myeloid cell resistance signature
TRG: Tumor Regression Grade
ORA: over-representation analysis
