# Supplementary material for: Ex vivo modelling of human colorectal cancer liver metastasis by normothermic machine perfusion
Source: Mol Cancer. 2025 Oct 21;24:264. doi: 10.1186/s12943-025-02430-7 (PMC12542284; doi:10.1186/s12943-025-02430-7)
Supplement: Supplementary file 1 — Supplementary Material 1. [file 12943_2025_2430_MOESM1_ESM.pdf]

Table S1. Patient characteristics CRC NMP (OrganOx® metra®).

							long-term perfusion
**Case**	**#1**	**#2**	**#3**	**#4**	**#5**	**#6**	**#7**
**Patient age (at diagnosis of the primary tumor)**	39	70	39	53	56	46	62
**Patient sex**	female	female	male	male	female	male	male
**Location of the primary tumor**	left	left	left	left	left	left	left
**Histology**	adenocarcinoma	adenocarcinoma	adenocarcinoma	adenocarcinoma	adenocarcinoma	adenocarcinoma	adenocarcinoma
**Synchronous CRLM**	yes	yes	yes	yes	yes	yes	yes
**Surgical treatment of primary tumor**	left hemicolectomy	left hemicolectomy	rectal resection	left hemicolectomy	none	rectal resection	extended left hemicolectomy
**Neoadjuvant therapy**	yes	no	yes	yes	yes	yes	no
**Adjuvant therapy**	no	yes	yes	yes	no	yes	yes
**Type of systemic therapy**	FOLFOX + Panitumumab	FOLFOX + Panitumumab	CapOx + Panitumumab, FOLFIRI + Bevacizumab	FOLFOX6 + Panitumumab, FOLFIRI + Bevacizumab, FOLFOX6+ Panitumumab	FOLFOX + Bevacizumab	FOLFOXIR + Bevacizumab, mFOLFIRI, FOLFOX 6 + Bevacizumab	FOLFOX + Panitumumab
**Liver first vs. primary first**	primary first	primary first	primary first	primary first	liver first	primary first	primary first
**Pathology of the primary tumor (TNM)**	ypT2N0M1a(HEP), R0, G2,	pT3N1bM1a(HEP), R0, G2	ypT3N2M1a(HEP), R0	ypT3N0M1a(HEP), G2, R0	ycT0NxM1a(HEP), G2	ypT3N0M1a(HEP), R0	pT3N1M1a(HEP); R0; G2
**Microsatellite status**	MSS	MSS	MSS	MSS	MSS	MSS	MSS
**BRAF status**	BRAF^{wt}	BRAF^{wt}	BRAF^{wt}	BRAF^{wt}	BRAF^{wt}	BRAF^{wt}	BRAF^{wt}
**KRAS status**	KRAS^{wt}	KRAS^{wt}	KRAS^{wt}	KRAS^{wt}	KRAS^{wt}	KRAS^{mut}	KRAS^{wt}
**Time from last systemic treatment to LR/LT**	1 month	2 months	CTx until LT	CTx until LT	1 month	2 months	1 month
**Radiology findings (liver)**							
- Disease status before LR/LT	PR	PR	PD	PR	PR	SD	PR
**Treatment of CRLM**	LR	LR	LT	LT	LR	LT	LR
**Time from diagnosis of CRLM to LR/LT**	7 months	5 months	18 months	21 months	7 months	83 months	5 months
**LOS after LR/LT (days)**	7	11	12	12	9	15	8
**Complication**	no	no	yes	no	yes	yes	no
**Clavien-Dindo**	0	0	IIIa	0	I	IIIa	0
**Pathology findings (liver)**							
- Number of lesions	4	2	10	5	3	5	1
- Size of largest lesion (cm)	1.5	7	10	3.5	4.3	6.8	4.5
- Pathologic response	TRG 3[§]	TRG 3[§]	TRG 4[§]	TRG 3[§]	TRG 4[§]	TRG 2[§]	TRG 2[§]
- Tumor viability (%)	40%	10%	60%	30%	60%	5%	2%
**Status (Dead/Alive)**	Alive	Alive	Dead	Alive	Alive	Alive	Alive
**DFS (months)**	12	12	3	1	3	8	16
**OS (months)**	26	42	11	31	15	13	41
**NMP duration (h)**	40.1	42.3	39.5	64	41.5	38	168

DFS, disease free survival; LR, liver resection; LT liver transplantation; LOS, length of stay; OS, overall survival; PD, progressive disease. PR, partial response; SD, stable disease;

[§]according to Rubbia-Brandt et al.

Tumor Regression Grade (TRG) according to Rubbia-Brandt et al.

→ TRG 1: absence of tumor cells, replaced by abundant fibrosis

→ TRG 2: rare residual tumor cells scattered throughout abundant fibrosis

→ TRG 3: more residual tumor cells throughout a predominant fibrosis

→ TRG 4: large amount of tumor cells predominating over fibrosis

→ TRG 5: tumor cells without fibrosis

(PMID: 17060484)

Table S2. Top ranked genes per cell type cluster

group	names	scores	logfoldchanges	rank
B cells	MS4A1	61,44895	9,140854	1
B cells	CD79A	59,743603	9,237503	2
B cells	CD74	57,136925	4,4405217	3
B cells	HLA-DRA	51,939495	4,7138653	4
B cells	IGHM	50,998993	7,843442	5
B cells	BANK1	50,12476	6,909521	6
B cells	HLA-DQA1	48,30926	4,3584304	7
B cells	HLA-DPA1	46,454365	3,5053997	8
B cells	HLA-DRB1	45,66437	3,5121953	9
B cells	CD52	44,856762	3,0999503	10
B cells	HLA-DPB1	44,238785	3,3721533	11
B cells	FCMR	43,8956	3,1389232	12
B cells	RPS23	43,888718	1,9923064	13
B cells	RPS8	43,82463	1,9334853	14
B cells	RPL34	41,0213	1,7279267	15
B cells	BIRC3	40,317238	3,2863328	16
B cells	IGKC	40,020256	4,6900496	17
B cells	CD22	39,988354	7,248473	18
B cells	RPL11	39,925213	1,7297091	19
B cells	RALGPS2	38,909058	4,487902	20
B cells	TNFRSF13C	38,284416	7,051822	21
B cells	CD37	37,891495	2,7523558	22
B cells	MEF2C	37,721222	3,9198678	23
B cells	RPL39	37,254105	1,5214223	24
B cells	RPS27	37,040894	1,7076856	25
B cells	MT-CO2	36,67349	0,9317926	26
B cells	RPL13A	36,600338	1,723984	27
B cells	RPL32	36,46499	1,6292325	28
B cells	RPS6	36,09721	1,5986129	29
B cells	RPS13	36,067947	1,5964485	30
B cells	RPL9	36,06325	1,5560745	31
B cells	RPL21	35,807835	1,556963	32
B cells	HLA-DQB1	35,489628	3,1649702	33
B cells	NIBAN3	35,395683	7,442318	34
B cells	MT-ND4	35,37896	0,8860994	35
B cells	PAX5	35,25714	6,5075235	36
B cells	MT-ND3	35,11814	0,9486611	37
B cells	RPS5	34,858456	1,8147042	38
B cells	SWAP70	34,40051	3,3328705	39
B cells	MT-CYB	33,80615	0,91378236	40
B cells	HVCN1	33,515507	4,189761	41
B cells	RPS3A	33,452145	1,5157223	42

**B cells**	RPS12	33,377457	1,6498808	43
**B cells**	RPL23	33,33059	1,3956616	44
**B cells**	IGHD	33,14339	8,602288	45
**B cells**	RPS11	33,104347	1,6097744	46
**B cells**	HLA-DMB	33,080063	3,275326	47
**B cells**	MT-ND4L	32,70994	1,1693294	48
**B cells**	RPS27A	32,67402	1,492863	49
**B cells**	RPL12	32,396545	1,5471728	50
**B cells**	FCRL1	32,371952	7,3523574	51
**B cells**	MT-CO3	31,844608	0,8474205	52
**B cells**	RPS20	31,842339	1,4531301	53
**B cells**	IRF8	31,384632	2,8740718	54
**B cells**	ST6GAL1	31,363298	2,7680378	55
**B cells**	NAP1L1	31,236683	1,5729402	56
**B cells**	RPS29	31,194775	1,4435868	57
**B cells**	RPL31	31,18957	1,4018517	58
**B cells**	RPL10A	31,1514	1,5263499	59
**B cells**	EEF1A1	31,05765	1,4286721	60
**B cells**	RPL27A	30,958965	1,3906857	61
**B cells**	RPL5	30,792538	1,4570199	62
**B cells**	POU2F2	30,561224	3,0713427	63
**B cells**	RPL30	30,374063	1,31748	64
**B cells**	RPL37A	30,335892	1,402601	65
**B cells**	BCL11A	30,29374	5,087877	66
**B cells**	RPS21	30,157938	1,4715481	67
**B cells**	MT-ATP6	30,108763	0,8637193	68
**B cells**	RPL23A	29,968496	1,43853	69
**B cells**	RPL37	29,952114	1,2973741	70
**B cells**	MT-CO1	29,913218	1,0986367	71
**B cells**	SMIM14	29,76648	2,829338	72
**B cells**	MTATP6P1	29,681376	1,0986243	73
**B cells**	POU2AF1	29,654205	6,0523067	74
**B cells**	RPL13	29,646261	1,448817	75
**B cells**	AFF3	29,420269	4,5314183	76
**B cells**	RPL3	28,755524	1,39273	77
**B cells**	RPS25	28,317425	1,3101724	78
**B cells**	FCRLA	28,315731	8,85861	79
**B cells**	RPL38	28,212313	1,281501	80
**B cells**	RPS4X	28,115667	1,3720539	81
**B cells**	RPL27	27,825575	1,2825722	82
**B cells**	TCF4	27,655233	3,3601148	83
**B cells**	CD83	27,638079	2,9478223	84
**B cells**	MARCHF1	27,360886	3,0286095	85
**B cells**	RPL35A	27,334461	1,2746462	86

**B cells**	RPLP2	27,265669	1,4000857	87
**B cells**	RPL4	26,977133	1,3434478	88
**B cells**	RPS16	26,827822	1,199376	89
**B cells**	RPL19	26,497423	1,3355099	90
**B cells**	MT-ND1	26,112902	0,66050416	91
**B cells**	PARP15	26,106625	3,188364	92
**B cells**	HLA-DMA	26,035042	2,4859536	93
**B cells**	RPS2	25,975695	1,3058467	94
**B cells**	EEF1B2	25,781979	1,4349374	95
**B cells**	EZR	25,766804	1,7350422	96
**B cells**	CXCR4	25,705189	1,640291	97
**B cells**	RPSA	25,689812	1,4096355	98
**B cells**	CD19	25,667582	8,4547615	99
**B cells**	RCSD1	25,191765	1,7300907	100
**cDCs**	CD74	59,485203	5,6994543	1
**cDCs**	HLA-DPA1	59,337646	6,371725	2
**cDCs**	HLA-DRA	59,194702	6,9487386	3
**cDCs**	HLA-DPB1	58,705048	5,9808607	4
**cDCs**	HLA-DQA1	58,52407	6,628337	5
**cDCs**	HLA-DRB1	58,204693	5,9839635	6
**cDCs**	HLA-DQB1	57,6636	5,8131766	7
**cDCs**	HLA-DMB	51,47655	4,627331	8
**cDCs**	HLA-DMA	51,377254	4,192086	9
**cDCs**	HLA-DRB6	50,708973	5,0983963	10
**cDCs**	CST3	48,169445	4,255472	11
**cDCs**	HLA-DOA	46,418255	5,249589	12
**cDCs**	CD86	46,131817	4,1540475	13
**cDCs**	HLA-DRB5	43,256504	4,4217787	14
**cDCs**	CCDC88A	43,140106	3,3484316	15
**cDCs**	FGL2	41,577515	3,6037006	16
**cDCs**	TMSB10	40,92962	2,4519556	17
**cDCs**	SAMHD1	40,521217	2,6352751	18
**cDCs**	CPVL	40,229908	4,026187	19
**cDCs**	RNASE6	39,276264	3,5017965	20
**cDCs**	RGS10	38,995544	2,982077	21
**cDCs**	FGD2	38,34068	3,5483181	22
**cDCs**	MARCHF1	38,26061	3,3710558	23
**cDCs**	CTSH	38,16215	2,9721243	24
**cDCs**	KCTD12	38,098476	3,0206387	25
**cDCs**	DPYSL2	37,93641	2,9977407	26
**cDCs**	CIITA	37,90302	4,5866814	27
**cDCs**	IL13RA1	37,832695	2,9398198	28
**cDCs**	CD4	37,736618	2,7551818	29
**cDCs**	HLA-DQA2	37,658978	5,643619	30

**cDCs**	CLIC2	37,64423	4,9948444	31
**cDCs**	LYZ	37,555485	3,387052	32
**cDCs**	GSN	37,345757	2,9134648	33
**cDCs**	SPINT2	37,220165	2,7411942	34
**cDCs**	ALDH2	36,98835	3,3599112	35
**cDCs**	PEA15	36,166534	2,712031	36
**cDCs**	CDK2AP1	35,96331	3,0485034	37
**cDCs**	GRN	35,935425	2,3880057	38
**cDCs**	GPR183	35,782574	3,0540028	39
**cDCs**	VIM	35,41081	2,2056553	40
**cDCs**	MS4A6A	35,127453	3,401716	41
**cDCs**	AHR	35,020744	2,8045065	42
**cDCs**	PAK1	34,992317	2,5860744	43
**cDCs**	C1orf162	34,226234	2,6421428	44
**cDCs**	HERPUD1	34,034504	2,3630424	45
**cDCs**	MEF2C	33,558582	2,9101129	46
**cDCs**	RPS24	33,547436	1,5532535	47
**cDCs**	NAP1L1	33,43103	1,8479084	48
**cDCs**	FLT3	33,240788	5,679596	49
**cDCs**	RAB31	32,998222	2,2395408	50
**cDCs**	SHTN1	32,97575	2,9840655	51
**cDCs**	PPA1	32,916534	2,4490144	52
**cDCs**	LGALS9	32,84176	2,581112	53
**cDCs**	MACROH2A1	32,70199	1,9161248	54
**cDCs**	CD83	32,5721	3,0006845	55
**cDCs**	CD1C	32,55533	6,5839033	56
**cDCs**	GRK3	32,405518	2,9592576	57
**cDCs**	AIF1	32,363045	2,345513	58
**cDCs**	PLXDC2	32,293312	2,560939	59
**cDCs**	ALCAM	32,250732	2,7715955	60
**cDCs**	NPC2	32,178482	2,1646826	61
**cDCs**	GDI2	32,167595	1,8835636	62
**cDCs**	PPT1	32,067917	2,3478467	63
**cDCs**	TUBA1B	31,959793	2,0425453	64
**cDCs**	ZNF385A	31,671074	3,1768966	65
**cDCs**	SLAMF8	31,384308	3,219345	66
**cDCs**	CSF2RA	31,254427	3,275733	67
**cDCs**	NDRG2	31,166742	4,628356	68
**cDCs**	AP1S2	31,12609	2,125991	69
**cDCs**	MFSD1	31,117464	2,1296299	70
**cDCs**	PABPC4	31,116405	2,052967	71
**cDCs**	FILIP1L	31,034752	4,3067126	72
**cDCs**	COTL1	30,90723	1,9537659	73
**cDCs**	CTSZ	30,770645	2,1765645	74

**cDCs**	IL18	30,408953	3,2241435	75
**cDCs**	ADA2	30,345028	2,222432	76
**cDCs**	VAMP8	30,223946	1,8111495	77
**cDCs**	CXCL16	30,092236	2,0644336	78
**cDCs**	FCGR2B	29,942118	3,5175588	79
**cDCs**	CD302	29,898634	2,6776752	80
**cDCs**	GNB4	29,864134	2,6980512	81
**cDCs**	RGS1	29,680725	2,434396	82
**cDCs**	OGFRL1	29,592947	2,033256	83
**cDCs**	CSF1R	29,453194	2,8962002	84
**cDCs**	TSPAN33	29,387066	4,1952186	85
**cDCs**	METTL7A	29,301035	2,5847287	86
**cDCs**	LGALS1	29,300823	2,3258252	87
**cDCs**	CNDP2	29,233627	2,096208	88
**cDCs**	SLC25A6	29,21769	1,816011	89
**cDCs**	ANXA5	29,125324	1,8508477	90
**cDCs**	CTSB	28,95595	1,8210217	91
**cDCs**	YBX1	28,837893	1,5766528	92
**cDCs**	RPL23	28,804836	1,337844	93
**cDCs**	GSTP1	28,7632	1,983833	94
**cDCs**	DSE	28,607738	2,1644716	95
**cDCs**	RAC1	28,282274	1,5817057	96
**cDCs**	RPS8	28,282045	1,4526377	97
**cDCs**	MRC1	28,11259	3,3353086	98
**cDCs**	LAP3	28,005106	2,3124733	99
**cDCs**	GPX1P1	27,774801	3,2989824	100
**CRC cells**	EPCAM	104,77165	6,9369907	1
**CRC cells**	MAL2	99,18608	6,423797	2
**CRC cells**	CLDN4	98,04068	6,3222456	3
**CRC cells**	SPINT2	97,99437	4,6413784	4
**CRC cells**	DSG2	97,90476	6,3342395	5
**CRC cells**	DSP	97,576065	6,331593	6
**CRC cells**	KRT8	96,02619	6,1520042	7
**CRC cells**	CAMK2N1	95,513664	4,9689927	8
**CRC cells**	ELF3	94,78102	5,677969	9
**CRC cells**	CLDN7	94,69846	6,178866	10
**CRC cells**	KLF5	94,559975	5,7274804	11
**CRC cells**	DSTN	92,83522	3,9452317	12
**CRC cells**	CDH17	91,83741	6,4774756	13
**CRC cells**	JUP	91,434784	5,4127975	14
**CRC cells**	PERP	91,296364	4,530525	15
**CRC cells**	MAP7	91,29357	5,0807176	16
**CRC cells**	LGALS4	91,01846	5,7868204	17
**CRC cells**	ESRP1	90,24993	5,71943	18

**CRC cells**	H1-0	89,52305	4,6714463	19
**CRC cells**	NET1	89,305984	4,8652062	20
**CRC cells**	ATP1B1	89,02568	4,365739	21
**CRC cells**	TPD52	88,79183	4,042928	22
**CRC cells**	PLS1	87,93012	5,207589	23
**CRC cells**	HSP90AB1	87,59714	2,6692662	24
**CRC cells**	MUC13	87,43043	6,0591764	25
**CRC cells**	CDH1	87,20184	3,9590402	26
**CRC cells**	EMP2	87,137375	5,5212493	27
**CRC cells**	AGR2	87,12411	6,3316917	28
**CRC cells**	KRT18	86,70879	5,976481	29
**CRC cells**	MARCKSL1	86,69449	3,9068367	30
**CRC cells**	CD24	86,680595	4,8559914	31
**CRC cells**	APP	86,073875	3,4320369	32
**CRC cells**	CHMP4C	85,90437	6,397222	33
**CRC cells**	DDR1	85,71722	4,9915504	34
**CRC cells**	TPM1	85,1836	4,1449766	35
**CRC cells**	FNBP1L	85,1591	4,8677607	36
**CRC cells**	CD9	85,14605	4,06295	37
**CRC cells**	PLOD2	84,60047	5,3030343	38
**CRC cells**	KRT19	84,51804	5,8066225	39
**CRC cells**	PTPRF	84,51279	5,163364	40
**CRC cells**	PDLIM1	84,16341	3,838351	41
**CRC cells**	PTK2	84,08677	3,7182863	42
**CRC cells**	VIL1	84,025665	5,8790083	43
**CRC cells**	LMO7	83,18721	4,451916	44
**CRC cells**	DDAH1	83,07331	5,238773	45
**CRC cells**	HSPB1	82,58089	3,9974124	46
**CRC cells**	ERBB3	82,376015	5,2377577	47
**CRC cells**	PAWR	82,2417	3,998634	48
**CRC cells**	TSC22D1	82,229195	3,7389946	49
**CRC cells**	MDK	81,93892	6,1924095	50
**CRC cells**	PRXL2A	81,91454	4,513122	51
**CRC cells**	ARHGAP5	81,88499	3,3901474	52
**CRC cells**	NDFIP2	81,666756	3,4072878	53
**CRC cells**	MYO6	81,62658	3,9985077	54
**CRC cells**	CXADR	81,29019	5,2200546	55
**CRC cells**	GNG12	81,08958	4,4817085	56
**CRC cells**	COX6C	81,02253	2,682883	57
**CRC cells**	PLCB4	80,71882	4,787642	58
**CRC cells**	RPL7	80,55763	2,5342913	59
**CRC cells**	GFPT1	80,39965	3,254691	60
**CRC cells**	ICA1	80,35156	4,195545	61
**CRC cells**	ATP9A	80,267365	4,143362	62

**CRC cells**	PRAC1	79,839066	8,097014	63
**CRC cells**	EIF3E	79,638374	2,679605	64
**CRC cells**	SNHG29	79,54991	2,8617706	65
**CRC cells**	GGH	79,290146	5,4411983	66
**CRC cells**	CKB	79,26323	6,2637815	67
**CRC cells**	HSPA1B	79,19186	3,4463696	68
**CRC cells**	RPL37	79,157875	2,1816564	69
**CRC cells**	NCKAP1	78,91418	4,1100364	70
**CRC cells**	FARP1	78,66292	4,059554	71
**CRC cells**	MEST	78,56062	5,9615383	72
**CRC cells**	GOLM1	78,35279	4,4018674	73
**CRC cells**	TMEM45B	78,32657	4,6062646	74
**CRC cells**	LAPTM4B	78,30763	4,189996	75
**CRC cells**	CTTN	78,192154	4,4528375	76
**CRC cells**	TSPAN13	78,14984	4,299789	77
**CRC cells**	YWHAE	77,95042	2,5689094	78
**CRC cells**	WEE1	77,77606	3,9162853	79
**CRC cells**	ENAH	77,64002	4,271567	80
**CRC cells**	SOX9	77,49095	5,945112	81
**CRC cells**	PHGR1	77,216736	6,07767	82
**CRC cells**	OCLN	77,10117	4,6681433	83
**CRC cells**	VEGFA	76,93758	3,1739333	84
**CRC cells**	CNN3	76,91871	4,329194	85
**CRC cells**	ODC1	76,75377	3,3150692	86
**CRC cells**	PPA1	76,489815	2,9512281	87
**CRC cells**	PPP1R1B	76,45238	6,111219	88
**CRC cells**	MYH14	76,43446	4,4775505	89
**CRC cells**	AFDN	76,35447	3,2323627	90
**CRC cells**	ANXA4	76,13002	2,9269392	91
**CRC cells**	GDF15	75,926346	5,276383	92
**CRC cells**	ZFAND1	75,69116	3,2968535	93
**CRC cells**	PCBD1	75,54567	3,4830108	94
**CRC cells**	HSP90AA1	75,43839	2,019755	95
**CRC cells**	TMEM176A	75,38653	3,4598587	96
**CRC cells**	SLC12A2	75,2467	3,7219477	97
**CRC cells**	NPM1	75,065765	2,1913617	98
**CRC cells**	HDAC2	74,946	2,8191185	99
**CRC cells**	SPTBN1	74,7898	3,0154395	100
**Endothelial cells**	IGFBP7	49,3588	7,5536084	1
**Endothelial cells**	APP	49,103546	5,069895	2
**Endothelial cells**	SPARC	46,63725	7,5813527	3
**Endothelial cells**	IGFBP4	45,522755	6,192212	4
**Endothelial cells**	CNN3	45,04166	5,9788346	5
**Endothelial cells**	PDLIM1	44,34771	5,07567	6

**Endothelial cells**	PTPRB	43,538895	8,603123	7
**Endothelial cells**	ADGRL4	43,50393	8,577135	8
**Endothelial cells**	FLT1	43,33022	6,741198	9
**Endothelial cells**	COL4A1	42,457253	8,371736	10
**Endothelial cells**	IL6ST	42,00137	4,044933	11
**Endothelial cells**	TCF4	41,77452	4,999016	12
**Endothelial cells**	TM4SF1	41,45584	6,133519	13
**Endothelial cells**	CD59	41,296238	4,4618187	14
**Endothelial cells**	CAVIN1	41,097343	7,3584127	15
**Endothelial cells**	LDB2	40,904865	5,333075	16
**Endothelial cells**	EPAS1	40,868137	5,3346634	17
**Endothelial cells**	COL4A2	40,784306	7,5254235	18
**Endothelial cells**	ADGRF5	40,44308	9,087503	19
**Endothelial cells**	CDH5	40,33241	9,2148	20
**Endothelial cells**	NID1	40,279182	7,622175	21
**Endothelial cells**	CAVIN2	40,19103	8,829627	22
**Endothelial cells**	TIMP3	40,083733	7,1830096	23
**Endothelial cells**	GNG11	39,81272	9,437655	24
**Endothelial cells**	A2M	39,700344	5,871944	25
**Endothelial cells**	TGM2	39,39844	5,0819693	26
**Endothelial cells**	HSPG2	39,31843	7,849347	27
**Endothelial cells**	PEA15	39,069965	3,3869452	28
**Endothelial cells**	PRSS23	38,943516	5,617778	29
**Endothelial cells**	MARCKSL1	38,66455	3,8906324	30
**Endothelial cells**	SHANK3	38,633987	7,8593655	31
**Endothelial cells**	DNASE1L3	38,51767	9,180404	32
**Endothelial cells**	IFITM3	38,185375	2,9942613	33
**Endothelial cells**	S100A16	38,108383	6,3103223	34
**Endothelial cells**	PLPP3	38,024	5,7526693	35
**Endothelial cells**	MCAM	38,00059	6,241577	36
**Endothelial cells**	CAV1	37,83755	7,867988	37
**Endothelial cells**	TIE1	37,790195	6,3858266	38
**Endothelial cells**	AKAP12	37,725895	7,01737	39
**Endothelial cells**	TFPI	37,597664	6,27738	40
**Endothelial cells**	IL33	37,382763	7,8057556	41
**Endothelial cells**	PDE2A	37,262478	7,2903547	42
**Endothelial cells**	ESAM	37,195717	8,10372	43
**Endothelial cells**	ITGB1	37,117504	2,5617085	44
**Endothelial cells**	SELENOP	37,065205	5,217833	45
**Endothelial cells**	FKBP1A	36,533234	2,7684908	46
**Endothelial cells**	TINAGL1	36,401432	5,6211762	47
**Endothelial cells**	PRCP	36,317978	4,042716	48
**Endothelial cells**	NR2F2	36,313377	6,1951113	49
**Endothelial cells**	WWTR1	36,24661	6,6512966	50

**Endothelial cells**	PECAM1	36,199512	3,5764534	51
**Endothelial cells**	CALCRL	35,524143	7,4344406	52
**Endothelial cells**	CD93	35,328457	3,3128817	53
**Endothelial cells**	ANXA2	35,21127	2,7304041	54
**Endothelial cells**	S1PR1	35,181892	4,577881	55
**Endothelial cells**	RAMP2	35,179817	8,0648985	56
**Endothelial cells**	TSPAN7	35,162655	6,4884334	57
**Endothelial cells**	STOM	34,876312	2,667534	58
**Endothelial cells**	MMRN2	34,849144	9,846572	59
**Endothelial cells**	IFI27	34,066715	6,2047963	60
**Endothelial cells**	FZD4	33,974743	9,040481	61
**Endothelial cells**	CLIC4	33,904324	4,1401906	62
**Endothelial cells**	NRP1	33,852028	4,079702	63
**Endothelial cells**	SH3BP5	33,40652	3,0302315	64
**Endothelial cells**	EMCN	33,077602	7,74741	65
**Endothelial cells**	PALM2AKAP2	33,03573	3,7213707	66
**Endothelial cells**	ROBO4	32,976772	9,855466	67
**Endothelial cells**	INSR	32,87174	4,300042	68
**Endothelial cells**	NFIB	32,691463	4,7130585	69
**Endothelial cells**	ENG	32,599087	4,9675717	70
**Endothelial cells**	YWHAE	32,32959	2,1720076	71
**Endothelial cells**	ARHGAP29	32,263012	6,411768	72
**Endothelial cells**	KDR	32,05266	9,860882	73
**Endothelial cells**	RNASE1	32,018562	4,5979266	74
**Endothelial cells**	SASH1	31,997417	4,461494	75
**Endothelial cells**	TPM4	31,952557	2,094222	76
**Endothelial cells**	NRP2	31,921118	4,8342137	77
**Endothelial cells**	RHOJ	31,875046	8,75448	78
**Endothelial cells**	TIMP2	31,773848	2,7639954	79
**Endothelial cells**	TUBB6	31,52411	4,4795113	80
**Endothelial cells**	PPFIBP1	31,344387	5,2024746	81
**Endothelial cells**	PLS3	31,232687	5,550468	82
**Endothelial cells**	GNG12	31,18177	4,3645096	83
**Endothelial cells**	CCDC50	31,074598	3,6539564	84
**Endothelial cells**	RDX	30,995441	2,9849594	85
**Endothelial cells**	CTSL	30,686707	3,6052668	86
**Endothelial cells**	PCAT19	30,538181	9,301824	87
**Endothelial cells**	F8	30,282637	5,8187222	88
**Endothelial cells**	YES1	30,20169	3,4014194	89
**Endothelial cells**	HYAL2	30,106882	7,068376	90
**Endothelial cells**	TACC1	29,917976	2,3235552	91
**Endothelial cells**	TEK	29,879477	8,3238535	92
**Endothelial cells**	MYCT1	29,87742	10,179217	93
**Endothelial cells**	DLC1	29,354553	5,707417	94

**Endothelial cells**	ST6GAL1	29,347958	2,942412	95
**Endothelial cells**	CYYR1	29,225147	6,6682525	96
**Endothelial cells**	BMPR2	29,143599	3,0790765	97
**Endothelial cells**	PXDN	29,13108	7,461935	98
**Endothelial cells**	FCN3	29,116974	9,929073	99
**Endothelial cells**	STAB1	29,11439	4,1868114	100
**Fibroblasts**	SPARC	29,738832	8,693415	1
**Fibroblasts**	CALD1	29,572153	8,418486	2
**Fibroblasts**	IGFBP7	28,927418	7,702381	3
**Fibroblasts**	COL3A1	28,417534	11,86341	4
**Fibroblasts**	IGFBP4	28,350689	6,3715324	5
**Fibroblasts**	COL1A2	28,328606	11,819385	6
**Fibroblasts**	COL1A1	28,07215	11,004207	7
**Fibroblasts**	ITGB1	26,869125	3,5559244	8
**Fibroblasts**	C1R	26,777657	8,048489	9
**Fibroblasts**	CAVIN1	26,755793	6,8484178	10
**Fibroblasts**	FN1	26,49177	7,306832	11
**Fibroblasts**	BGN	25,858837	9,291638	12
**Fibroblasts**	CNN3	25,682184	5,446522	13
**Fibroblasts**	C1S	25,58914	7,3729014	14
**Fibroblasts**	TPM1	25,58404	5,254236	15
**Fibroblasts**	FSTL1	25,49588	8,223429	16
**Fibroblasts**	COL5A2	25,17772	9,459306	17
**Fibroblasts**	CLIC4	24,995546	4,498282	18
**Fibroblasts**	COL6A1	24,879232	7,340275	19
**Fibroblasts**	SERPING1	24,687872	5,208859	20
**Fibroblasts**	CALU	24,469051	4,138177	21
**Fibroblasts**	ANTXR1	24,32827	8,008465	22
**Fibroblasts**	NR2F2	24,187035	5,870645	23
**Fibroblasts**	PARVA	24,129128	5,845154	24
**Fibroblasts**	MT2A	24,124264	4,5965257	25
**Fibroblasts**	PLS3	24,073648	5,7401094	26
**Fibroblasts**	DCN	23,968185	10,23467	27
**Fibroblasts**	CAV1	23,95797	6,5874696	28
**Fibroblasts**	LAMC1	23,690172	5,2586017	29
**Fibroblasts**	MGP	23,343647	7,8141336	30
**Fibroblasts**	RBPMS	23,309677	5,2875314	31
**Fibroblasts**	TIMP1	23,286072	4,445024	32
**Fibroblasts**	DSTN	23,251757	3,6142075	33
**Fibroblasts**	CD59	23,227947	3,5760005	34
**Fibroblasts**	APP	23,21137	3,369201	35
**Fibroblasts**	COL6A2	23,210846	6,3149657	36
**Fibroblasts**	C1QTNF1	23,205072	6,4606667	37
**Fibroblasts**	THBS2	23,118835	10,694866	38

**Fibroblasts**	TAGLN	23,069653	7,7844086	39
**Fibroblasts**	IL6ST	23,047737	3,4812858	40
**Fibroblasts**	SERPINH1	23,007158	5,670894	41
**Fibroblasts**	KANK2	22,865747	6,0417314	42
**Fibroblasts**	DDR2	22,780174	7,196745	43
**Fibroblasts**	MYLK	22,623304	6,8552933	44
**Fibroblasts**	GNG12	22,443626	4,4143434	45
**Fibroblasts**	LAMB1	22,388603	6,0800967	46
**Fibroblasts**	CCDC80	22,244476	9,125082	47
**Fibroblasts**	YAP1	22,20139	4,8880677	48
**Fibroblasts**	PLOD2	22,144377	5,28859	49
**Fibroblasts**	TIMP2	22,11753	3,2579622	50
**Fibroblasts**	ACTA2	22,066116	6,547726	51
**Fibroblasts**	LGALS3BP	22,056488	4,0162187	52
**Fibroblasts**	VIM	22,040636	2,7665932	53
**Fibroblasts**	EPAS1	21,973755	4,440654	54
**Fibroblasts**	MAP1B	21,936398	4,3680058	55
**Fibroblasts**	TNS1	21,92969	4,9913197	56
**Fibroblasts**	COL4A2	21,792643	6,526531	57
**Fibroblasts**	OSMR	21,790741	6,2244735	58
**Fibroblasts**	NFIX	21,773777	5,2182226	59
**Fibroblasts**	TPM2	21,769257	5,175086	60
**Fibroblasts**	FERMT2	21,700165	4,995918	61
**Fibroblasts**	PALM2AKAP2	21,693344	3,6625636	62
**Fibroblasts**	KIRREL1	21,632101	7,016695	63
**Fibroblasts**	COL5A1	21,570824	7,226681	64
**Fibroblasts**	LGALS1	21,424341	3,440562	65
**Fibroblasts**	ITGAV	21,38971	4,002083	66
**Fibroblasts**	VCL	21,356459	3,4821167	67
**Fibroblasts**	SLC39A14	21,324118	5,0028124	68
**Fibroblasts**	TPM4	21,123484	2,4607928	69
**Fibroblasts**	MSRB3	20,928656	6,356281	70
**Fibroblasts**	TIMP3	20,916525	5,710028	71
**Fibroblasts**	TCEAL9	20,884989	4,3591046	72
**Fibroblasts**	CYBRD1	20,781559	4,466992	73
**Fibroblasts**	RTL8C	20,715456	3,0924761	74
**Fibroblasts**	NCKAP1	20,693098	3,7991717	75
**Fibroblasts**	BEX3	20,572817	4,470231	76
**Fibroblasts**	PXDN	20,466461	6,558321	77
**Fibroblasts**	YBX3	20,4644	2,915355	78
**Fibroblasts**	CRTAP	20,460627	2,8577485	79
**Fibroblasts**	IFITM3	20,409742	2,7094753	80
**Fibroblasts**	NDN	20,311901	6,9489093	81
**Fibroblasts**	COL4A1	20,305962	6,807719	82

**Fibroblasts**	MAP3K20	20,257591	3,3902667	83
**Fibroblasts**	LAMA4	20,233477	6,7895947	84
**Fibroblasts**	PDLIM5	20,118261	3,090248	85
**Fibroblasts**	FILIP1L	20,058998	4,8181105	86
**Fibroblasts**	SERPINE1	20,00795	6,62901	87
**Fibroblasts**	LHFPL6	20,001144	5,930048	88
**Fibroblasts**	ANXA5	19,944561	2,6501155	89
**Fibroblasts**	PDLIM1	19,90528	3,2428334	90
**Fibroblasts**	IL1R1	19,863785	3,8656087	91
**Fibroblasts**	AEBP1	19,806225	7,547874	92
**Fibroblasts**	ACTN1	19,79015	2,6771057	93
**Fibroblasts**	CDH11	19,737322	7,995508	94
**Fibroblasts**	NNMT	19,732363	6,120561	95
**Fibroblasts**	TCF4	19,725346	3,2991946	96
**Fibroblasts**	RBFOX2	19,662405	2,900709	97
**Fibroblasts**	SPTBN1	19,659575	2,9079492	98
**Fibroblasts**	C11orf96	19,535833	6,7703757	99
**Fibroblasts**	PALLD	19,494915	4,1542797	100
**Mast cells**	TPSAB1	16,870985	15,107539	1
**Mast cells**	TPSB2	16,870583	14,461239	2
**Mast cells**	KIT	16,867485	11,40319	3
**Mast cells**	VWA5A	16,840406	8,408566	4
**Mast cells**	CPA3	16,69256	14,926282	5
**Mast cells**	MS4A2	16,51264	13,462307	6
**Mast cells**	GATA2	16,507849	11,038025	7
**Mast cells**	SLC18A2	16,155832	12,069466	8
**Mast cells**	HPGDS	15,961134	10,165441	9
**Mast cells**	SIGLEC6	15,250736	10,571847	10
**Mast cells**	CLU	15,160985	6,3024054	11
**Mast cells**	ACSL4	15,049737	4,795363	12
**Mast cells**	IL1RL1	14,999447	8,451588	13
**Mast cells**	HPGD	14,876578	6,8222275	14
**Mast cells**	PTGS1	14,649866	6,2465773	15
**Mast cells**	GCSAML	14,548883	11,070553	16
**Mast cells**	PALM2AKAP2	14,489881	4,698437	17
**Mast cells**	LAPTM4A	14,417456	3,4721537	18
**Mast cells**	VIM	14,265004	3,2391798	19
**Mast cells**	HDC	14,200444	12,336419	20
**Mast cells**	RHEX	14,166569	9,434622	21
**Mast cells**	MAOB	14,140494	8,513491	22
**Mast cells**	LMO4	13,855301	4,062287	23
**Mast cells**	BMP2K	13,7538185	4,16988	24
**Mast cells**	BACE2	13,747196	5,377097	25
**Mast cells**	ENSG0000023	13,485618	11,195343	26

**Mast cells**	SLC45A3	13,243836	8,380471	27
**Mast cells**	CD9	13,059216	4,191793	28
**Mast cells**	ARHGEF6	13,034036	3,8444314	29
**Mast cells**	RGS13	12,936534	9,852229	30
**Mast cells**	SLC24A3	12,872852	6,6023383	31
**Mast cells**	ANXA1	12,783519	3,3008933	32
**Mast cells**	SIGLEC17P	12,542313	5,4237666	33
**Mast cells**	IL18R1	12,249055	3,419246	34
**Mast cells**	AHNAK	12,166004	2,749665	35
**Mast cells**	TIMP3	11,863167	5,211858	36
**Mast cells**	RGS1	11,860458	3,9530754	37
**Mast cells**	SLC44A1	11,692638	3,4402742	38
**Mast cells**	GALC	11,537975	4,0568037	39
**Mast cells**	BHLHE40	11,481146	3,07937	40
**Mast cells**	TESPA1	11,412116	4,0642066	41
**Mast cells**	ARHGAP18	11,261622	3,117947	42
**Mast cells**	SEPTIN2	11,17096	2,3065703	43
**Mast cells**	GRAP2	11,167454	3,7081587	44
**Mast cells**	CAVIN2	11,103228	6,309902	45
**Mast cells**	LPCAT2	10,992721	3,328177	46
**Mast cells**	CD63	10,973767	2,2044244	47
**Mast cells**	CD84	10,718358	3,0623846	48
**Mast cells**	BTK	10,692957	4,1457148	49
**Mast cells**	PBX1	10,69182	5,3828993	50
**Mast cells**	SWAP70	10,587412	3,436583	51
**Mast cells**	RAB27B	10,541411	4,600569	52
**Mast cells**	LEO1	10,379375	3,7156808	53
**Mast cells**	EPB41L1	10,328143	4,7739344	54
**Mast cells**	SMYD3	10,114768	3,4409125	55
**Mast cells**	RHOBTB3	10,047145	3,7400274	56
**Mast cells**	LIF	10,034518	5,44301	57
**Mast cells**	SAMSN1	9,929759	2,443231	58
**Mast cells**	SIGLEC8	9,929297	10,031883	59
**Mast cells**	CAPG	9,827789	3,3247924	60
**Mast cells**	RGS2	9,740195	2,9757113	61
**Mast cells**	CALB2	9,734636	9,091115	62
**Mast cells**	ITM2C	9,698386	2,701476	63
**Mast cells**	TMEM176A	9,684335	3,5029988	64
**Mast cells**	PTMA	9,654704	1,5315807	65
**Mast cells**	ATP6V0A2	9,617376	3,6818795	66
**Mast cells**	MT-ND5	9,595514	1,2919194	67
**Mast cells**	ALS2	9,539348	4,1053543	68
**Mast cells**	RAPGEF2	9,43902	2,7033277	69
**Mast cells**	FER	9,43612	4,0787864	70

**Mast cells**	ALDH1A1	9,364725	3,946974	71
**Mast cells**	ABCC4	9,305744	4,736164	72
**Mast cells**	HS6ST1	9,267206	5,6010923	73
**Mast cells**	CD22	9,190006	5,233742	74
**Mast cells**	ALOX5	9,124343	2,6833572	75
**Mast cells**	GPR65	9,03423	2,3495474	76
**Mast cells**	MLPH	8,997388	6,008423	77
**Mast cells**	TNIK	8,990522	2,7199922	78
**Mast cells**	CSF1	8,951938	4,030495	79
**Mast cells**	FOSB	8,914953	2,857652	80
**Mast cells**	RPL37A	8,898403	1,5434753	81
**Mast cells**	PRNP	8,892137	2,427467	82
**Mast cells**	AHR	8,858252	2,919231	83
**Mast cells**	TMEM176B	8,841013	3,4907036	84
**Mast cells**	CDK15	8,836586	8,367127	85
**Mast cells**	ACER3	8,697541	2,85496	86
**Mast cells**	EEF1A1	8,696533	1,4906058	87
**Mast cells**	PEBP1	8,66132	2,131427	88
**Mast cells**	SMIM3	8,600394	3,1876373	89
**Mast cells**	KREMEN1	8,581963	3,1111586	90
**Mast cells**	MT-ND4	8,576221	0,85960156	91
**Mast cells**	DLC1	8,526189	4,620294	92
**Mast cells**	SOX13	8,5125	5,2863135	93
**Mast cells**	UBB	8,480554	1,6249524	94
**Mast cells**	FOXP1	8,433353	2,3061242	95
**Mast cells**	ADRB2	8,4189205	3,461985	96
**Mast cells**	ALAS1	8,416739	3,5408444	97
**Mast cells**	TNS1	8,416546	4,1704297	98
**Mast cells**	MT-ND4L	8,381621	1,1703092	99
**Mast cells**	CTSG	8,3348465	10,578119	100
**Monocytes/Macrophages**	CTSB	159,8552	5,64562	1
**Monocytes/Macrophages**	PSAP	153,75725	3,9547153	2
**Monocytes/Macrophages**	FTL	152,31668	3,8616648	3
**Monocytes/Macrophages**	CYBB	146,55603	5,5843344	4
**Monocytes/Macrophages**	HLA-DRA	146,21011	5,7835	5
**Monocytes/Macrophages**	NPC2	141,95238	4,4450636	6
**Monocytes/Macrophages**	CD163	139,3323	6,3012037	7
**Monocytes/Macrophages**	MAFB	137,22859	6,2402225	8
**Monocytes/Macrophages**	ANXA5	136,73462	3,603629	9
**Monocytes/Macrophages**	LYZ	135,33972	5,01261	10
**Monocytes/Macrophages**	HLA-DRB1	133,59232	4,548119	11
**Monocytes/Macrophages**	MPEG1	132,98138	5,330738	12
**Monocytes/Macrophages**	CD74	132,55594	3,7290251	13
**Monocytes/Macrophages**	VCAN	131,13007	6,4509273	14

**Monocytes/Macrophages**	TGFBI	129,2771	5,178731	15
**Monocytes/Macrophages**	VIM	129,17604	2,978114	16
**Monocytes/Macrophages**	CST3	127,80486	4,0122194	17
**Monocytes/Macrophages**	MS4A7	126,927	6,4730797	18
**Monocytes/Macrophages**	HMOX1	126,48955	5,575038	19
**Monocytes/Macrophages**	APLP2	126,34113	3,1623275	20
**Monocytes/Macrophages**	AIF1	125,90145	3,8614082	21
**Monocytes/Macrophages**	CTSS	124,70037	3,2035801	22
**Monocytes/Macrophages**	CPVL	124,66431	5,6347046	23
**Monocytes/Macrophages**	CCDC88A	122,82679	4,108627	24
**Monocytes/Macrophages**	ANXA2	122,80993	3,1128576	25
**Monocytes/Macrophages**	GRN	122,56745	3,327052	26
**Monocytes/Macrophages**	KCTD12	122,447716	3,7712975	27
**Monocytes/Macrophages**	CREG1	122,19121	3,8564334	28
**Monocytes/Macrophages**	MFSD1	121,6189	3,3918283	29
**Monocytes/Macrophages**	TPP1	117,981514	2,710755	30
**Monocytes/Macrophages**	FCER1G	117,85831	3,1716194	31
**Monocytes/Macrophages**	CD14	116,9661	4,1175466	32
**Monocytes/Macrophages**	LIPA	116,95431	4,2989717	33
**Monocytes/Macrophages**	CSF1R	116,729034	5,9772654	34
**Monocytes/Macrophages**	HLA-DPA1	116,12723	3,884034	35
**Monocytes/Macrophages**	LILRB1	115,45493	5,0365586	36
**Monocytes/Macrophages**	CCR1	115,41916	3,4303265	37
**Monocytes/Macrophages**	MS4A6A	115,25704	4,186359	38
**Monocytes/Macrophages**	CTSL	114,93179	5,685294	39
**Monocytes/Macrophages**	CD86	114,234146	5,1816225	40
**Monocytes/Macrophages**	ZEB2	114,149475	3,089895	41
**Monocytes/Macrophages**	KYNU	111,829155	4,844085	42
**Monocytes/Macrophages**	PKM	109,61253	2,6271381	43
**Monocytes/Macrophages**	MARCHF1	109,25502	4,214604	44
**Monocytes/Macrophages**	CTSH	109,08117	3,9591906	45
**Monocytes/Macrophages**	CD300E	108,15811	6,183904	46
**Monocytes/Macrophages**	FTH1	107,53544	2,6908789	47
**Monocytes/Macrophages**	SAMHD1	107,464386	2,5575821	48
**Monocytes/Macrophages**	GSTO1	107,432175	2,8454096	49
**Monocytes/Macrophages**	CD63	107,41903	2,2723715	50
**Monocytes/Macrophages**	CTSZ	107,05718	3,6207955	51
**Monocytes/Macrophages**	CSTB	107,02526	2,7867773	52
**Monocytes/Macrophages**	PLIN2	106,72002	3,3096275	53
**Monocytes/Macrophages**	SLC7A7	106,53121	4,8481784	54
**Monocytes/Macrophages**	LILRB2	106,17045	3,356242	55
**Monocytes/Macrophages**	C1orf162	105,34284	3,060126	56
**Monocytes/Macrophages**	AP1S2	105,20623	2,8601286	57
**Monocytes/Macrophages**	TIMP2	104,35779	2,8272638	58

**Monocytes/Macrophages**	RAB31	103,87631	2,7587092	59
**Monocytes/Macrophages**	MACROH2A1	102,60972	2,3086925	60
**Monocytes/Macrophages**	PNKD	102,514824	3,9196389	61
**Monocytes/Macrophages**	GLUL	102,48884	2,6557307	62
**Monocytes/Macrophages**	MS4A4A	102,4379	5,664214	63
**Monocytes/Macrophages**	DMXL2	102,12238	3,7060158	64
**Monocytes/Macrophages**	EPB41L3	102,07283	5,5716496	65
**Monocytes/Macrophages**	TTYH3	102,031044	3,9642994	66
**Monocytes/Macrophages**	LGALS1	102,009575	3,5092058	67
**Monocytes/Macrophages**	PLXDC2	101,643585	2,9905014	68
**Monocytes/Macrophages**	TIMP1	101,527794	3,591239	69
**Monocytes/Macrophages**	RNF130	101,358734	2,4526072	70
**Monocytes/Macrophages**	IFNGR2	100,50453	2,541084	71
**Monocytes/Macrophages**	YBX1	100,02682	1,9973692	72
**Monocytes/Macrophages**	LILRB4	99,68663	5,877996	73
**Monocytes/Macrophages**	SIRPA	99,46198	2,7911017	74
**Monocytes/Macrophages**	TFEC	99,35619	4,536218	75
**Monocytes/Macrophages**	HLA-DPB1	99,14358	3,1901982	76
**Monocytes/Macrophages**	ASAH1	98,918396	2,4226513	77
**Monocytes/Macrophages**	C3AR1	98,84102	3,0408835	78
**Monocytes/Macrophages**	CXCL16	98,40898	2,6353626	79
**Monocytes/Macrophages**	CLEC7A	98,22482	2,5921836	80
**Monocytes/Macrophages**	SCARB2	97,997955	3,0484223	81
**Monocytes/Macrophages**	ATP6V1B2	97,657585	2,3742867	82
**Monocytes/Macrophages**	LGALS3	97,64911	2,6962342	83
**Monocytes/Macrophages**	CD4	97,485664	2,974399	84
**Monocytes/Macrophages**	PLA2G7	97,22926	5,5891795	85
**Monocytes/Macrophages**	DAB2	97,13018	4,107845	86
**Monocytes/Macrophages**	ADAP2	96,97998	5,190961	87
**Monocytes/Macrophages**	LTA4H	96,909355	2,670905	88
**Monocytes/Macrophages**	GNS	96,790886	2,5784798	89
**Monocytes/Macrophages**	EMILIN2	96,482086	4,280184	90
**Monocytes/Macrophages**	UCP2	96,38567	2,614689	91
**Monocytes/Macrophages**	PGD	96,211006	2,598784	92
**Monocytes/Macrophages**	MSR1	95,75728	5,4362507	93
**Monocytes/Macrophages**	CORO1C	95,57519	2,8602839	94
**Monocytes/Macrophages**	HEXB	95,570206	2,5821056	95
**Monocytes/Macrophages**	ATP6V1F	95,561264	2,595789	96
**Monocytes/Macrophages**	CPM	95,417534	4,3027806	97
**Monocytes/Macrophages**	CYFIP1	95,32518	3,7651508	98
**Monocytes/Macrophages**	C5AR1	95,26111	2,8688152	99
**Monocytes/Macrophages**	IGSF6	95,176025	2,7023833	100
**Neutrophils**	NAMPT	189,17526	5,885352	1
**Neutrophils**	CSF3R	184,17589	6,6795936	2

**Neutrophils**	SRGN	181,19241	3,5340407	3
**Neutrophils**	IFITM2	178,97835	4,674087	4
**Neutrophils**	FCGR3B	177,6775	8,78761	5
**Neutrophils**	FPR1	175,58572	5,771691	6
**Neutrophils**	SLC25A37	172,3877	5,6529126	7
**Neutrophils**	S100A8	169,62126	6,3511314	8
**Neutrophils**	DUSP1	168,8575	4,136819	9
**Neutrophils**	S100A11	168,74431	3,6581173	10
**Neutrophils**	G0S2	168,69286	7,075954	11
**Neutrophils**	SAT1	168,57588	4,290012	12
**Neutrophils**	BASP1	167,25996	5,6398697	13
**Neutrophils**	CXCR2	165,13567	8,132133	14
**Neutrophils**	LITAF	165,03769	3,5354326	15
**Neutrophils**	AQP9	164,84538	5,3570204	16
**Neutrophils**	RNF149	164,20297	3,7492723	17
**Neutrophils**	SOD2	163,77423	4,3953514	18
**Neutrophils**	MXD1	162,18715	4,8265634	19
**Neutrophils**	RGS2	161,02708	5,173565	20
**Neutrophils**	FOS	160,51962	3,9483476	21
**Neutrophils**	FTH1	159,53593	3,4078674	22
**Neutrophils**	BCL2A1	156,37012	4,663924	23
**Neutrophils**	CEBPB	156,25565	3,8545406	24
**Neutrophils**	CLEC4E	155,22452	5,528726	25
**Neutrophils**	IL1R2	153,11124	6,1804986	26
**Neutrophils**	ALOX5AP	151,97964	3,4099536	27
**Neutrophils**	MCL1	151,13805	2,4470685	28
**Neutrophils**	C5AR1	150,77762	4,6766367	29
**Neutrophils**	GCA	150,55493	4,961528	30
**Neutrophils**	H3-3A	148,52702	2,7278757	31
**Neutrophils**	MNDA	147,86057	5,091335	32
**Neutrophils**	S100A9	147,81006	5,0022235	33
**Neutrophils**	SDCBP	146,91768	3,0601668	34
**Neutrophils**	ITM2B	146,89171	2,1000886	35
**Neutrophils**	SERPINA1	144,58357	3,882451	36
**Neutrophils**	H3-3B	144,56012	1,9955832	37
**Neutrophils**	CD55	144,47896	3,8046646	38
**Neutrophils**	VNN 2,00	143,78247	5,787263	39
**Neutrophils**	ACSL1	140,43822	4,4701686	40
**Neutrophils**	RIPOR2	139,3286	3,5677962	41
**Neutrophils**	NCF2	139,32787	4,1020117	42
**Neutrophils**	NAMPTP1	138,60939	5,0370317	43
**Neutrophils**	SELL	137,95728	4,7482615	44
**Neutrophils**	TREM1	133,87282	4,7979455	45
**Neutrophils**	MALAT1	133,10603	1,6266546	46

**Neutrophils**	SPI1	132,99254	3,7426486	47
**Neutrophils**	EVI2B	130,02106	2,5648932	48
**Neutrophils**	SMAP2	129,34885	2,680806	49
**Neutrophils**	LYN	129,02443	3,0726306	50
**Neutrophils**	HLA-C	128,9754	1,3343937	51
**Neutrophils**	MT-RNR2	128,8495	1,1343164	52
**Neutrophils**	SAMSN1	126,37038	3,4179275	53
**Neutrophils**	IRS2	126,28766	3,767744	54
**Neutrophils**	CXCR1	126,12755	7,6130314	55
**Neutrophils**	NIBAN1	125,82693	3,302885	56
**Neutrophils**	ARRB2	123,567825	2,5162303	57
**Neutrophils**	JUNB	122,22232	2,5185938	58
**Neutrophils**	PLAUR	120,18325	3,6758595	59
**Neutrophils**	SORL1	119,29743	2,9425225	60
**Neutrophils**	MT-RNR1	117,81886	1,2462674	61
**Neutrophils**	NEAT1	117,61716	1,9657153	62
**Neutrophils**	TYROBP	116,386055	2,6188939	63
**Neutrophils**	RTN3	112,38429	2,3277178	64
**Neutrophils**	LILRB3	112,37877	4,1272035	65
**Neutrophils**	TLR2	112,16216	3,54605	66
**Neutrophils**	CSF2RB	111,53134	4,3105044	67
**Neutrophils**	SLC11A1	111,49699	3,9422135	68
**Neutrophils**	GLUL	111,457	2,7912028	69
**Neutrophils**	HCLS1	111,398026	1,9224721	70
**Neutrophils**	SIRPB1	111,305786	4,312299	71
**Neutrophils**	USP10	111,30398	3,1812255	72
**Neutrophils**	BCL6	110,74844	3,613013	73
**Neutrophils**	CXCL8	110,34079	4,514762	74
**Neutrophils**	DOK3	109,11791	4,0623784	75
**Neutrophils**	VASP	109,057014	2,293044	76
**Neutrophils**	PELI1	107,96317	3,9406755	77
**Neutrophils**	SLC2A3	106,10864	2,3941114	78
**Neutrophils**	FPR2	105,32899	4,8023233	79
**Neutrophils**	PTGS2	105,09581	4,950327	80
**Neutrophils**	CR1	104,22062	4,0696845	81
**Neutrophils**	CMTM2	103,92049	7,3120384	82
**Neutrophils**	MIR223HG	102,81046	4,982519	83
**Neutrophils**	KCNJ15	102,524605	5,5504417	84
**Neutrophils**	APOBEC3A	102,5157	4,662973	85
**Neutrophils**	IGSF6	102,22753	3,1724946	86
**Neutrophils**	SLA	101,986176	1,9100522	87
**Neutrophils**	ACTB	101,947365	1,049701	88
**Neutrophils**	FGR	100,79252	2,499983	89
**Neutrophils**	MYO1F	100,51298	2,2612593	90

**Neutrophils**	SEC14L1	100,46273	2,6311069	91
**Neutrophils**	CTSS	100,40017	2,1875744	92
**Neutrophils**	MME	99,90422	5,2869363	93
**Neutrophils**	TMEM154	99,77215	3,9853232	94
**Neutrophils**	IRAK3	99,39079	3,7213795	95
**Neutrophils**	MEGF9	99,13572	3,1529276	96
**Neutrophils**	PHC2	98,22506	2,9698155	97
**Neutrophils**	BTG2	97,93291	2,2583268	98
**Neutrophils**	EGR1	97,68465	3,1640162	99
**Neutrophils**	LRRK2	97,17471	4,0784235	100
**NK cells**	IL2RB	149,83545	4,659185	1
**NK cells**	KLRD1	145,31586	4,781021	2
**NK cells**	NKG7	144,9491	4,4058433	3
**NK cells**	KLRF1	136,71577	6,5041184	4
**NK cells**	PRF1	119,26729	3,663724	5
**NK cells**	GZMA	117,36784	3,33216	6
**NK cells**	ETS1	112,22061	2,7591162	7
**NK cells**	TXK	111,406425	4,766466	8
**NK cells**	EOMES	110,24309	3,9516978	9
**NK cells**	SH2D1B	108,66734	7,08418	10
**NK cells**	IKZF3	105,991104	2,8696277	11
**NK cells**	KLRB1	104,59673	3,1363392	12
**NK cells**	CCL4	101,75958	2,9099903	13
**NK cells**	CD247	99,98469	3,568701	14
**NK cells**	NCR1	99,934395	5,3877244	15
**NK cells**	CMC1	98,23791	3,5680618	16
**NK cells**	PYHIN1	97,18487	3,1260097	17
**NK cells**	RUNX3	96,61866	2,4365873	18
**NK cells**	FYN	96,086395	2,366012	19
**NK cells**	SLFN5	94,848946	2,5244813	20
**NK cells**	MT-CO1	94,39452	1,3119881	21
**NK cells**	CTSW	91,17788	3,1139383	22
**NK cells**	CCL3	90,90909	3,2176962	23
**NK cells**	MYBL1	89,30299	3,0643845	24
**NK cells**	PIP4K2A	87,59515	1,9937719	25
**NK cells**	CCL5	87,09812	2,7436235	26
**NK cells**	CD96	85,595535	2,3374057	27
**NK cells**	CD160	85,50885	4,7422256	28
**NK cells**	PRKCH	85,2824	2,2936208	29
**NK cells**	TRDC	85,25182	4,2151475	30
**NK cells**	SLFN12L	84,710785	2,4349055	31
**NK cells**	MT-ND4L	83,27329	1,1878985	32
**NK cells**	ID2	81,85933	1,9253893	33
**NK cells**	AOAH	80,63336	2,130441	34

**NK cells**	MT-CO2	80,61352	0,83987904	35
**NK cells**	PTPN4	79,89481	2,6182952	36
**NK cells**	XCL1	78,62646	4,609391	37
**NK cells**	ZBTB16	78,14951	2,5357783	38
**NK cells**	PTGDR	78,06593	3,7681024	39
**NK cells**	SLAMF7	77,91923	2,2988107	40
**NK cells**	MT-ATP8	77,86048	1,2572427	41
**NK cells**	MT-ND4	77,74165	0,7894769	42
**NK cells**	APMAP	77,548515	1,8194937	43
**NK cells**	SLC38A1	75,07126	1,8451028	44
**NK cells**	APOBEC3G	74,9606	2,4731445	45
**NK cells**	CCND2	74,79783	2,452142	46
**NK cells**	ITGAL	73,331604	2,0887847	47
**NK cells**	CD38	71,76436	3,3449996	48
**NK cells**	XCL2	71,58506	4,1955805	49
**NK cells**	SH2D1A	71,3629	2,508268	50
**NK cells**	ENSG0000029	70,512596	5,6642566	51
**NK cells**	MT-ATP6	70,42198	0,84493375	52
**NK cells**	SLFN13	70,366684	3,6906748	53
**NK cells**	NCAM1	69,92714	4,031389	54
**NK cells**	GIMAP7	69,37371	1,8011038	55
**NK cells**	PIK3R1	68,66276	1,8320833	56
**NK cells**	MAPK1	68,57046	1,7121028	57
**NK cells**	LBH	68,26597	2,0105212	58
**NK cells**	HELZ	67,47919	2,0600567	59
**NK cells**	WIPF1	67,197464	1,1879832	60
**NK cells**	MACF1	67,02425	1,5789274	61
**NK cells**	CD7	66,938545	3,11438	62
**NK cells**	MT-CO3	66,916664	0,7441806	63
**NK cells**	JAK1	66,59128	1,3100177	64
**NK cells**	UTRN	66,35419	1,7817144	65
**NK cells**	STK17A	65,742294	1,8071026	66
**NK cells**	ARHGAP9	64,914055	1,5465662	67
**NK cells**	MT-ND5	64,56176	1,0179042	68
**NK cells**	KLRC1	64,092354	4,7340775	69
**NK cells**	MBNL1	63,927647	1,1341767	70
**NK cells**	LNPEP	62,962494	1,8600844	71
**NK cells**	CLDND1	62,95488	1,9188898	72
**NK cells**	FCMR	62,86477	1,8951627	73
**NK cells**	PRKACB	62,75794	1,9105577	74
**NK cells**	RAC2	61,649593	1,1043195	75
**NK cells**	FASLG	61,495697	2,8561814	76
**NK cells**	ITGA4	61,040024	1,6743591	77
**NK cells**	GFOD1	60,282856	3,3915153	78

**NK cells**	SEPTIN7	59,907288	1,1233616	79
**NK cells**	GNPTAB	59,51415	2,2329092	80
**NK cells**	PLAC8	58,287838	2,1277454	81
**NK cells**	ATM	58,065372	1,6376034	82
**NK cells**	TOX2	58,014626	3,6114109	83
**NK cells**	ABCB1	57,767864	2,6411572	84
**NK cells**	SACM1L	57,06569	2,0370111	85
**NK cells**	DENND2D	56,448433	2,044975	86
**NK cells**	GNLY	55,850975	3,8394704	87
**NK cells**	IPCEF1	55,114597	2,26258	88
**NK cells**	TIGIT	54,655945	2,3427136	89
**NK cells**	PDCD4	54,50311	1,3091576	90
**NK cells**	PITPNC1	54,5001	1,669343	91
**NK cells**	ARPC5L	53,49506	1,9486516	92
**NK cells**	CELF2	53,36084	1,1088291	93
**NK cells**	STAT4	53,350777	1,9086876	94
**NK cells**	SAMD3	53,210247	2,608308	95
**NK cells**	MT-ND3	53,148426	0,6403299	96
**NK cells**	AKNA	52,946983	1,5147696	97
**NK cells**	SLA2	52,92768	2,1882582	98
**NK cells**	HEG1	52,866016	2,6719923	99
**NK cells**	SKAP1	52,858917	1,8047767	100
**Plasma cells**	POU2AF1	32,181805	7,783994	1
**Plasma cells**	HERPUD1	32,099804	4,6287913	2
**Plasma cells**	XBP1	31,624548	4,2397733	3
**Plasma cells**	SEC11C	31,405785	4,5006404	4
**Plasma cells**	HSP90B1	31,252485	3,4324315	5
**Plasma cells**	TENT5C	30,657068	4,4181747	6
**Plasma cells**	SEL1L3	30,632172	4,2271357	7
**Plasma cells**	IGKC	30,205215	9,368473	8
**Plasma cells**	SSR4	30,044645	5,1608677	9
**Plasma cells**	FCRL5	29,146755	7,3790054	10
**Plasma cells**	JCHAIN	28,685951	10,123763	11
**Plasma cells**	CD79A	28,634848	5,9970174	12
**Plasma cells**	PRDX4	28,094423	4,7244406	13
**Plasma cells**	ST6GAL1	27,961506	3,8509483	14
**Plasma cells**	MZB1	27,7499	8,62242	15
**Plasma cells**	SSR3	27,64852	3,1606357	16
**Plasma cells**	PIM2	27,63215	3,8400764	17
**Plasma cells**	FKBP11	27,12507	4,061534	18
**Plasma cells**	SEL1L	26,611507	3,4520772	19
**Plasma cells**	DNAJB9	26,007462	3,4427354	20
**Plasma cells**	ANKRD36BP2	25,858944	7,8397126	21
**Plasma cells**	SPCS2	25,85065	2,673773	22

**Plasma cells**	ITM2C	25,785872	3,598401	23
**Plasma cells**	RPN2	25,217575	2,5773213	24
**Plasma cells**	ERLEC1	25,10863	3,4320657	25
**Plasma cells**	DERL3	25,085762	7,426971	26
**Plasma cells**	RAB30	24,551014	4,4820514	27
**Plasma cells**	LMAN1	24,28789	2,95023	28
**Plasma cells**	CLPTM1L	24,134638	3,0945652	29
**Plasma cells**	UBE2J1	24,005005	2,8826625	30
**Plasma cells**	TP53INP1	23,83403	3,6753395	31
**Plasma cells**	METTL7A	23,750238	3,2828622	32
**Plasma cells**	PLPP5	23,585495	3,2317488	33
**Plasma cells**	SDC1	23,479826	5,946265	34
**Plasma cells**	SPATS2	23,385843	3,7608674	35
**Plasma cells**	SPCS1	23,194277	2,5898266	36
**Plasma cells**	KDELR1	22,798632	2,5944655	37
**Plasma cells**	MEF2C	22,677353	3,0929377	38
**Plasma cells**	CREB3L2	22,675781	3,322087	39
**Plasma cells**	TPD52	22,619486	2,890186	40
**Plasma cells**	TXNDC15	22,589794	3,1559806	41
**Plasma cells**	TRAM1	22,50044	2,105072	42
**Plasma cells**	SPCS3	22,468157	2,278467	43
**Plasma cells**	GNG7	22,460598	4,49723	44
**Plasma cells**	CPNE5	22,323305	5,338003	45
**Plasma cells**	HDLBP	22,30882	2,6534529	46
**Plasma cells**	SYVN1	22,300016	3,408964	47
**Plasma cells**	CPEB4	22,234138	2,7328055	48
**Plasma cells**	SLAMF7	22,008154	2,6445756	49
**Plasma cells**	PDK1	21,969444	3,782253	50
**Plasma cells**	SELENOS	21,916279	2,7099497	51
**Plasma cells**	HSPA5	21,804544	2,243168	52
**Plasma cells**	LINC03034	21,793957	8,672921	53
**Plasma cells**	NUCB2	21,664408	2,5403962	54
**Plasma cells**	TMEM258	21,641104	2,1989017	55
**Plasma cells**	PDIA6	21,619574	2,3134797	56
**Plasma cells**	ADA2	21,592402	2,6040926	57
**Plasma cells**	DENND5B	21,515038	4,736804	58
**Plasma cells**	ENSG0000028	21,488327	9,685136	59
**Plasma cells**	ELL2	21,453085	2,4860044	60
**Plasma cells**	IGHG1	21,378063	10,0123005	61
**Plasma cells**	TRAM2	21,159067	4,1675534	62
**Plasma cells**	MANF	21,00968	2,5731401	63
**Plasma cells**	CLIC4	20,901659	3,1292076	64
**Plasma cells**	COBLL1	20,867306	4,634626	65
**Plasma cells**	MAN1A1	20,86316	2,3850408	66

**Plasma cells**	SEC61B	20,764664	2,4362426	67
**Plasma cells**	IGHGP	20,652256	8,529998	68
**Plasma cells**	CD38	20,496065	2,7841134	69
**Plasma cells**	TMED10	20,2732	1,8821223	70
**Plasma cells**	RALGPS2	20,192617	3,1315577	71
**Plasma cells**	TXNDC11	20,084639	3,0959847	72
**Plasma cells**	TNFRSF17	19,985872	9,859284	73
**Plasma cells**	SELENOK	19,927029	2,2742717	74
**Plasma cells**	SEC62	19,913218	1,8869317	75
**Plasma cells**	SLC1A4	19,777285	3,6164947	76
**Plasma cells**	FBXW7	19,734001	2,4506915	77
**Plasma cells**	SRPRA	19,650948	2,0120792	78
**Plasma cells**	MANEA	19,609503	3,281051	79
**Plasma cells**	CALR	19,561447	1,9979963	80
**Plasma cells**	SRPRB	19,459291	2,7288077	81
**Plasma cells**	VOPP1	19,361073	2,0304441	82
**Plasma cells**	HIPK2	19,288048	2,24178	83
**Plasma cells**	IGHG3	19,230595	10,598022	84
**Plasma cells**	CRELD2	19,101652	2,8836124	85
**Plasma cells**	SERP1	18,94843	1,7534732	86
**Plasma cells**	KDELR2	18,929462	2,1837444	87
**Plasma cells**	ENSG0000024	18,862165	7,421228	88
**Plasma cells**	TMED9	18,851274	1,9738843	89
**Plasma cells**	HSPA13	18,696415	2,5740016	90
**Plasma cells**	PDIA4	18,660635	2,3149557	91
**Plasma cells**	RRBP1	18,64224	2,5517437	92
**Plasma cells**	BHLHE41	18,638626	4,903704	93
**Plasma cells**	HM13	18,593838	2,1492932	94
**Plasma cells**	IGHA1	18,480864	8,145241	95
**Plasma cells**	MYDGF	18,34888	2,5828948	96
**Plasma cells**	CD74	18,263233	1,9094915	97
**Plasma cells**	IGHG2	18,152544	9,925088	98
**Plasma cells**	LARP1B	18,00593	2,550192	99
**Plasma cells**	CUTA	17,879036	2,0604787	100
**T cells CD4**	IL7R	117,82581	4,778542	1
**T cells CD4**	TPT1	106,14957	1,2601565	2
**T cells CD4**	TRAC	96,55905	3,191713	3
**T cells CD4**	RPL34	96,17532	1,7626747	4
**T cells CD4**	RPS29	92,62785	1,789989	5
**T cells CD4**	RPS27	92,61594	1,8374792	6
**T cells CD4**	RPL32	90,48106	1,7513968	7
**T cells CD4**	RPS6	89,76561	1,7171103	8
**T cells CD4**	EEF1A1	89,571365	1,669468	9
**T cells CD4**	CD2	87,43334	2,767182	10

**T cells CD4**	RPS12	87,38907	1,832416	11
**T cells CD4**	RPL31	86,87348	1,6551245	12
**T cells CD4**	RPS27A	86,651596	1,6924134	13
**T cells CD4**	RPL9	85,1714	1,6145217	14
**T cells CD4**	RPL30	85,00175	1,5518782	15
**T cells CD4**	RPL39	83,96633	1,5050669	16
**T cells CD4**	SPOCK2	83,48624	2,681892	17
**T cells CD4**	RPS16	83,065506	1,5785836	18
**T cells CD4**	RPL11	82,99757	1,5893798	19
**T cells CD4**	RPS20	82,93098	1,6137002	20
**T cells CD4**	RPL21	82,31349	1,5681257	21
**T cells CD4**	RPL13	82,01244	1,7336898	22
**T cells CD4**	RPL13A	81,552055	1,6837796	23
**T cells CD4**	RPS25	80,944214	1,6097751	24
**T cells CD4**	RPS4X	80,14467	1,6527478	25
**T cells CD4**	RPL38	78,46842	1,5238184	26
**T cells CD4**	CD3E	78,13339	2,421691	27
**T cells CD4**	NAP1L1	76,80848	1,6764581	28
**T cells CD4**	RPL23	75,79457	1,3865393	29
**T cells CD4**	RPL14	75,076485	1,5354918	30
**T cells CD4**	RPS8	74,75402	1,5333148	31
**T cells CD4**	RPL41	74,73467	1,2969648	32
**T cells CD4**	RPS13	73,51074	1,466338	33
**T cells CD4**	TRBC2	73,1803	2,3182786	34
**T cells CD4**	RPLP1	73,13855	1,6327522	35
**T cells CD4**	RPS3	73,02755	1,5382305	36
**T cells CD4**	RPL3	72,463104	1,5071645	37
**T cells CD4**	RPS3A	72,20832	1,4530028	38
**T cells CD4**	CD69	72,16426	2,3422308	39
**T cells CD4**	RPL4	71,971	1,5264373	40
**T cells CD4**	RPL27A	71,958305	1,4149897	41
**T cells CD4**	RPS23	71,81256	1,4803815	42
**T cells CD4**	RPS26	71,58119	1,4752681	43
**T cells CD4**	RPL12	71,02191	1,4686738	44
**T cells CD4**	RPLP2	71,01111	1,5700933	45
**T cells CD4**	RPL37A	70,9273	1,42204	46
**T cells CD4**	RPL37	70,299515	1,3314172	47
**T cells CD4**	RPL10A	69,710594	1,4943309	48
**T cells CD4**	RPL5	69,51131	1,4453275	49
**T cells CD4**	ZFP36L2	68,87769	1,7412412	50
**T cells CD4**	RPL35A	68,7411	1,3859	51
**T cells CD4**	RPS21	68,57947	1,4714143	52
**T cells CD4**	RPL23A	68,330055	1,4433976	53
**T cells CD4**	CXCR4	68,25825	1,8786546	54

**T cells CD4**	CD52	68,21042	2,1668959	55
**T cells CD4**	RPS15A	67,86951	1,4589179	56
**T cells CD4**	SARAF	67,16337	1,3813232	57
**T cells CD4**	RPL27	64,83741	1,3014992	58
**T cells CD4**	RPL10	64,19179	1,3837575	59
**T cells CD4**	MT-ND2	63,95995	0,8233607	60
**T cells CD4**	RPL6	63,943607	1,308492	61
**T cells CD4**	RPL36	63,388176	1,4753091	62
**T cells CD4**	ETS1	62,956238	1,8316959	63
**T cells CD4**	RPL19	62,502148	1,3757699	64
**T cells CD4**	CD3D	62,137184	2,2617633	65
**T cells CD4**	CD40LG	61,742428	4,3575478	66
**T cells CD4**	PABPC1	60,39317	1,0794678	67
**T cells CD4**	CD4	59,79692	2,400094	68
**T cells CD4**	CD3G	59,431942	2,250643	69
**T cells CD4**	RPS2	59,39733	1,3003263	70
**T cells CD4**	GPR171	59,392838	2,2619576	71
**T cells CD4**	RPS19	58,806564	1,4325031	72
**T cells CD4**	BCL11B	58,708755	2,3158693	73
**T cells CD4**	RPS11	58,09666	1,2309854	74
**T cells CD4**	RCAN3	57,98237	2,6509676	75
**T cells CD4**	RPSA	55,494965	1,3615105	76
**T cells CD4**	RPS5	54,329567	1,2768757	77
**T cells CD4**	ITK	54,228638	2,1642773	78
**T cells CD4**	LEPROTL1	53,732212	1,6353508	79
**T cells CD4**	IL32	53,66358	1,7397805	80
**T cells CD4**	CD96	52,381622	1,6322156	81
**T cells CD4**	GPR183	52,18867	2,3529427	82
**T cells CD4**	AHNAK	52,09031	1,4676012	83
**T cells CD4**	DGKA	51,86578	2,1153855	84
**T cells CD4**	RPS24	51,350727	1,0734845	85
**T cells CD4**	TCF7	50,89319	2,4774191	86
**T cells CD4**	MAF	50,858047	1,9846268	87
**T cells CD4**	BCL2	50,810383	2,0293505	88
**T cells CD4**	RPL26	50,33558	1,191795	89
**T cells CD4**	TOMM7	50,08268	1,1717743	90
**T cells CD4**	RPS7	49,966763	1,087611	91
**T cells CD4**	SYNE2	48,913437	1,5203124	92
**T cells CD4**	NPM1	48,844322	1,1162388	93
**T cells CD4**	JUN	48,63963	1,5326619	94
**T cells CD4**	EEF1B2	48,31422	1,1998645	95
**T cells CD4**	RPL24	47,954704	1,0893807	96
**T cells CD4**	LTB	47,845963	1,901362	97
**T cells CD4**	TNFAIP3	47,474037	1,4251624	98

**T cells CD4**	RACK1	47,005318	1,0390844	99
**T cells CD4**	RPL29	46,91386	1,1365274	100
**T cells CD8**	CD3E	141,21815	3,6680644	1
**T cells CD8**	CCL5	130,22484	3,593617	2
**T cells CD8**	TRAC	129,32382	3,5900023	3
**T cells CD8**	TRBC2	126,27603	3,2885687	4
**T cells CD8**	CD8A	117,467865	5,831979	5
**T cells CD8**	CD3D	114,77486	3,7185738	6
**T cells CD8**	RPS29	113,77661	1,7949412	7
**T cells CD8**	IL32	113,43218	2,943158	8
**T cells CD8**	GZMA	112,61171	2,9667659	9
**T cells CD8**	CD2	108,269394	2,8575706	10
**T cells CD8**	GZMK	107,46986	3,4141202	11
**T cells CD8**	CD3G	107,42869	3,6139197	12
**T cells CD8**	RPS27	107,1289	1,7891673	13
**T cells CD8**	RPS27A	105,02162	1,6975635	14
**T cells CD8**	RPL34	103,7018	1,6104871	15
**T cells CD8**	RPS12	100,189224	1,7361895	16
**T cells CD8**	TRGC2	98,82774	3,6176054	17
**T cells CD8**	SPOCK2	97,83296	2,7261224	18
**T cells CD8**	CD69	96,60507	2,492346	19
**T cells CD8**	RPL27A	93,04474	1,4947	20
**T cells CD8**	RPL21	91,44655	1,4794413	21
**T cells CD8**	GPR171	90,887665	2,910297	22
**T cells CD8**	MT-ND5	90,37146	1,1889977	23
**T cells CD8**	RPS3	90,03183	1,5202296	24
**T cells CD8**	RPS20	89,36751	1,4770552	25
**T cells CD8**	CD96	89,185265	2,238649	26
**T cells CD8**	HLA-A	88,32606	0,914001	27
**T cells CD8**	RUNX3	86,95056	2,0344882	28
**T cells CD8**	RPL31	86,79028	1,4374746	29
**T cells CD8**	FYN	86,08028	1,9728079	30
**T cells CD8**	IKZF3	84,4081	2,110803	31
**T cells CD8**	RPL13A	83,18934	1,4314743	32
**T cells CD8**	ETS1	81,63301	1,9202718	33
**T cells CD8**	RPL39	81,02973	1,2738112	34
**T cells CD8**	PRF1	80,71581	2,3492727	35
**T cells CD8**	SLFN12L	80,61492	2,1573825	36
**T cells CD8**	KCNA3	80,06075	2,9187076	37
**T cells CD8**	ARL4C	79,92298	1,9467403	38
**T cells CD8**	RPL23A	79,52111	1,3617582	39
**T cells CD8**	EEF1A1	78,72968	1,4577528	40
**T cells CD8**	RPS6	78,35337	1,3938609	41
**T cells CD8**	GIMAP7	78,25565	1,8922819	42

**T cells CD8**	SLC38A1	76,28876	1,734438	43
**T cells CD8**	RPL30	74,92714	1,2024292	44
**T cells CD8**	KLRG1	74,60717	3,5135663	45
**T cells CD8**	HLA-B	74,24116	0,7622877	46
**T cells CD8**	RPS26	74,09272	1,2401336	47
**T cells CD8**	MT-ND2	73,70747	0,79965484	48
**T cells CD8**	PDCD4	73,54633	1,6043665	49
**T cells CD8**	RPL41	72,58574	1,0750761	50
**T cells CD8**	RPL32	72,45662	1,281443	51
**T cells CD8**	PPP2R5C	72,41072	1,3910142	52
**T cells CD8**	RPS23	71,87075	1,2692252	53
**T cells CD8**	RPL9	71,71871	1,2109265	54
**T cells CD8**	RPL35A	71,61683	1,1948924	55
**T cells CD8**	CCR5	71,549065	3,013702	56
**T cells CD8**	CCL4	71,12727	1,8827024	57
**T cells CD8**	CDC42SE2	69,90233	1,4877582	58
**T cells CD8**	BCL11B	69,846695	2,4182725	59
**T cells CD8**	RPL37A	69,7044	1,2295984	60
**T cells CD8**	LINC01871	69,422554	2,9427156	61
**T cells CD8**	RPS4X	69,31812	1,2454492	62
**T cells CD8**	AHNAK	68,51084	1,5517476	63
**T cells CD8**	RPL23	68,274574	1,1320829	64
**T cells CD8**	TMSB4X	67,30749	0,5842746	65
**T cells CD8**	SLAMF7	66,6729	1,9046714	66
**T cells CD8**	CXCR6	66,47819	2,6125565	67
**T cells CD8**	PTPRC	66,34934	1,0449356	68
**T cells CD8**	GBP5	66,19032	1,86116	69
**T cells CD8**	NAP1L1	66,08082	1,2078365	70
**T cells CD8**	RPS21	65,871284	1,155916	71
**T cells CD8**	RPS15A	65,65029	1,1448252	72
**T cells CD8**	LCK	65,523186	2,237021	73
**T cells CD8**	GPRIN3	65,46791	1,754307	74
**T cells CD8**	RPS16	65,43762	1,0731642	75
**T cells CD8**	RPL6	65,375534	1,1485577	76
**T cells CD8**	IL7R	65,01344	2,2633805	77
**T cells CD8**	RBL2	64,78499	1,4751275	78
**T cells CD8**	CD52	64,661606	1,6844751	79
**T cells CD8**	RPL11	64,58922	1,1143426	80
**T cells CD8**	RPS25	64,04751	1,0810825	81
**T cells CD8**	RPL13	63,956497	1,1265628	82
**T cells CD8**	RPL3	63,53629	1,1256044	83
**T cells CD8**	MT-ND4L	63,145382	0,9261577	84
**T cells CD8**	RPS13	63,063396	1,139823	85
**T cells CD8**	LBH	62,803894	1,7553833	86

**T cells CD8**	RASGRP1	62,722965	2,0969958	87
**T cells CD8**	CD48	62,720745	1,3562086	88
**T cells CD8**	RPL38	62,637157	1,0500447	89
**T cells CD8**	TC2N	61,753407	2,0786152	90
**T cells CD8**	CLEC2D	61,608322	2,0233793	91
**T cells CD8**	B2M	61,520626	0,50543064	92
**T cells CD8**	RPS3A	60,771244	1,0476166	93
**T cells CD8**	PTP4A2	60,504818	0,92892355	94
**T cells CD8**	IL2RG	60,450554	1,1667757	95
**T cells CD8**	RPL14	60,42785	1,0612227	96
**T cells CD8**	RPLP2	59,82398	1,0781524	97
**T cells CD8**	MBNL1	59,791767	0,95393133	98
**T cells CD8**	PTMA	59,573006	1,1239861	99
**T cells CD8**	ZFP36L2	59,52245	1,182327	100

Table S3. Cell Type Composition of scRNA-seq data

Cell Numbers of each Sample after QC

Liver	Timepoint T0	Timepoint T1	Total
Liver 1	8749	6440	15189
Liver 2	13472	10074	23546
Liver 3	4257	1683	5940
Liver 4	9374	4538	13912
Liver 5	5724	5402	11126
Liver 6	3471	1920	5391
Total	45047	30057	75104

Cell Numbers of Cell Types in each Liver (T0, T1; after QC)

(% of total cells)	P1	P2	P3	P4	P5	P6	All
**B cells**	1,84	2,2	1,82	1,28	3,09	0,24	1,92
**CRC cells**	0,52	0,79	18,97	1,45	18,86	6,64	5,39
**Cholangiocytes**	0,03	0,11	0,12	0,22	0,3	7,83	0,7
**Endothelial cells**	2,78	0,71	0,77	1,88	0,67	0,95	1,36
**Fibroblasts**	0,13	0,38	1,53	0,37	0,45	0,37	0,43
**Hepatocytes**	0,32	0,18	0,32	0,56	0,12	0,15	0,28
**Mast cells**	0,06	0,16	0,02	0,16	0,14	0,17	0,13
**Monocytes/Macrophages**	6,13	15,66	36,52	19,51	5,37	18,98	14,81
**NK cells**	37,88	7,35	5,72	21,36	7,31	14,17	16,47
**Neutrophil Progenitors**	0,52	0,06	1,45	0,88	0,04	0,61	0,45
**Neutrophils**	7,98	23,08	17,61	30,13	25,18	32,35	21,88
**Plasma cells**	0,34	0,38	0,62	0,33	1,97	0,22	0,6
**T cells CD4**	15,45	10,06	5,77	8,5	20,01	6,88	11,77
**T cells CD8**	23,12	37,02	7,42	11,32	15,85	8,94	21,95
**cDCs**	2,77	1,71	1,21	1,55	0,53	1,48	1,66
**pDCs**	0,14	0,14	0,12	0,52	0,11	0,02	0,19

absolute numbers	T0	T1
**B cells**	1006	434
**CRC cells**	2930	1121
**Cholangiocytes**	459	65
**Endothelial cells**	775	250
**Fibroblasts**	125	196
**Hepatocytes**	106	103
**Mast cells**	37	58
**Monocytes/Macrophages**	6266	4856
**NK cells**	5713	6658
**Neutrophil Progenitors**	218	121
**Neutrophils**	15056	1374
**Plasma cells**	249	205
**T cells CD4**	4485	4354
**T cells CD8**	6549	9940
**cDCs**	936	313
**pDCs**	137	9
**Total**	45047	30057

absolute numbers	P1	P2	P3	P4	P5	P6	All
**B cells**	280	517	108	178	344	13	1440
**CRC cells**	79	187	1127	202	2098	358	4051
**Cholangiocytes**	5	27	7	30	33	422	524
**Endothelial cells**	423	168	46	262	75	51	1025
**Fibroblasts**	19	90	91	51	50	20	321
**Hepatocytes**	48	43	19	78	13	8	209
**Mast cells**	9	38	1	22	16	9	95
**Monocytes/Macrophages**	931	3687	2169	2714	598	1023	11122
**NK cells**	5753	1730	340	2971	813	764	12371
**Neutrophil Progenitors**	79	14	86	122	5	33	339
**Neutrophils**	1212	5435	1046	4191	2802	1744	16430
**Plasma cells**	51	89	37	46	219	12	454
**T cells CD4**	2347	2369	343	1183	2226	371	8839
**T cells CD8**	3512	8716	441	1575	1763	482	16489
**cDCs**	420	403	72	215	59	80	1249
**pDCs**	21	33	7	72	12	1	146
**Total**	15189	23546	5940	13912	11126	5391	75104

Cell Numbers of Cell Types per material in each Liver before (T0) and at the end of NMP (T1) (after QC)

patient and timepoint		P1 T0	P1 T1	P2 T0	P2 T1	P3 T0	P3 T1	P4 T0	P4 T1	P5 T0	P5 T1	P6 T0	P6 T1	Total T0	Total T1
cell_type	material														
B cells	CRLM	36	35	298	47	13	32	48	15	268	34	4	2	667	165
B cells	adj.liver	132	77	92	80	55	8	41	74	17	25	2	5	339	269
CRC cells	CRLM	46	15	142	39	1070	21	173	13	1109	747	289	60	2829	895
CRC cells	adj.liver	15	3	3	3	36	0	8	8	30	212	9	0	101	226
Cholangiocytes	CRLM	1	2	18	2	2	1	20	6	0	3	401	7	442	21
Cholangiocytes	adj.liver	0	2	4	3	4	0	0	4	0	30	9	5	17	44
Endothelial cells	CRLM	61	11	57	36	5	5	147	27	45	2	23	6	338	87
Endothelial cells	adj.liver	292	59	24	51	21	15	60	28	21	7	19	3	437	163
Fibroblasts	CRLM	13	0	11	67	1	49	33	1	30	14	16	3	104	134
Fibroblasts	adj.liver	4	2	1	11	0	41	14	3	1	5	1	0	21	62
Hepatocytes	CRLM	10	7	13	9	5	13	56	8	0	3	2	0	86	40
Hepatocytes	adj.liver	6	25	1	20	0	1	9	5	1	9	3	3	20	63
Mast cells	CRLM	5	0	6	12	0	1	11	2	3	11	8	0	33	26
Mast cells	adj.liver	2	2	0	20	0	0	2	7	0	2	0	1	4	32
Monocytes/Macrophages	CRLM	233	96	1264	482	638	177	716	344	140	141	259	184	3250	1424
Monocytes/Macrophages	adj.liver	331	271	979	962	680	674	428	1226	183	134	415	165	3016	3432
NK cells	CRLM	203	405	486	107	22	90	728	120	23	42	39	36	1501	800
NK cells	adj.liver	2676	2469	344	793	182	46	768	1355	139	609	103	586	4212	5858
Neutrophil Progenitors	CRLM	4	4	4	1	2	1	48	1	0	0	0	5	58	12
Neutrophil Progenitors	adj.liver	20	51	7	2	77	6	43	30	3	2	10	18	160	109
Neutrophils	CRLM	71	46	2028	40	390	67	2022	112	514	119	268	73	5293	457
Neutrophils	adj.liver	941	154	3204	163	493	96	1903	154	1967	202	1255	148	9763	917
Plasma cells	CRLM	18	9	12	20	1	2	21	5	128	60	2	0	182	96
Plasma cells	adj.liver	13	11	5	52	27	7	11	9	9	22	2	8	67	109
T cells CD4	CRLM	909	544	1155	440	68	98	804	85	550	752	91	101	3577	2020
T cells CD4	adj.liver	469	425	118	656	142	35	94	200	52	872	33	146	908	2334
T cells CD8	CRLM	554	532	1875	1260	70	126	772	107	332	437	76	49	3679	2511
T cells CD8	adj.liver	1285	1141	993	4588	202	43	200	496	133	861	57	300	2870	7429
cDCs	CRLM	210	18	232	24	23	15	102	21	13	18	52	3	632	99
cDCs	adj.liver	168	24	63	84	21	13	21	71	8	20	23	2	304	214
pDCs	CRLM	6	0	18	0	0	0	48	0	4	1	0	1	76	2
pDCs	adj.liver	15	0	15	0	7	0	23	1	1	6	0	0	61	7

Table S4. Percentage of DEG per cell type and tissue at T0

Cell Type	Differentially Expressed Genes	Total Genes	Percentage
Monocytes-Macrophages	1558	24765	6,291136685
NK_cells	53	18879	0,280735208
Neutrophils	186	23384	0,795415669
T_cells_CD4	0	18250	0
T_cells_CD8	58	19818	0,292663235
cDCs	89	15505	0,574008384
B_cells	2	13135	0,015226494
Endothelial_cells	244	14143	1,725235099

Table S5. Tumor-associated myeloid cell resistance signature

LN3 - top genes	LM3 Marker - top genes	TMRS (LN3 & LM3 intersection)
CXCL8	GPNMB	ABCA1
VEGFA	OLR1	ABCG1
C15orf48	SPP1	C15orf48
PKM	CD9	CD83
IER3	RGS1	CSTB
CCRL2	VEGFA	CXCR4
SQSTM1	APOC1	JUN
CYBB	ABCA1	OLR1
NFKBIA	BTG1	P4HA1
CCL3L3	P4HA1	PLIN2
BHLHE40	TNS1	SPP1
CD83	CXCR4	VEGFA
CCR5AS	C15orf48	
CANX	PLIN2	
JUN	BNIP3L	
CXCR4	SLC16A10	
PPIF	RGCC	
P4HA1	ERO1A	
CDKN1A	ABCG1	
SPP1	CD109	
ENO1	SCD	
TPI1	HK2	
CCL3	ALOX5AP	
APLP2	A2M	
OLR1	ATF3	
CCL4L2	BCAT1	
TNFAIP3	CD83	
ICAM1	MXI1	
IER5	ELL2	
TPP1	SDS	
IRAK2	CXCL16	
LIMS1	FN1	
FTH1	LDHA	
CSTB	SLC2A5	
FNDC3B	SDC2	
PLIN2	LGMN	
CCL4	FCGR2B	
PTMA	BNIP3	
CD44	RNASE1	
M6PR	SLC2A3	
IRF2BP2	CSTB	
DSE	ADAM8	
C3AR1	RALA	
PHACTR1	VIM	
TGM2	JUN	
LGALS3	SLC2A1	
CD63	ACP5	
ABCA1	EGLN3	
NPC2	GBE1	
ABCG1	COLEC12	

Table S6. Antibody list for flow-cytometry

Marker	Clone	Fluorochrome	Dilution	Supplier
CD34	HPCA-2	FITC	1:20	BD Biosciences
7AAD	-	BB700	1:14	BD Biosciences
CD90	5E10	APC	1:100	Biolegend
CD19	HI98	APC-R700	1:400	BD Biosciences
CD16	eBioCB16	APC-eF780	1:25	Thermo
CD56	NCAM16.2	BUV395	1:100	BD Biosciences
CD3	UCHT	BUV496	1:400	BD Biosciences
CD8	RPA-T8	BUV563	1:200	BD Biosciences
CD28	CD28.2	BUV615P	1:500	BD Biosciences
CD4	SK3	BUV737	1:200	BD Biosciences
CD45	HI30	BUV805	1:100	BD Biosciences
CD38	HIT2	BV421	1:200	BD Biosciences
HLA-DR	G46-6	BV480	1:100	BD Biosciences
CD31	WM59	BV605	1:100	Biolegend
CD123	7G3	BV650	1:200	BD Biosciences
CD14	MφP9	BV711	1:200	BD Biosciences
CD15	HI98	BV786	1:100	BD Biosciences
CD161	DX12	PE	1:40	BD Biosciences
CD193	5E8	PE-CF594	1:167	BD Biosciences
CD326	9C4	PE-Cy7	1:2000	Biolegend

Table S7. Overview of antibodies used for multispectral imaging

Antibody	Clone	Provider	Dilution	pH (AR)	Opal Pairing
**Panel 1. Immunopanel**					
CD20	L26	Dako	1:200	6	540
CD8	C8/144B	Dako	1:200	9	570
CD3	polyclonal	Dako	1:250	6	620
CD68	PG-M1	Dako	1:200	9	650
Cytokeratin	AE1/AE3<br>C-11	Dako<br>Abcam	1:500<br>1:1000	9	690
DAPI	-	Akoya Biosciences	1:15	-	450
**Panel 2. Vimentin – Cytokeratin**					
Vimentin	EPR3776	Abcam	1:600	9	620
Cytokeratin	AE1/AE3<br>C-11	Dako<br>Abcam	1:500<br>1:1000	9	690
DAPI	-	Akoya Biosciences	1:15	-	450
