# Supplementary material for: Ex vivo modelling of human colorectal cancer liver metastasis by normothermic machine perfusion
Source: Mol Cancer. 2025 Oct 21;24:264. doi: 10.1186/s12943-025-02430-7 (PMC12542284; doi:10.1186/s12943-025-02430-7)
Supplement: Supplementary file 2 — Supplementary Material 2. [file 12943_2025_2430_MOESM2_ESM.pdf]

Ex vivo modelling of human colorectal cancer liver metastasis by normothermic machine perfusion

Manuel Trebo[1,†], Thomas Maurer[1,†], Felix J. Krendl[2,†], Stefan Salcher[1,†], Agnieszka Martowicz[1,3], Theresa Hautz[2], Sieghart Sopper[1], Arno Amann[1], Benno Cardini[2], Lukas H. Poelsler[2], Anna Mair[1], Julia Hofmann[2], Andras T. Meszaros[2], Martin Hermann[2], Michael Günther[4,5], Steffen Ormanns[4,5], Zlatko Trajanoski[6], Stefan Schneeberger[2,‡], Dominik Wolf[1,‡], Rupert Oberhuber[2,‡,*], Andreas Pircher[1,‡,*]

* Corresponding authors

† Contributed equally as first authors

‡ Contributed equally as senior authors

Affiliations

[1] Department of Internal Medicine V, Hematology and Oncology, Comprehensive Cancer Center Innsbruck (CCCI), Austrian Comprehensive Cancer Network (ACCN) and Tyrolean Cancer Research Institute (TKFI), Medical University of Innsbruck, Innsbruck, Austria

[2] Department of Visceral, Transplant and Thoracic Surgery, Center of Operative Medicine, organLife Laboratory and D. Swarovski Research Laboratory, Medical University of Innsbruck, Innsbruck, Austria

[3] Tyrolpath Obrist Brunhuber GmbH, Zams, Austria

[4] Innpath Institute of Pathology, Tirol Kliniken, Innsbruck, Austria

[5] Institute of General Pathology, Medical University Innsbruck, Innsbruck, Austria

[6] Biocenter, Institute of Bioinformatics, Medical University of Innsbruck, Innsbruck, Austria

Correspondence

* Dr. Andreas Pircher PhD, Department of Internal Medicine V, Hematology and Oncology, and Tyrolean Cancer Research Institute (TKFI), Anichstr. 35, A-6020 Innsbruck, Austria, Electronic address: andreas.pircher@i-med.ac.at

* Dr. Rupert Oberhuber PhD, Department of Visceral, Transplant and Thoracic Surgery, Center for Operative Medicine, Medical University of Innsbruck, Austria.

Electronic address: rupert.oberhuber@i-med.ac.at

Figure Overview:

Figure S1. Surgical preparation and perfusion dynamics of livers with metastatic lesions 8

Figure S2. Perfusion parameters of long-term perfused patient 7 9

Figure S3. Cellular composition of the *ex vivo* CRLM model 10

Figure S4. Metastatic heterogeneity of cancer cells before perfusion start (T0) 11

Figure S5. Spatial representation of Progeny activities and pathologist annotations 12

Figure S6. Compositional impact of NMP on cell types in CRLM and adjacent liver tissue ... 13

Figure S7. Tissue and TME integrity analysis throughout perfusion 14

Figure S8. Impact of NMP on cellular stress 15

Figure S9. Detailed phenotyping of T- and NK cells stability during NMP 16

Figure S10. Impact of NMP on pathway activities within tissues 17

Figure S11. Impact of NMP on monocytes/macrophages and neutrophils 18

Table Overview:

Supplementary Table S1. Patient characteristics CRC NMP (OrganOx® metra®).

Supplementary Table S2. Top ranked genes per cell type cluster

Supplementary Table S3. Cell Type Composition of scRNA-seq data

Supplementary Table S4. Percentage of DEG per cell type and tissue at T0

Supplementary Table S5. Tumor-associated myeloid cell resistance signature

Supplementary Table S6. Antibody list for flow-cytometry

Supplementary Table S7. Overview of antibodies used for multispectral imaging

Supplementary figure legends:

Figure S1. Surgical preparation and perfusion dynamics of livers with metastatic lesions

A) and B) Surgical Reconstruction after organ explantation. C and D) Outflow reconstruction and reconstruction of the portal vein – in anticipation of cannulation for NMP – was performed using a pericardium patch. The hepatic artery and bile ducts were cannulated directly. E) Biopsies were taken ultrasound-guided to guarantee reliable tumor sampling and F) the liver specimen was then transferred to the OrganOx® Metra® and NMP commenced. G) Metabolic and hepatic parameters were monitored over the course of perfusion, including lactate, pH, glucose, ALT, and AST. Line graphs show individual perfusion profiles (patient 1–6) over perfusion time.

Figure S2. Perfusion dynamics of long-term perfusion.

A) Flow and pressure curves, as well as bile flow of patient 7 during 168 hours of NMP are shown. B) Metabolic and hepatic parameters were evaluated over the course of long-term perfusion of patient 7, including lactate, pH, ALT, and AST. C) H&E staining of adjacent liver (top) and CRLM tissue (bottom) are depicted after 168 hours of NMP.

Figure S3. Cellular composition of the *ex vivo* CRLM model

A) UMAP reduction showing canonical CRC marker expression on the complete scRNA-seq dataset and B) color-coding by individual patients of both tissues (CRLM and adjacent liver) and both timepoints (T0 and T1). C) Barplots showing cell type percentages without mCRC cells in scRNA-seq data per patient and tissue (CRLM – top or adjacent liver – bottom) including both timepoints. D) Cell2Location deconvoluted images of CRLM (left) and adjacent liver tissues (right) at both timepoints (T0 and T1) showing the predicted most dominant cell type per spot for patient 1.

Figure S4. Metastatic heterogeneity of cancer cells before perfusion start (T0)

A) UMAP visualization of cells clustered in CNV space color-coded by the predicted CNV score (left), patient identity (middle) or cell-type at T0 (right). B) UMAP plot showing expression levels of iCMS2 (top) or iCMS3 (bottom) on subclustered mCRC cells of a publicly available CRC atlas. C) UMAP and D) dotplot showing metastatic differentiation signature expression of subclustered mCRC cells at T0. E) Spatial representation of metastatic signatures in CRLM tissue of patient 3 and 5 at T0. F) Patient (left) or leiden-cluster (right) color-coded proportional composition of mCRC cells at T0.

Figure S5. Spatial representation of Progeny activities and pathologist annotations

A) Selected spatial ProgenY pathway activities (Hypoxia, TGF-β and NF-κB) within adjacent liver tissues of patients at T0. B) Spatial image overview of adjacent liver (left) and CRLM (right) tissues at T0 showing all selected region of interests (ROIs) with H&E pathologist-based annotation.

Figure S6. Compositional impact of NMP on cell types in CRLM and adjacent liver tissue

A) UMAP color-coded by timepoint pre NMP (T0) and after NMP (T1) in adjacent liver tissue. B) Relative cell type composition in adjacent liver tissues pre (T0) and after (T1) NMP. C) Cell-type proportional change deconvoluted per patient in CRLM tissue (left) or adjacent liver tissue (right). D) scCODA calculated proportional shifts between timepoints (T0 vs T1) of CRLM (top) and adjacent liver tissues. E) Sequential perfusate analysis by routine flow-cytometry of leukocytes and major lymphocyte populations ($CD4^+$-, $CD8^+$ T cells, NK cells and B cells) are shown as total cell counts per µl in long-term perfused patient 7.

Figure S7. Tissue and TME integrity analysis throughout perfusion

A) H&E stainings and multiplex IF images of immunology markers presented before (T0) and after (T1) NMP applied to corresponding adjacent liver and CRLM tissue specimen. Legend

depicts color-coding of fluorescence; images are displayed at ×20 magnification (scale bar: 100 μm).

Figure S8. Impact of NMP on cellular stress

A) Quality parameters and abundance of mitochondrial reads calculated as mean per patient and tissue (CRLM – top or adjacent liver – bottom) before (T0) and after NMP (T1). B) Stress-scores in adjacent liver tissue calculated as mean per cell-type and patient between pre (T0) and after (T1) NMP. C) Representative spatial image of stress-scores in patient 3 in CRLM (top) or adjacent liver (bottom) tissue before (T0 – left) and after NMP (T1 – right). D) Stress-scores of spatial images calculated as mean per patient and spot between tissues (CRLM – top, adjacent liver – bottom).

Figure S9. Detailed phenotyping of T- and NK cell stability during NMP

A) Marker gene expression of top regulated markers T- and NK cell-specific genes in individual cell types B) UMAP illustration highlighting $CD4^{+}$, $CD8^{+}$ T cells and NK cells. C) UMAP plots showing NK- and T cells color-coded by cell type subset (top left), material (top right), timepoint (bottom left) and patient (bottom right). D) Barplots (top) per dataset and boxplots per patient illustrating timepoint dynamics of T- and NK cell subsets. E) UMAP (left) and Violin Plots (right) depicting cytotoxicity (top) and exhaustion (bottom) scores per cell and timepoint (T0 vs T1). F) Gene expression of key checkpoint markers in CRLM tissue by patient (mean) and timepoint (T0 vs T1).

Figure S10. Impact of NMP on pathway activities within tissues

A) Spatial image overview of adjacent liver (left) and CRLM (right) tissues at T1 showing all selected region of interests (ROIs) used for quantification based on H&E pathologist annotation. B) Selected ProgenY pathway activities (Hypoxia, NF-κB and TGF-β) within both CRLM (top panel) and adjacent liver tissue (bottom panel) of patients at T1. C) Mean pathway activities per ROI of tissue types (divided into adjacent liver, fibrotic and tumor regions)

compared between timepoints (T0 vs T1). D) Comparison of selected cell proliferation associated genes between timepoints in CRLM (T0 vs T1).

Figure S11. Impact of NMP on monocytes/macrophages and neutrophils

A) Comparison of logFC from DEG between tissues (CRLM vs. adjacent liver) before (T0) and after NMP (T1) for monocytes/macrophages (left) and neutrophils (right), color-coded as follows: non-significant or significant at only one timepoint (white), significantly upregulated (red) or downregulated (blue) at both timepoints, and differentially regulated in opposite directions between timepoints (green). B) Relative percentages of individual Leiden clusters at T0, separated by tissue type (adjacent liver and CRLM), for monocytes/macrophages (top) and neutrophils (bottom). C) UMAP visualization showing subclustered monocytes/macrophage (top) and neutrophils (bottom) color-coded by timepoint before (T0) and after (T1) NMP. D) Compositional shift of monocyte/macrophage (top panel) and neutrophil (bottom panel) subcluster fractions between tissues (adjacent liver: top; CRLM: bottom) within individual patients, before (T0) and after (T1) NMP.

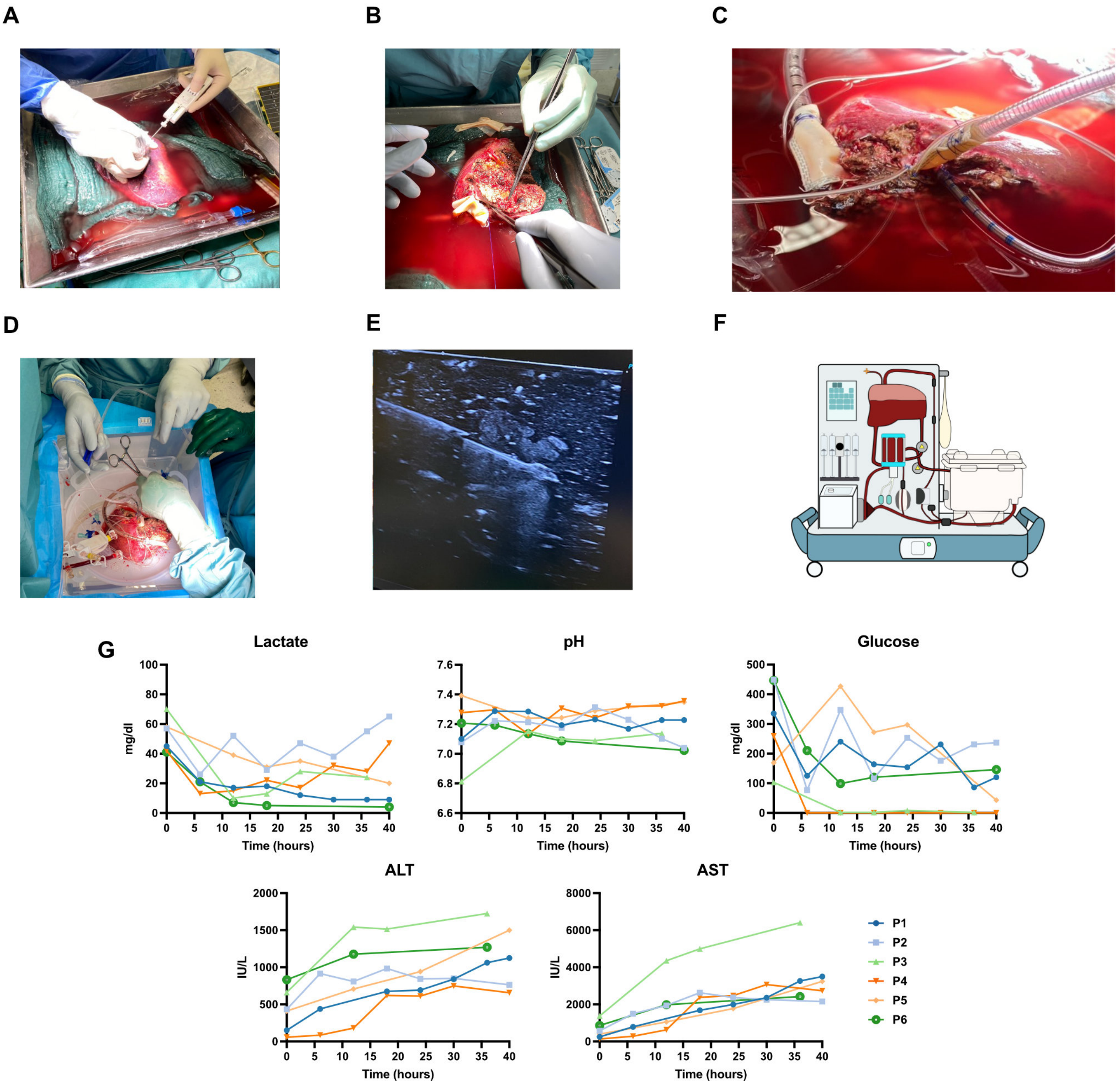

Figure S1. Surgical preparation and perfusion dynamics of livers with metastatic lesions

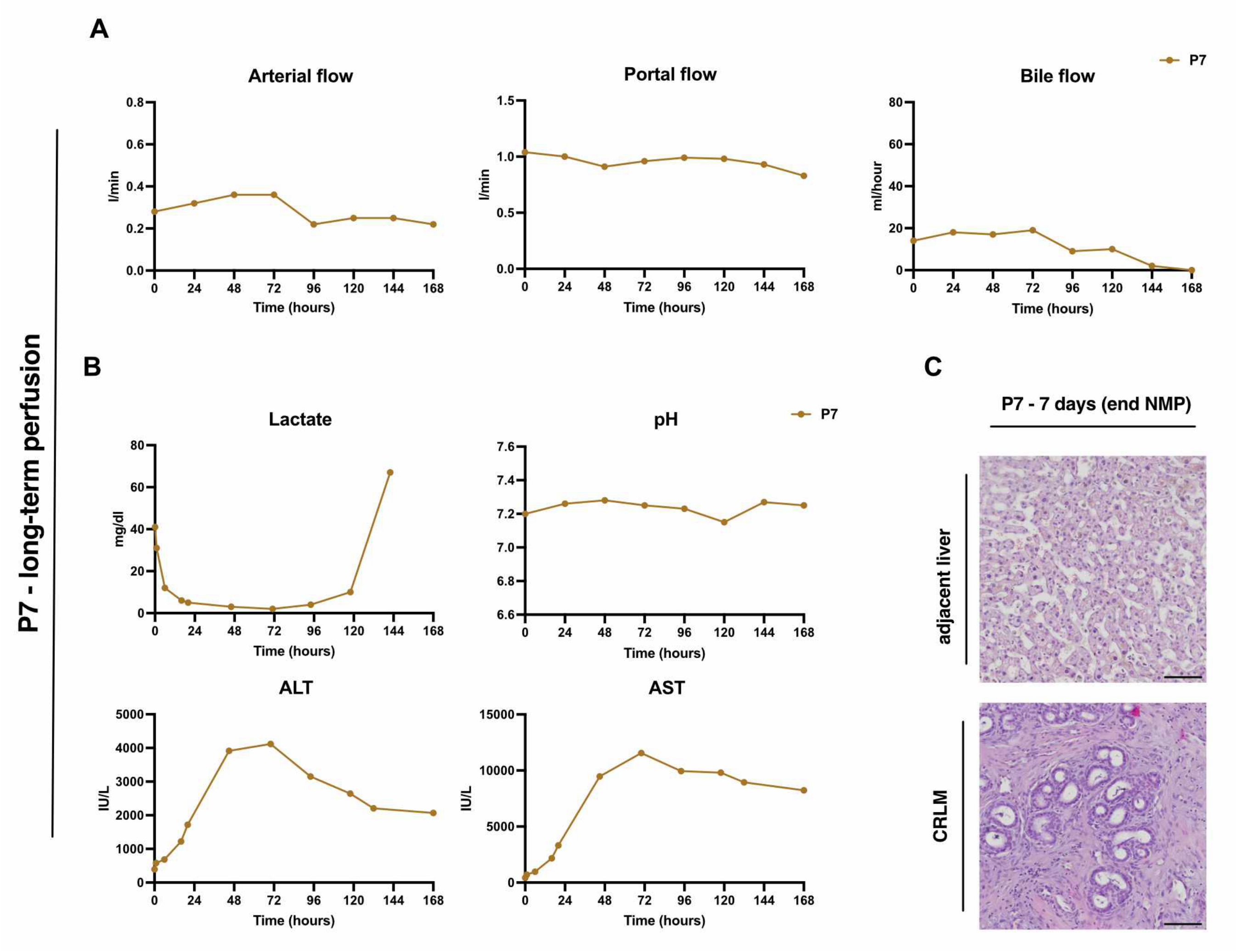

Figure S2. Perfusion parameters of long-term perfused patient 7

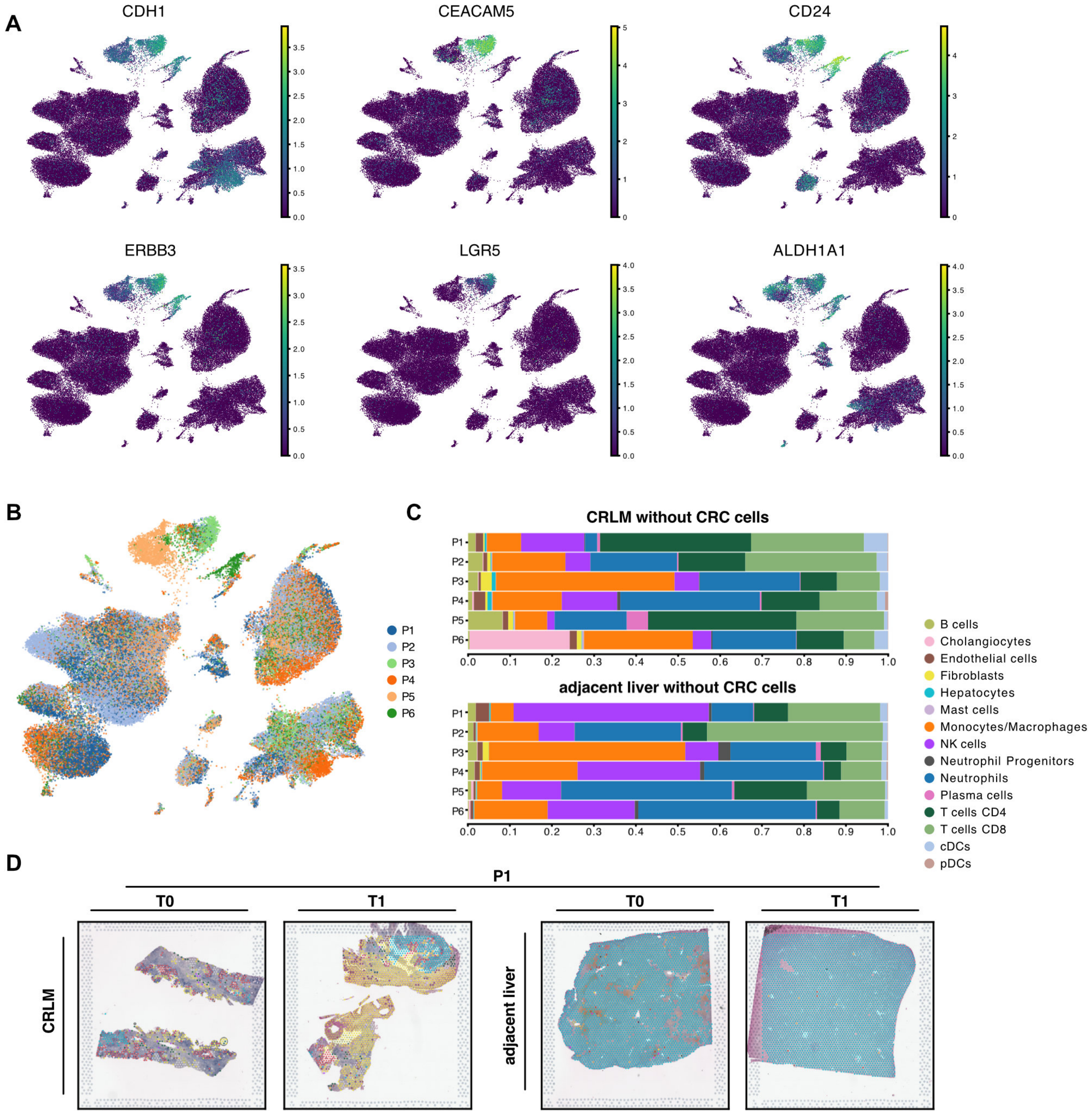

Figure S3. Cellular composition of the *ex vivo* CRLM model

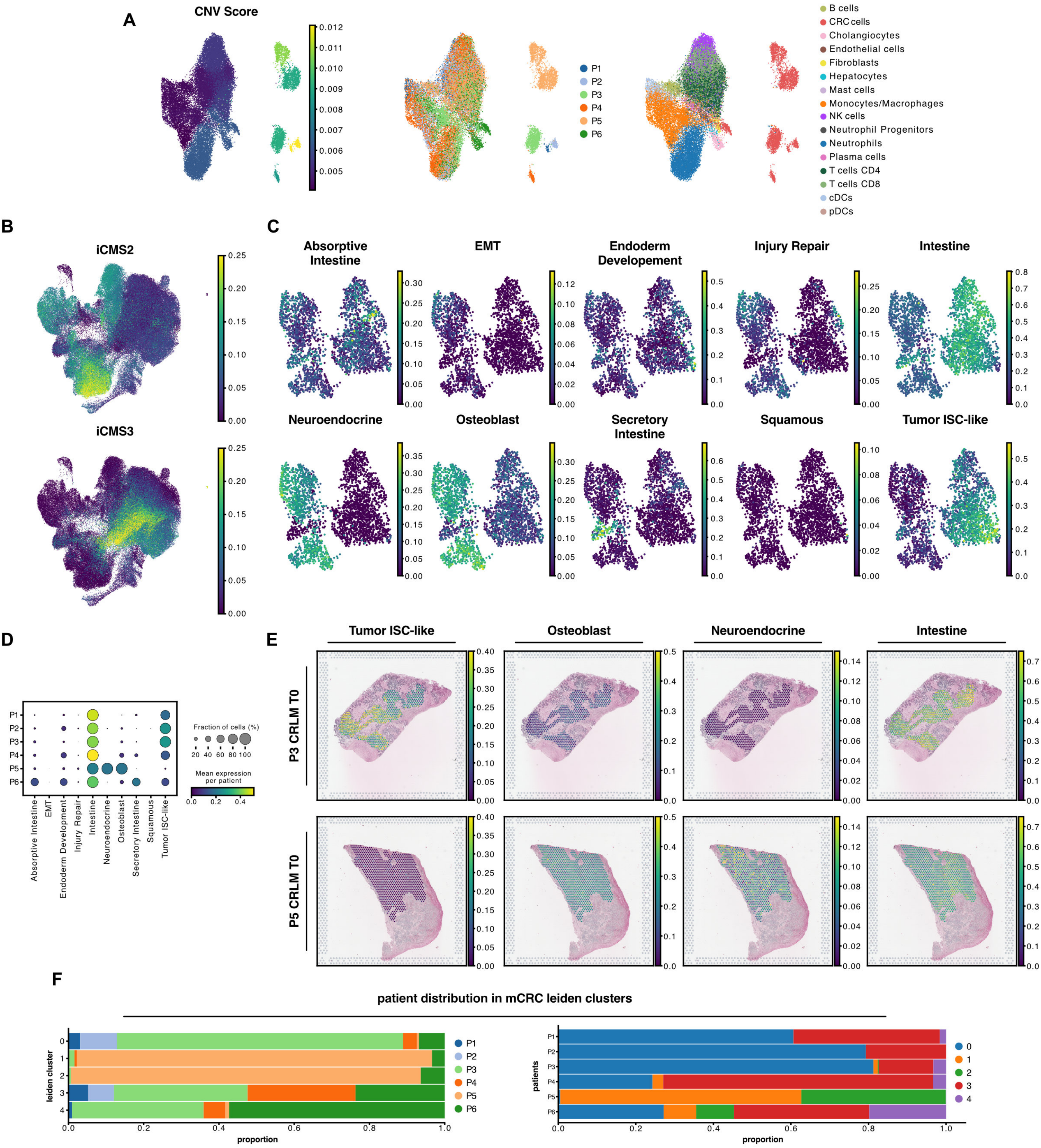

Figure S4. Metastatic heterogeneity of cancer cells before perfusion start (T0)

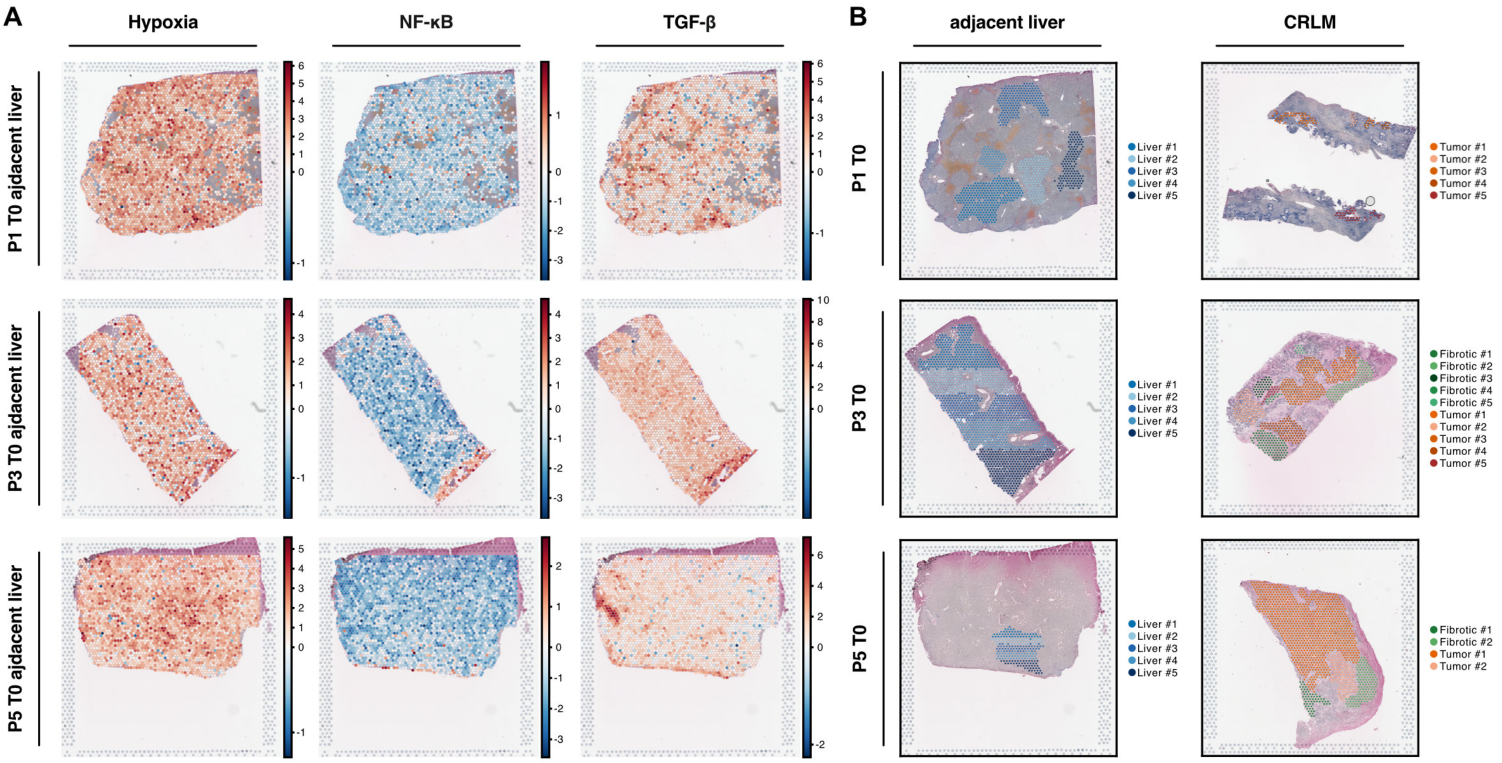

Figure S5. Spatial representation of Progeny activities and pathologist annotations

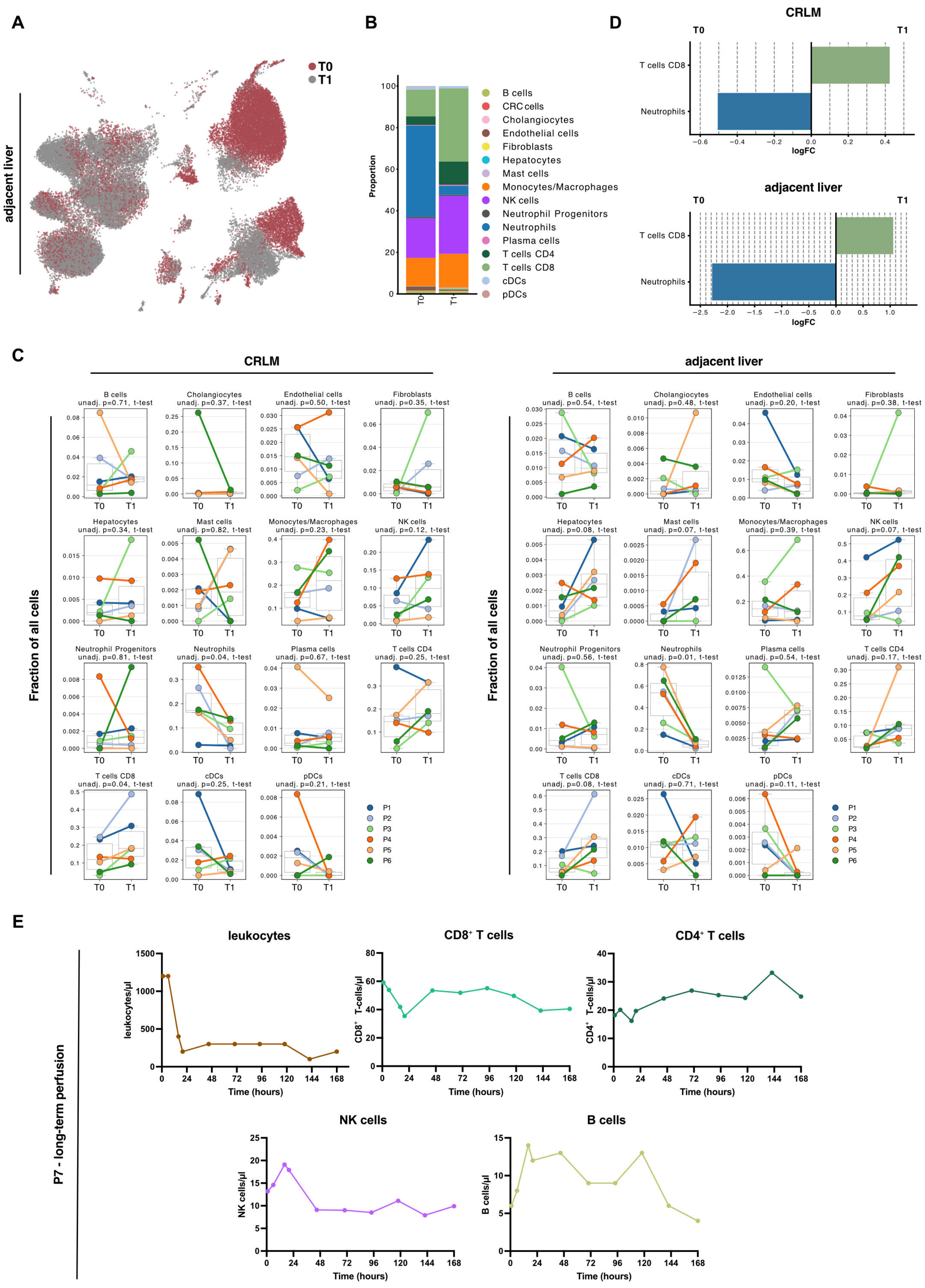

Figure S6. Compositional impact of NMP on cell types in CRLM and adjacent liver tissue

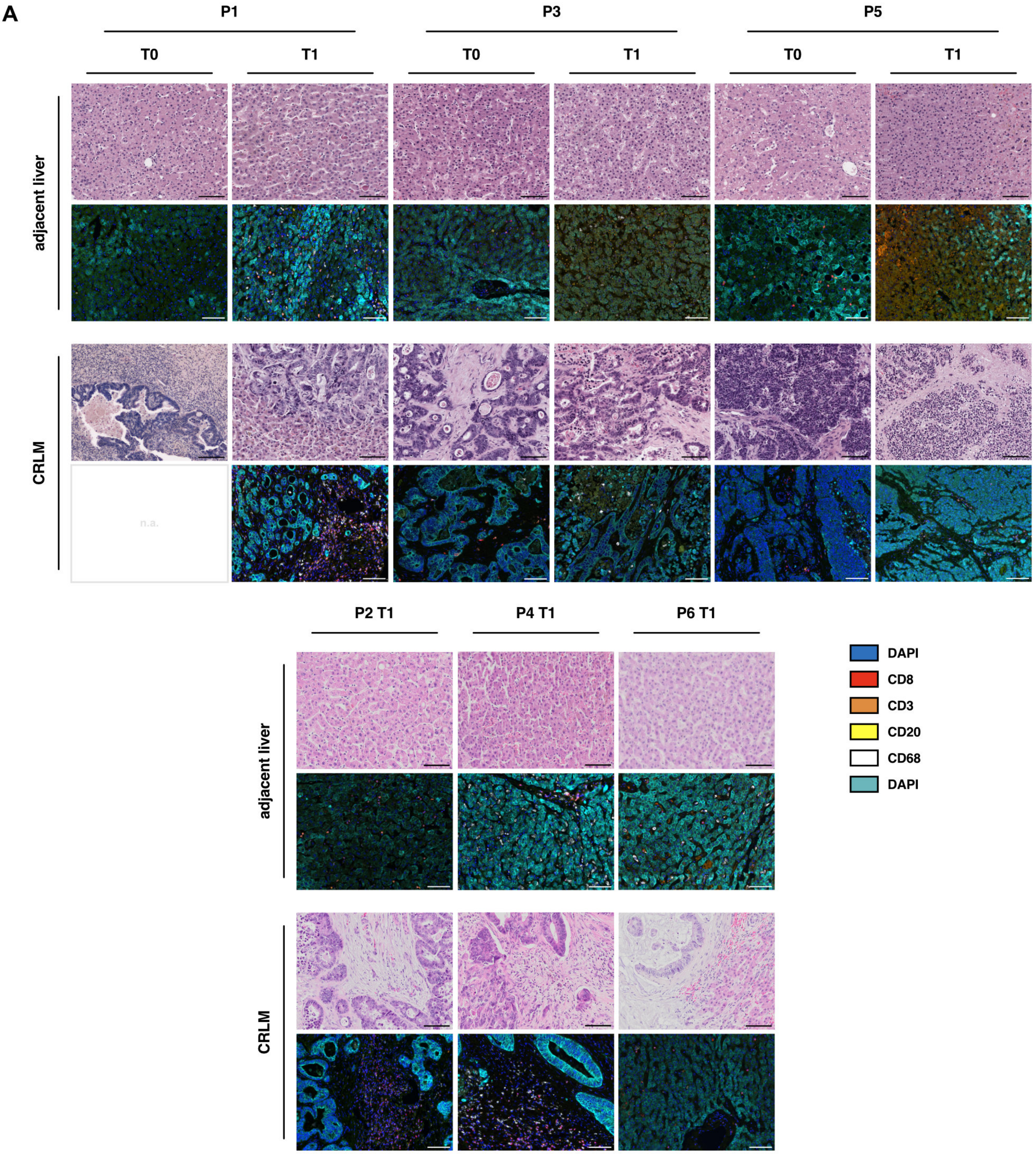

Figure S7. Tissue and TME integrity analysis throughout perfusion

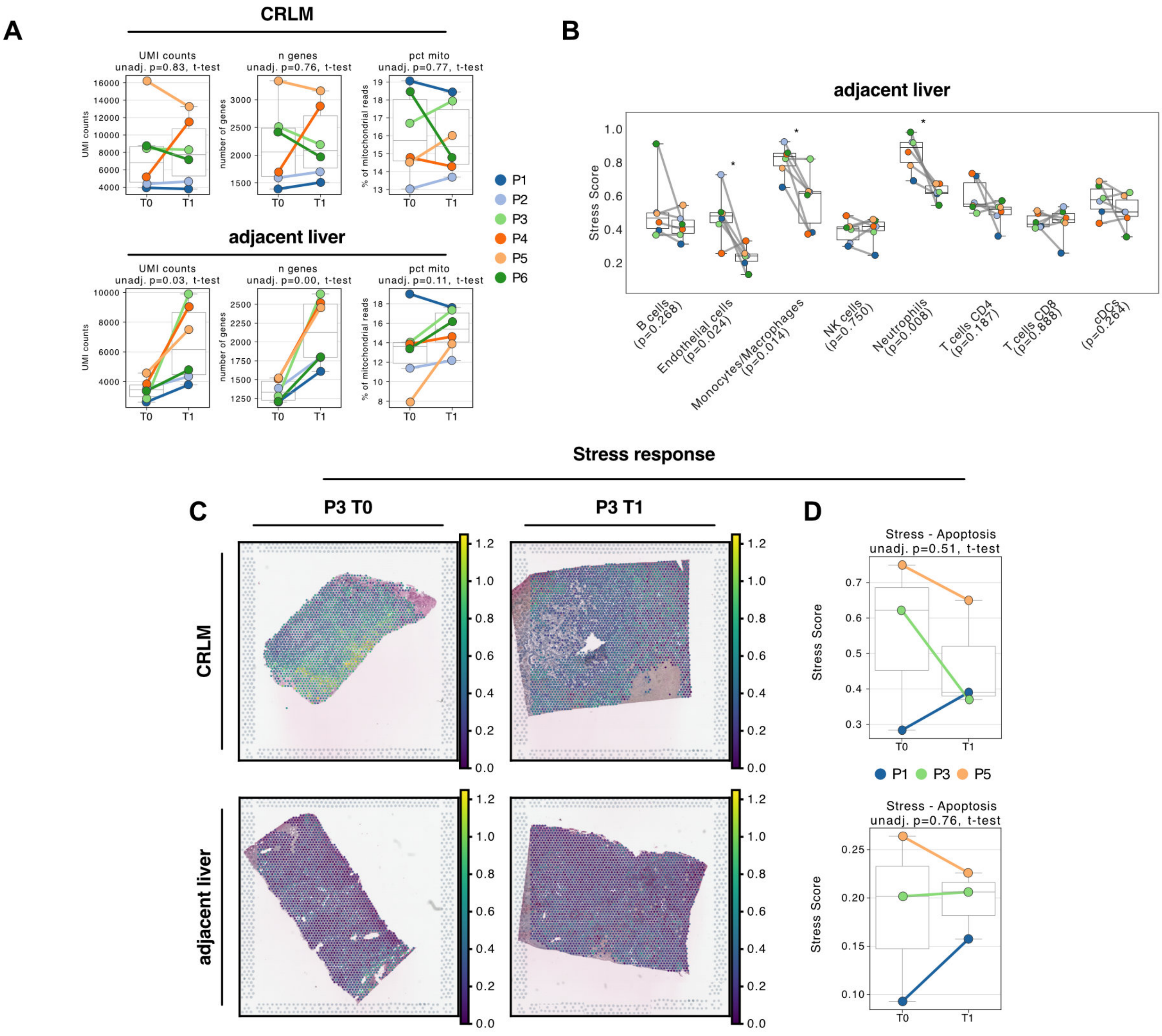

Figure S8. Impact of NMP on cellular stress

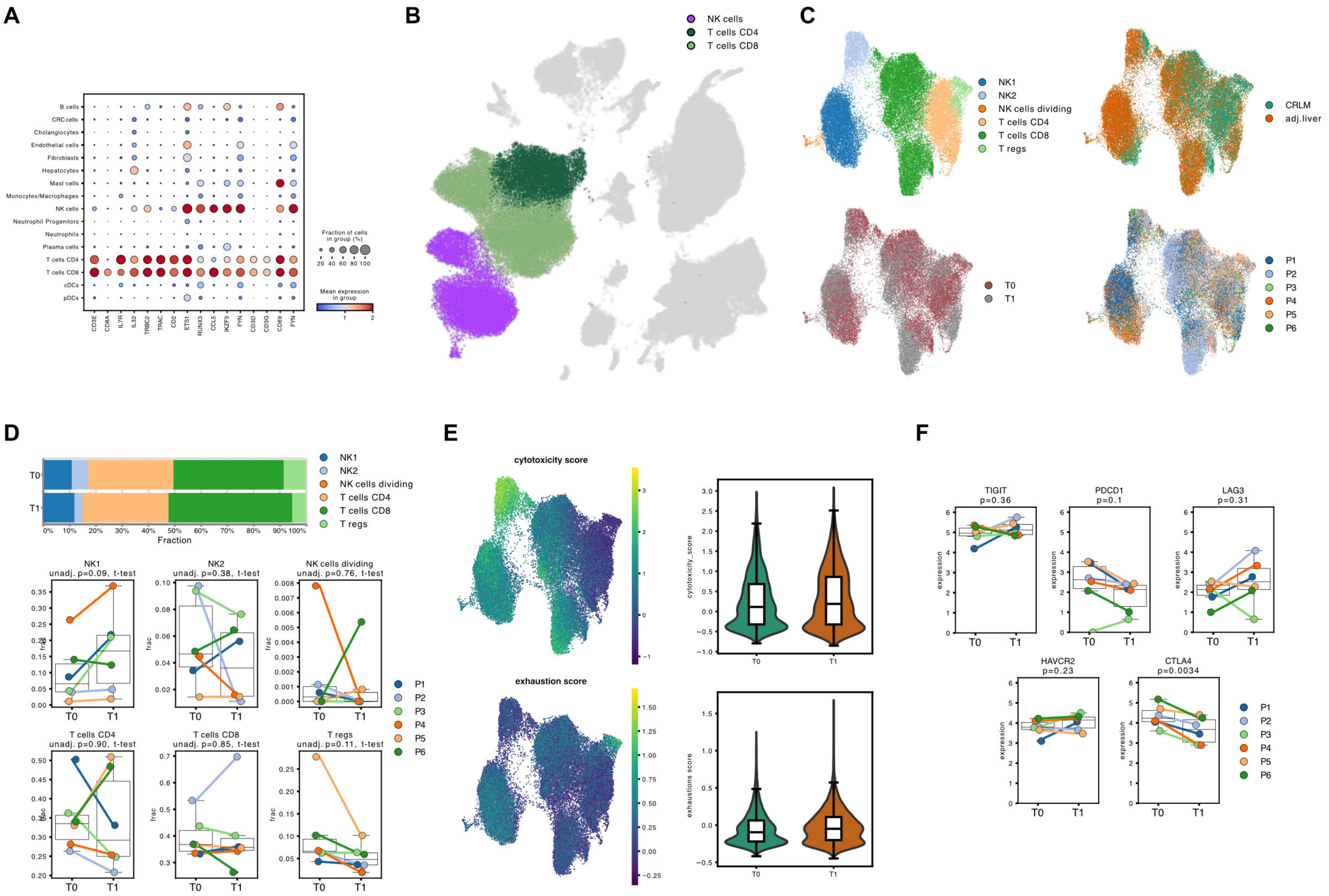

Figure S9. Detailed phenotyping of T- and NK cells stability during NMP

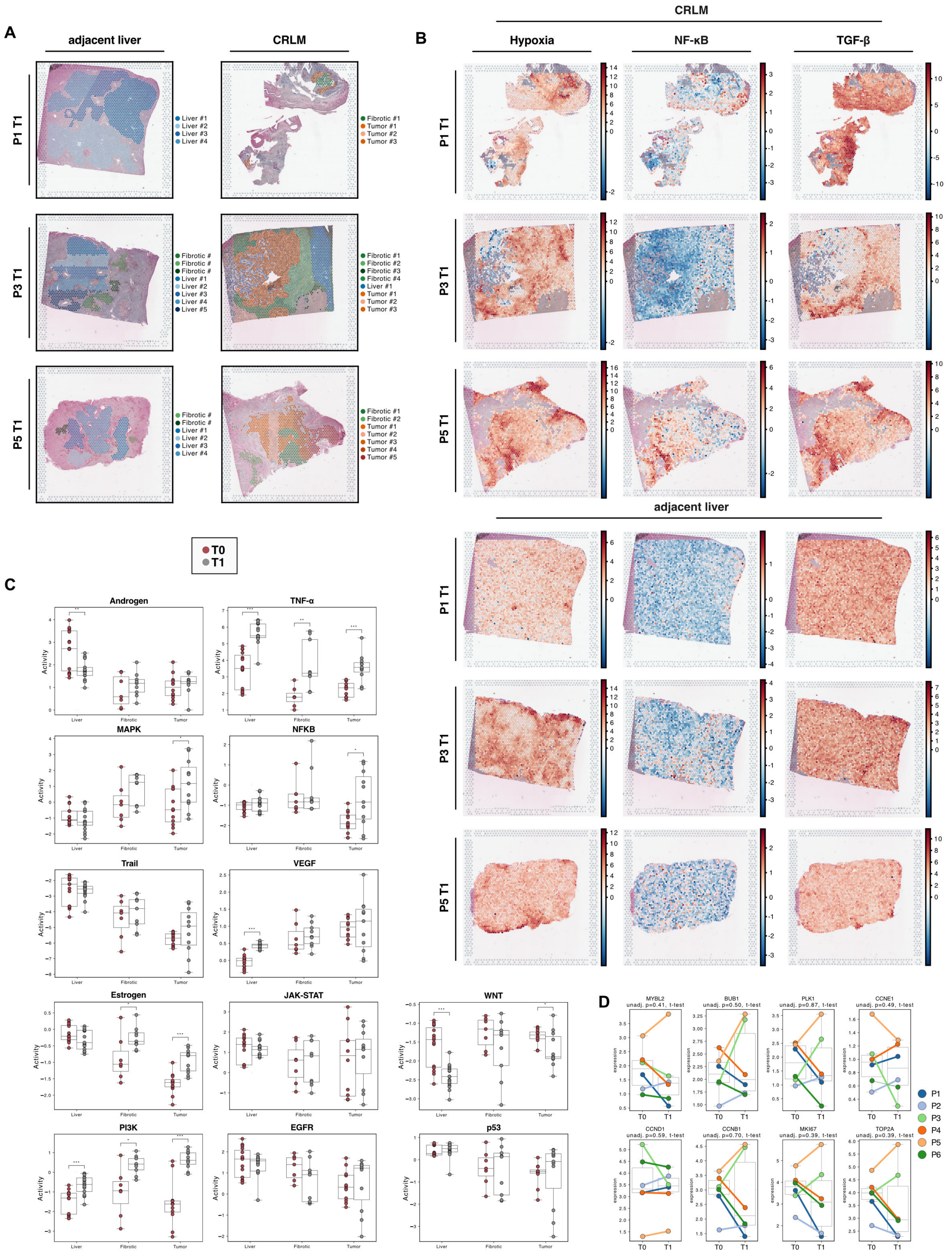

Figure S10. Impact of NMP on pathway activities within tissues

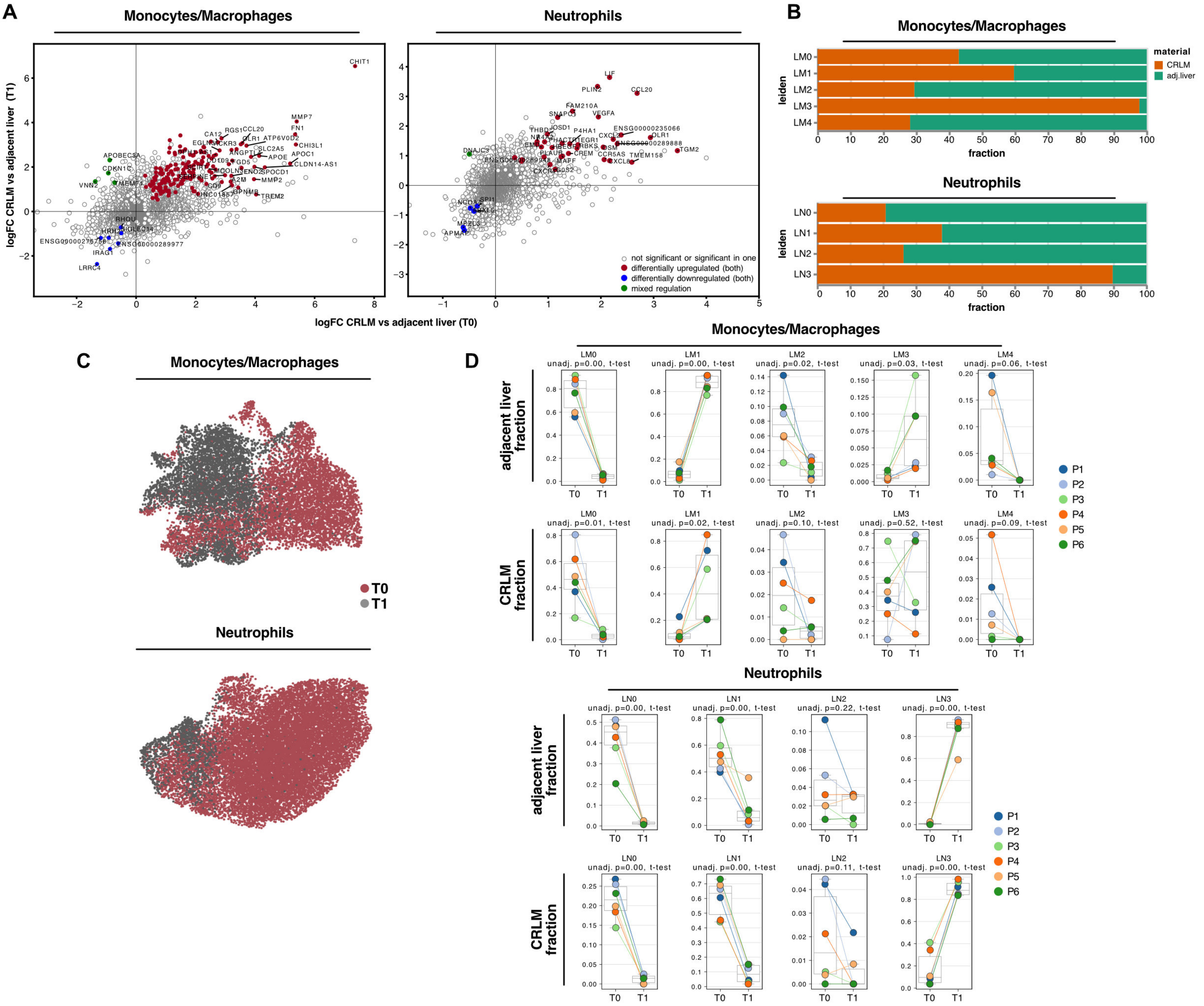

Figure S11. Impact of NMP on monocytes/macrophages and neutrophils
